# Effects of guaranteed basic income interventions on poverty‐related outcomes in high‐income countries: A systematic review and meta‐analysis

**DOI:** 10.1002/cl2.1414

**Published:** 2024-06-16

**Authors:** Anita Rizvi, Madeleine Kearns, Michael Dignam, Alison Coates, Melissa K. Sharp, Olivia Magwood, Patrick R. Labelle, Nour Elmestekawy, Sydney Rossiter, Ali A. A. Al‐Zubaidi, Omar Dewidar, Leanne Idzerda, Jean Marc P. Aguilera, Harshita Seal, Julian Little, Alba M. Antequera Martín, Jennifer Petkovic, Janet Jull, Lucas Gergyek, Elizabeth Tanjong Ghogomu, Beverley Shea, Cristina Atance, Holly Ellingwood, Christina Pollard, Lawrence Mbuagbaw, George A. Wells, Vivian Welch, Elizabeth Kristjansson

**Affiliations:** ^1^ School of Psychology, Faculty of Social Sciences University of Ottawa Ottawa Ontario Canada; ^2^ Co‐Operative Development Foundation Vineland Ontario Canada; ^3^ Telfer School of Management University of Ottawa Ottawa Ontario Canada; ^4^ Department of Public Health & Epidemiology, School of Population Health RCSI University of Medicine and Health Sciences Dublin Ireland; ^5^ Bruyère Research Institute Ottawa Ontario Canada; ^6^ Interdisciplinary School of Health Sciences University of Ottawa Ottawa Ontario Canada; ^7^ Library University of Ottawa Ottawa Ontario Canada; ^8^ Faculty of Social Sciences University of Ottawa Ottawa Ontario Canada; ^9^ School of Medicine University College Cork Cork Ireland; ^10^ Temerty School of Medicine University of Toronto Toronto Ontario Canada; ^11^ Centre for Global Health Research University of Ottawa Ottawa Ontario Canada; ^12^ Department of Surgery, Faculty of Medicine McGill University Markham Canada; ^13^ Department of Epidemiology & Community Medicine University of Ottawa Ottawa Ontario Canada; ^14^ Biomedical Research Institute Sant Pau Hospital de la Santa Creu i Sant Pau Barcelona Spain; ^15^ Bruyère Research Institute University of Ottawa Ottawa Ontario Canada; ^16^ School of Rehabilitation Therapy Queen's University Kingston Ontario Canada; ^17^ Department of Psychology Wilfrid Laurier University Waterloo Ontario Canada; ^18^ Department of Epidemiology and Community Medicine University of Ottawa Ottawa Ontario Canada; ^19^ Department of Psychology Carleton University Ottawa Ontario Canada; ^20^ School of Population Health Curtin University Bentley Western Australia Australia; ^21^ Department of Health Research Methods, Evidence and Impact (HEI) McMaster University Hamilton Ontario Canada; ^22^ School of Epidemiology and Public Health University of Ottawa Ottawa Ontario Canada; ^23^ Methods Centre, Bruyère Research Institute Ottawa Ontario Canada

**Keywords:** developed countries, food insecurity, guaranteed basic income, high-income countries, income support, mental health, multidimensional poverty measure, negative income tax, official poverty measure, poverty, social assistance, stigma, universal basic income, welfare, workfare, working poor

## Abstract

**Background:**

High‐income countries offer social assistance (welfare) programs to help alleviate poverty for people with little or no income. These programs have become increasingly conditional and stringent in recent decades based on the premise that transitioning people from government support to paid work will improve their circumstances. However, many people end up with low‐paying and precarious jobs that may cause more poverty because they lose benefits such as housing subsidies and health and dental insurance, while incurring job‐related expenses. Conditional assistance programs are also expensive to administer and cause stigma. A guaranteed basic income (GBI) has been proposed as a more effective approach for alleviating poverty, and several experiments have been conducted in high‐income countries to investigate whether GBI leads to improved outcomes compared to existing social programs.

**Objectives:**

The aim of this review was to conduct a synthesis of quantitative evidence on GBI interventions in high‐income countries, to compare the effectiveness of various types of GBI versus “usual care” (including existing social assistance programs) in improving poverty‐related outcomes.

**Search Methods:**

Searches of 16 academic databases were conducted in May 2022, using both keywords and database‐specific controlled vocabulary, without limits or restrictions on language or date. Sources of gray literature (conference, governmental, and institutional websites) were searched in September 2022. We also searched reference lists of review articles, citations of included articles, and tables of contents of relevant journals in September 2022. Hand searching for recent publications was conducted until December 2022.

**Selection Criteria:**

We included all quantitative study designs except cross‐sectional (at one timepoint), with or without control groups. We included studies in high income countries with any population and with interventions meeting our criteria for GBI: unconditional, with regular payments in cash (not in‐kind) that were fixed or predictable in amount. Although two primary outcomes of interest were selected a priori (food insecurity, and poverty level assessed using official, national, or international measures), we did not screen studies on the basis of reported outcomes because it was not possible to define all potentially relevant poverty‐related outcomes in advance.

**Data Collection and Analysis:**

We followed the Campbell Collaboration conduct and reporting guidelines to ensure a rigorous methodology. The risk of bias was assessed across seven domains: confounding, selection, attrition, motivation, implementation, measurement, and analysis/reporting. We conducted meta‐analyses where results could be combined; otherwise, we presented the results in tables. We reported effect estimates as standard mean differences (SMDs) if the included studies reported them or provided sufficient data for us to calculate them. To compare the effects of different types of interventions, we developed a GBI typology based on the characteristics of experimental interventions as well as theoretical conceptualizations of GBI. Eligible poverty‐related outcomes were classified into categories and sub‐categories, to facilitate the synthesis of the individual findings. Because most of the included studies analyzed experiments conducted by other researchers, it was necessary to divide our analysis according to the “experiment” stage (i.e., design, recruitment, intervention, data collection) and the “study” stage (data analysis and reporting of results).

**Main Results:**

Our searches yielded 24,476 records from databases and 80 from other sources. After screening by title and abstract, the full texts of 294 potentially eligible articles were retrieved and screened, resulting in 27 included studies on 10 experiments. Eight of the experiments were RCTs, one included both an RCT site and a “saturation” site, and one used a repeated cross‐sectional design. The duration ranged from one to 5 years. The control groups in all 10 experiments received “usual care” (i.e., no GBI intervention). The total number of participants was unknown because some of the studies did not report exact sample sizes. Of the studies that did, the smallest had 138 participants and the largest had 8019. The risk of bias assessments found “some concerns” for at least one domain in all 27 studies and “high risk” for at least one domain in 25 studies. The risk of bias was assessed as high in 21 studies due to attrition and in 22 studies due to analysis and reporting bias. To compare the interventions, we developed a classification framework of five GBI types, four of which were implemented in the experiments, and one that is used in new experiments now underway. The included studies reported 176 poverty‐related outcomes, including one pre‐defined primary outcome: food insecurity. The second primary outcome (poverty level assessed using official, national, or international measures) was not reported in any of the included studies. We classified the reported outcomes into seven categories: food insecurity (as a category), economic/material, physical health, psychological/mental health, social, educational, and individual choice/agency. Food insecurity was reported in two studies, both showing improvements (SMD = −0.57, 95% CI: −0.65 to −0.49, and SMD = −0.41, 95% CI: −0.57 to −0.26) which were not pooled because of different study designs. We conducted meta‐analyses on four secondary outcomes that were reported in more than one study: subjective financial well‐being, self‐rated overall physical health, self‐rated life satisfaction, and self‐rated mental distress. Improvements were reported, except for overall physical health or if the intervention was similar to existing social assistance. The results for the remaining 170 outcomes, each reported in only one study, were summarized in tables by category and subcategory. Adverse effects were reported in some studies, but only for specific subgroups of participants, and not consistently, so these results may have been due to chance.

**Authors' Conclusions:**

The results of the included studies were difficult to synthesize because of the heterogeneity in the reported outcomes. This was due in part to poverty being multidimensional, so outcomes covered various aspects of life (economic, social, psychological, educational, agency, mental and physical health). Evidence from future studies would be easier to assess if outcomes were measured using more common, validated instruments. Based on our analysis of the included studies, a supplemental type of GBI (provided along with existing programs) may be effective in alleviating poverty‐related outcomes. This approach may also be safer than a wholesale reform of existing social assistance approaches, which could have unintended consequences.

## 1 PLAIN LANGUAGE SUMMARY

### 1.1 Limited evidence that a guaranteed basic income improves poverty‐related outcomes compared to existing conditional social assistance

Numerous types of guaranteed basic income (GBI) have been tested in high‐income countries, yielding limited evidence for each type. Full GBI with gradual withdrawal of benefits (as other income increases) may reduce food insecurity and increase the time youths stay in school after the compulsory age. Supplemental GBI (paid along with existing income assistance programs) may reduce mental distress. GBI with benefit amounts larger than social assistance benefits may improve life satisfaction and subjective financial well‐being. The evidence for any type of GBI pertaining to overall health is inconclusive.

### 1.2 What is this review about?

Existing social assistance programs are complex and costly to administer. Recent reforms intended to alleviate poverty by transitioning benefit recipients to paid work through strict requirements and sanctions have not been successful and have sometimes exacerbated poverty and stigma. GBI has been proposed as a simpler solution, but opponents argue that it would be unaffordable (due to benefits being given to more people) and might result in increased poverty for people who are presently eligible for more than one program.

This review examined experiments that intended to predict the impacts of a real world GBI program. We looked at interventions that met our criteria for GBI: unconditional (e.g., not requiring job seeking), paid in cash (e.g., not food vouchers), as well as paid regularly and in fixed or predictable amounts.

Because social assistance programs are intended to alleviate poverty, we looked at any poverty‐related outcomes that the GBI experiments examined, including food insecurity, health (mental and physical), and financial, educational, and social impacts.

### 1.3 What is the aim of this review?

This Campbell systematic review synthesizes the findings of twenty‐seven studies from high‐income countries on the effects of GBI interventions on poverty‐related outcomes, compared to “usual care” (including existing social assistance for those who were eligible).

### 1.4 What studies are included?

This review summarizes evidence from 27 studies that analyzed data from 10 experiments carried out between 1968 and 2020 that aimed to explore how GBI would impact people with low incomes. Five experiments took place in the US, two were in Canada, and the rest were in Barcelona (Spain), Finland, and the Netherlands. Nine of the experiments randomly assigned participants to either receive GBI or not (control group) so that observed effects could be attributed to receiving GBI. However, the experiments and the related studies had several other methodological weaknesses, which reduced the quality of the evidence.

### 1.5 What are the main findings of this review?

Analysis of the ten GBI experiments identified four distinct types of GBI that were tested, which formed a framework to categorize and synthesize the findings in the examined studies. A fifth type of GBI, which has been implemented in new experiments that are underway, was also included in the framework to assist in future analyses.

The included studies examine a total of 176 poverty‐related outcome variables. Combining quantitative results across studies is only possible if the same variable is being measured. Therefore, most of the findings are synthesized by sorting them according to seven outcome categories and 34 subcategories.

Food insecurity, self‐rated mental distress, and post‐mandatory school enrollment may be significantly improved when GBI is provided. The evidence on subjective financial well‐being and self‐rated life satisfaction appears mixed; however, GBI with more generous benefits than existing social assistance appears to yield beneficial results for both of these outcomes.

A supplemental type of GBI, provided in addition to existing social assistance benefits, appears to improve subjective financial well‐being, self‐rated life satisfaction, and self‐rated mental distress.

Receipt of a full GBI (which replaces social assistance benefits) does not appear to improve self‐rated overall health.

### 1.6 What are the implications for research and policy?

Although the details of how a full‐scale GBI would be implemented have been discussed and debated, the empirical evidence from GBI experiments has been previously assessed as if GBI were a singular type of intervention. The typology developed for this review may assist in the evaluation and synthesis of GBI studies in the future, since numerous new experiments are underway.

The main obstacle to a more complete synthesis in this review was the large number of different outcome measures that studies used. In part, this is because income‐support interventions affect many aspects of life (social, economic, health, etc.); however, it may help if researchers use standard validated measures in future studies. At the same time, very broad outcome measures (e.g., general health) may obscure more specific impacts (e.g., on hypertension), so a compromise between general and granular outcomes may provide stronger evidence.

For implementing a full‐scale GBI program, a supplemental GBI may be most effective, as well as more prudent, to avoid possible unintended consequences of a broader reform of existing social assistance.

### 1.7 How up‐to‐date is this review?

Searches for eligible studies were conducted until December 2022. Studies published later would likely include data collected during the COVID‐19 pandemic, so they would not be within the scope of this review (on interventions conducted to inform permanent income assistance policies).

## 2 SUMMARY OF FINDINGS

Summary of findings.

**Table:** 

Outcomes	Anticipated absolute effects (95% CI)	№ of participants (studies)	Certainty of the evidence (GRADE)	Comments
Self‐reported food insecurity Assessed with: interview/survey Follow‐up: range 2 to 4 years	Two studies found a reduction in food insecurity. 1 RCT: calculated SMD of 0.41 (95% CI: 0.26, 0.57) 1 retrospective cohort study: calculated SMD of 0.57 (95% CI: 0.49, 0.65)	5030 (1 RCT, 1 NRS)	⊕⊕⊕◯ Moderate^a,b^	Upgraded by one level due to very large estimated effects in both studies. Pooling/meta‐analysis was not possible for this outcome because of different study designs.
Poverty level Assessed with: official/national or international poverty measures		(0 studies)	‐	None of the included studies reported this outcome.
Self‐rated overall physical health Assessed with: interview/survey Follow‐up: range 1 to 2 years	1 of 4 studies found a significant improvement, compared to controls	3169 (4 RCTs)	⊕⊕⊕◯ Moderate^c,d^	Pooling/meta‐analysis was not possible for this outcome because of different GBI interventions (2 studies provided supplemental GBI, 2 studies provided GMI with partial withdrawal).
Self‐rated mental distress Assessed with: interview/survey Follow‐up: range 1 to 2 years	Pooled SMD is 0.25 SD lower versus control group (0.37 lower to 0.13 lower)	1958 (2 RCTs)	⊕⊕◯◯ Low^e^	
Overall life satisfaction and subjective well‐being Assessed with: interview/survey Follow‐up: range 16 months to 2 years	2 of 3 studies found a significant improvement compared to controls	2844 (3 RCTs)	⊕⊕⊕◯ Moderate^d,f^	Pooling/meta‐analysis was not possible for this outcome because of different GBI interventions (1 study provided supplemental GBI, 2 studies provided GMI with partial withdrawal).
Subjective financial well‐being Assessed with: interview/survey Follow‐up: range 1 to 2 years	3 of 4 studies found significantly improved subjective (self‐reported) financial well‐being, compared to controls	3169 (4 RCTs)	⊕⊕◯◯ Low^c,g^	Pooling/meta‐analysis was not possible for this outcome because of different GBI interventions (2 studies provided supplemental GBI, 2 studies provided GMI with partial withdrawal).
Post‐mandatory school continuation Assessed with: interview/survey or educational system records Follow‐up: range 2 to 4 years	4 of 5 studies found significantly higher post‐mandatory school enrollment (after age 16 or 17) for youth from families receiving the intervention, versus control groups. 1 study found a larger effect for boys, 1 study found a larger effect for girls. The other studies did not report results by sex or gender.	Total *N* not available (4 RCTs, 1 NRS)	⊕⊕⊕◯ Moderate^h,i^	Upgraded by one level due to large estimated effect in 3 of 5 studies. 2 studies used school board data, 3 studies used interview/survey data. Pooling/meta‐analysis was not possible for this outcome because sample sizes were not reported by study group (2 studies reported the total sample sizes (*N*), 2 studies reported the number of families in the experiment, 1 study reported the total number of observations).

^a^The retrospective study (using health system records) had a low risk of bias, the RCT had a high risk of bias due to potentially high measurement and analysis biases.

^b^Interventions delivered differently and in different settings.

^c^1 study with moderate overall risk of bias, 2 studies with high risk of bias in 2 of 7 domains, 1 study with high risk in 4 of 7 domains.

^d^There is considerable heterogeneity among the results; however, this is likely due to differences in the interventions (i.e., GBI benefits were of various monetary amounts or different “doses”) so we did not rate down for unexplained inconsistency.

^e^1 study with high risk in 2 of 7 domains, 1 study with high risk in 4 of 7 domains.

^f^1 study with moderate overall risk of bias, 2 studies with high risk of bias in 2 of 7 domains.

^g^High degree of heterogeneity (Chi² = 26.89 (*p* < 0.00001), *I*² = 93%).

^h^2 studies with high risk in 2 of 7 domains, 1 study with high risk in 3 of 7 domains, 2 studies with high risk in 6 of 7 domains.

^i^Sample sizes were not reported by study group (n) or at all (unreported sample size, *N*). Two studies gave total sample sizes that were not large (*N* = 138 and *N* = 266).

## 3 BACKGROUND

### 3.1 The problem, condition or issue

#### 3.1.1 Poverty in high‐income countries

Although the concept of poverty in high‐income countries seems like a contradiction in terms, there are nonetheless many people in these countries who are unable to afford basic needs such as adequate and nutritious food. Many of these people rely on social assistance benefits and food banks, as well as housing, heating, and electricity subsidies to make ends meet. The seeming incongruity of poverty existing in wealthy countries can be explained in part by the definition of a high‐income country: one that has a gross national income (GNI) per capita of US$13,846 or more (World Bank, 2023). Because this criterion is only an average for each country, it does not provide any information on the distribution of the income within the population or indicate how many of its citizens are unable to afford a basic standard of living.

Although it is expected that some people in the free‐market economies of high‐income countries will earn more money than others, income inequality has increased in almost all developed (industrially advanced) countries since 1990 (United Nations, 2020). In the U.S., the share of national aggregate income held by low‐income households fell slightly from 10% to 9% between 1970 and 2018, while the share held by high‐income households over the same time increased from 29% to 48% (Horowitz, 2020). As well, the proportion of the population in the middle‐income class (having household incomes between 75% and 200% of the national median) has declined since the mid‐1980s in most developed countries, while the size of the lower‐income class (below 75% of the national median household income) has grown in most (OECD, 2019). In contrast, due to strong economic growth in industrially developing countries in the last two decades, the size of the middle class in these countries has nearly doubled or tripled, depending on the measure used (Versace, 2021). One factor in these diverging trends between higher‐income and lower‐income countries is the outsourcing of manufacturing by developed countries in recent decades, combined with technological advancement that has displaced routine‐based jobs, as well as increasing computing power and artificial intelligence which is also placing nonroutine jobs at risk (OECD, 2021).

According to the International Labor Organization (ILO), 22% of people in developed countries (more than 300 million) were considered poor in 2012, with an income of less than 60% of the national median – and since then, various indicators have shown poverty rates to be either unchanged or, in the case of some countries (e.g., Greece, Italy, Portugal), trending higher after the 2008 global financial crisis (ILO, 2016; OECD, 2022). Similarly, the poverty threshold of the Organization for Economic Co‐operation and Development (OECD), set at 50% of the national household median income, indicates that poverty rates in developed countries have remained fairly stable between 2008 and 2019, ranging in 2019 from 5.6% in Czechia (Czech Republic) to 18% in the United States (OECD, 2023). The OECD data also show the poverty rate for children (0–17 years old) in the United States and Spain to be the highest among developed countries in 2019, at 21%. (Although poverty rates are also available for 2020 and later, we are not including them for comparison because of the economic instability caused by the recent pandemic and the various temporary relief measures that were implemented in each country.)

It is important to note that the poverty rates cited above draw on measures of relative poverty (based on national median incomes) and not absolute poverty, which refers to the extent of material deprivation and the lack of access to food, clean water, health services, and basic education (Peer, 2023). The implications of relative poverty can vary from setting to setting. For example, if one country's median income is €30,000 and another's is €40,000, then using a poverty threshold of 50% of the median income would mean that people with incomes between €15,000 and €20,000 would be considered poor in one country but not in the other. Relative poverty measures can also be misleading if economic conditions change in the short term (Sarlo, 2018). For example, if a stock market crash resulted in lower incomes for wealthy people in a country, the national median income would decline, resulting in fewer people being considered as poor without any change in their financial situations.

Relative poverty rates can provide a useful indicator of income inequality within a country (e.g., identifying what proportion of people have incomes in the lowest quartile), but they do not provide any insight into the extent or severity of poverty that people experience. It is imperative to recognize this limitation when assessing the effectiveness of policies, programs, or interventions intended to reduce poverty, considering that basic material needs such as food and shelter are unmet – either temporarily or chronically – for many people in high‐income countries. Because homelessness involves complex underlying factors besides not being able to afford housing, such as substance use/addiction, intimate partner violence, and mental illness, this experience of poverty is outside the scope of this review, but has been addressed in others (e.g., Aubry, 2020; Moledina, 2021; and Nilsson, 2019). Inadequate access to food, on the other hand, is directly related to people's financial circumstances in high‐income countries, as reflected in commonly used definitions of food insecurity: “a lack of available financial resources for food at the household level” (Hunger and Health, 2022), “[not] having physical and economic access to sufficient healthy food at all times” (UK Government, 2021), and “the inadequate or insecure access to food because of financial constraints” (Tarasuk & Mitchell, 2020). These definitions refer to “household food insecurity,” a term that is commonly used to distinguish this experience from food insecurity associated with other factors: food availability (production, supply, variety), physical access to food (distance, means of transportation, mobility), food utilization (knowledge, equipment, preparation, storage), and stability (political, macroeconomic, environmental) (Calloway, 2023; Carson, 2020; FAO, 2023).

#### 3.1.2 Policies and programs for reducing poverty

Social justice advocates have long asserted that poverty reduction is a moral obligation of the state which can be achieved by a fairer distribution of wealth (Barder, 2009; Standing, 2019). Although various types of support have been provided by the state to people in poverty since ancient times, the modern concept of social welfare emerged in the late 19th century in Germany under Chancellor von Bismarck, based on the precept that people facing poverty and distress should receive assistance from the state, not as a matter of charity but as a right (Rose, 1985). Other high‐income countries followed suit during the 20th century, implementing social assistance programs to alleviate poverty after the Great Depression (Trattner, 2007). In the United Kingdom during the Second World War, economist Sir William Beveridge wrote a report for the government which called for a “revolution” in the direction of Britain's welfare state and laid out a comprehensive set of social assistance programs, including child benefits, publicly funded healthcare, and funeral allowances. The Beveridge Report expanded on programs introduced by Lloyd George and Churchill three decades earlier and provided the blueprint for modern welfare in the United Kingdom (Day, 2017; Wheeler, 2015). Similarly, the Marsh Report of 1943 provided the foundation for the current social security system in Canada, by proposing measures similar to Beveridge's (a mentor of Marsh) and adding elements such as an employment program and health care insurance (Policy Options, 2004).

The cost of social assistance programs in high‐income countries is between 12% and 31% of the gross domestic product (GDP), depending on the country (OECD, 2020). The generosity of social assistance also varies over time, with cutbacks being common during economic recessions due to politicians being pressured to support workers not “shirkers” (Romano, 2015).

Social welfare programs were found to reduce both relative and absolute poverty in most high‐income countries between 1960 and 1991, particularly in those with more generous programs (Kenworthy, 1999). Since then, however, welfare reforms – often called “workfare” because of their emphasis on transitioning social assistance recipients into the workforce – have been blamed by critics for reversing the poverty reduction trend by cutting benefits to the unemployed, including single mothers, and requiring them to accept precarious, low‐paying jobs (Carey & Bell, 2020; Widerquist, 2013). The increased conditionality of workfare may also result in additional stigma and shame for recipients who either remain unemployed, or those who are skilled or educated and placed in low‐skill, low‐paying jobs (Carey & Bell, 2020; Widerquist, 2013). Sanctions in the form of benefit cuts and interruptions are intended to increase compliance with the conditions of workfare programs (e.g., accepting any type of available work); however, a recent review of 94 studies suggests that these sanctions can have detrimental effects on mental and physical health, debt, material hardship, and financial stress (Pattaro, 2022).

Because social assistance programs rely on a minimum income threshold to determine eligibility, transitioning to a low‐paying job with an income slightly above the threshold can result in losing the benefit. Some programs include an earnings allowance (e.g., for income from a part‐time job) which raises the eligibility threshold; however, this allowance is usually a modest amount (e.g., $200 per month in Ontario, Canada; Government of Ontario, 2022) or is conditional (e.g., having a dependent child in the UK; UK Government, 2023). Low‐paid work may also mean losing in‐kind benefits such as a rent subsidy and dental care, so a person's net income may end up being even lower than the amount provided by social assistance (Wolfson, 2018).

A distinguishing feature of social assistance in most high‐income countries is the availability of various programs, offered by different levels of government (federal, state/provincial, municipal) and targeted at specific groups (e.g., people with disabilities, women with infant children) and for specific needs (e.g., money for food or rent). This approach has been criticized as being a patchwork of programs that are confusing in terms of understanding eligibility criteria, and which fail to provide some people with a subsistence‐level income (Koebel & Pohler, 2019; Wolfson, 2018). The complexity of the programs and uncertainty regarding eligibility also translates into high levels of non‐uptake, which results in many people missing out on benefits that they are eligible to receive. Although non‐uptake results in short‐term savings for the government, it may result in more costly downstream effects if it prevents people from affording early medical treatment or paying for a better education for their children (Van Mechelen & Janssens, 2017).

The United Kingdom introduced a welfare reform called Universal Credit (UC) in 2012, which consolidates six previously separate programs (Winchester, 2021). To be eligible for UC, most recipients who are unemployed (except those with infant children) must seek work or take training courses, and noncompliance such as missing an appointment with a work coach can lead to sanctions (UK Government, 2014). Some studies also show that the reforms of UC have led to an increase in poverty for single mothers, due to the loss of provisions offered by the replaced programs, as well as large reductions in time available to care for children due to intensive job‐seeking requirements (Carey & Bell, 2020).

One type of supplementary social assistance offered in many high‐income countries is in the form of refundable (or payable) tax credits, which provide cash benefits to eligible people with low incomes who file income tax returns. However, this form of income supplement has been criticized as being insufficient, especially for people with low incomes and without children (Koebel & Pohler, 2019). Also, in countries where people have to file income tax returns to receive certain benefits, refundable tax credits only reach those who file returns, and the rate of non‐filing is as high as 20% among people with very low incomes (Robson & Schwartz, 2020).

#### 3.1.3 Universal basic income (UBI)

UBI has been proposed as a potentially effective way to alleviate poverty (Hasdell, 2020) and to replace the current assortment of social assistance programs in high‐income countries, administered by different levels of government, which have been described as bureaucratic, costly, and stigmatizing (Koebel & Pohler, 2019; Reed & Lansley, 2016). UBI is “an income paid by a political community to all its members on an individual basis, without means test or work requirement” (Van Parijs, 2004, p. 8). More recently, additional dimensions of UBI have been specified: it is paid at regular intervals and as cash payments which recipients can spend in any way they choose (BIEN, 2020). The amount of the UBI payment should also be stable and predictable (Standing, 2021). These payments should provide enough funds to meet basic needs, may or may not be phased out as earnings increase, and be available to a large portion of the population, rather than targeted to a particular group (Hoynes & Rothstein, 2019). Universality is intended to promote social cohesion; a universal guaranteed annual income (GAI) becomes a shared social experience rather than an individual benefit (Forget, 2011). Advocates of UBI programs have suggested that they are a just and economically efficient means of achieving the core objectives of social security: redistributing income, alleviating poverty, and managing risk (Martinelli, 2017).

Proponents of UBI have criticized the reformed welfare programs of the past three decades as being fiscally unsustainable, overly intrusive and inhibiting the agency of benefit recipients (Orrell, 2021). In terms of public opinion, a study in the United Kingdom and the United States found that the two main reasons cited in support of UBI were simplicity and efficiency of administration, and the reduction of stress and anxiety (Nettle, 2021).

Other important implications for UBI pertain to inequalities across socioeconomic status, race, ethnicity, and gender. Stressors such as financial difficulties, caring for disabled children or parents, and abusive relationships at work or at home have damaging effects on mental and physical health, and these effects disproportionately impact women, racial/ethnic minorities and people with low incomes (Thoits, 2010).

For women, UBI paid on an individual basis could potentially address several areas of concern. Firstly, UBI would provide an income for women who perform work outside the formal labor market, such as caring for children and doing volunteer work, as well as for those who have personal care jobs which usually do not pay well. An individual‐level UBI would also reduce the financial dependency of spouses in abusive households, who currently are not eligible for social assistance if their spouse earns an income above the eligibility threshold (Bidadanure, 2018).

Poverty rates in high‐income countries are disproportionally high for Black and Indigenous people as well as for other racial and ethnic minorities, often resulting from involuntary unemployment due to discrimination and lack of opportunities. UBI has been proposed since the 1960s by Martin Luther King, Jr., the Black Panther Party, and other advocates as a way to alleviate poverty due to systemic racism and reduce income inequality along racial lines (Bidadanure, 2019).

UBI is currently receiving renewed attention due to rising income inequality and the changing nature of work due to automation and reductions in the quantity and quality of jobs (Gentilini, 2020; Hasdell, 2020). More recently, the economic disruptions brought about by the COVID‐19 pandemic have further prompted policy discussions on full‐scale UBI programs. On the other hand, the concept of UBI is also controversial and has been criticized for potentially disincentivizing work and for being extremely costly, to the point that it could result in cuts to healthcare and education (Centre for Social Justice, 2018; Hoynes & Rothstein, 2019).

#### 3.1.4 Defining and measuring poverty

Regardless of the type of poverty reduction approach that may be implemented, a major challenge is evaluating the effectiveness of the approach. This is because a standardized method does not exist for measuring poverty. Indeed, there has been considerable debate over which poverty indicators are most accurate and reliable (Cutillo, 2020; Meyer & Sullivan, 2012). Official poverty measures (OPMs) have traditionally been based on income, setting some minimum threshold as the poverty line, while some newer official measures factor in the cost of living, or at least the cost of basic needs (Cutillo, 2020; Guio, 2016; Meyer & Sullivan, 2012). Simple income‐based measures are still commonly used and have been criticized as being outdated and that they measure income inequality, not poverty (Gupta & Theoharis, 2020; Konle‐Seidl, 2021). The OECD, for example, defines the poverty line as “half the median household income of the total population” in each country (OECD, 2021). Because of the arbitrary thresholds of such measures, millions of people slightly above the poverty line may live precariously – “just a $400 emergency away from poverty” (Gupta & Theoharis, 2020).

Consumption‐based measures, which use surveys to assess what goods and services individuals or households consume, have been proposed as a more accurate indicator of poverty. A comparison of various poverty measures in Europe found that consumption‐based measures versus income‐based measures identified different groups as being poor, and that income had a low correlation with severe material deprivation (Cutillo, 2020). Similarly, a comparison of poverty measures in the United States, including the OPM, found that a consumption‐based measure was more accurate in identifying people who were facing financial hardship – that is, low consumption was a better indicator than low income (Meyer & Sullivan, 2012). Consumption‐based measures can also identify those with incomes above the official poverty line who spend a large amount on health‐related expenses, which may cause difficulty in affording food and rent (Sarabia, 2016).

The inaccuracy of income‐based poverty measures, even when the cost of living is factored in, can be demonstrated by nonmonetary indicators of poverty. For example, in Canada the OPM, the Market Basket Measure (MBM), indicates that the percentage of Canadians living below the poverty line decreased considerably, from 15.0% in 2012 to 10.1% in 2019. Over almost the same period, however, the prevalence of food insecurity increased slightly, from 8.3% of households in 2011–2012 to 8.7% in 2017–2018 (Statistics Canada, 2021). As well, the number of people aged 65 and older who visited food banks because they did not have enough money for food increased by 29.8% between 2016 and 2019 (Food Banks Canada, 2019). OPMs also may not capture the impacts of food poverty on children, for whom food insecurity is not only associated with hunger and inadequate nutrition, but also with social, developmental and health impacts that may persist into adulthood (Ramsey, 2011; Thomas, 2019).

Food insecurity has been proposed as a more accurate and sensitive indicator of poverty than measures based on income and estimates of the cost of living (Loopstra & Tarasuk, 2013; Power, 2016). Loopstra and Tarasuk observed a linear relationship between the severity of food insecurity and the odds of experiencing hardships such as not being able to pay rent and bills on time. The increasing use of food banks in high‐income countries is also an important indicator in relation to deeper poverty, because the people who rely on food banks for assistance are typically in the most food‐insecure categories (moderately or severely food‐insecure) and have lower incomes than food‐insecure people who do not rely on food banks (Tarasuk, 2020).

To examine the relationships between various types of material deprivation, Toppenberg (2017) constructed regression models using data from the US Census Bureau's 2015 Current Population Survey Food Security Supplement, and found that compromised health, education, standard of living, and housing were all better predictors of food insecurity than low income.

Recently, there has been increasing attention in social sciences and policy research to the multidimensional nature of poverty, which includes income poverty and material deprivation, as well as the psychological dimension of subjective financial stress (Schenck‐Fontaine & Panico, 2019). The experience of poverty also includes other less tangible aspects which income and consumption measures are not able to capture, such as deficits in the areas of “voice, human security, isolation, dignity, lack of time, and subjective wellbeing” (Poverty Analysis Discussion Group, 2012, p. 5).

Interestingly, multidimensional poverty indices have been adopted in many developing countries as OPMs, incorporating the dimensions mentioned above, as well as: basic services, environment, personal safety from violence, and social inclusion (ITWG, 2021). Nongovernmental bodies such as the United Nations Development Program (UNDP) and the International Fund for Agricultural Development (IFAD) have also developed multidimensional poverty measures, as has the United Nations Children's Fund (UNICEF) to assess the poverty of children (SDSN, 2019).

The European Union (EU) adopted a new OPM in 2010 which is described as multidimensional (SDSN, 2019; Whelan, 2014); however, it only includes three indicators: relative income (60% of the national median), employment, and material deprivation.

The development of various types of poverty measures in recent decades reflects the evolution in the definitions of poverty over this time. Traditional definitions refer to the inadequacy of income and assets, defining poverty as “the state of one who lacks a usual or socially acceptable amount of money or material possessions” (Merriam‐Webster, 2024) or “poverty is said to exist when people lack the means to satisfy their basic needs” (Encyclopædia Britannica, 2024). More recent definitions have included the additional dimensions of poverty described above. At the present time, however, a standard or widely accepted definition that describes poverty as it exists in high‐income countries has not been established. Because of this, it is still not clear which poverty measures are best able to assess the extent and severity of poverty in high‐income countries.

In this review, we examine basic income interventions for reducing poverty. Due to the evolving nature of the definitions and measures of poverty, all indicators were considered. Thus, we sought evidence obtained using traditional income‐based poverty measures as well as alternative and novel measures – based on food insecurity, consumption, material deprivation, subjective financial stress, and other physical, social, and psychological dimensions of poverty – to examine and compare the effectiveness of different variants of a GBI.

### 3.2 The intervention

A true UBI policy has never been implemented in high‐income countries (Gentilini, 2020; Gibson, 2020). Thus, our review examines basic income interventions which include some features of UBI, as described below. These quasi‐UBI approaches are known by various terms such as: basic income guarantee (BIG), guaranteed annual income (GAI), unconditional cash transfer (UCT), and negative income tax (NIT). These variations share the common attribute of monetary benefits that would be guaranteed by the state (Van Parijs & Vanderborght, 2017), so we will use the term “guaranteed basic income” (GBI) in this review to cover all types of basic income interventions. The shorter term “basic income” is also often used in the literature as a short form of “universal basic income”; therefore, we will use the term “guaranteed basic income” (GBI) to avoid confusion. For the meaning of basic, we will use the two interpretations outlined by Hoynes and Rothstein (Hoynes & Rothstein, 2019): (1) an amount sufficient to pay for one's basic needs, or (2) an amount given to each recipient that provides a base which can be supplemented by other forms of income.

We also define the “regular” and “predictable” payment criteria of GBI as being paid at least once per year and in the same amount each time (in real value, adjusted for inflation). Although not always considered a core criterion of a basic income, we consider regular payments of a fixed or predictable amount to be essential if GBI is used as an intervention to reduce poverty. Not knowing if the next payment will cover the same expenses as the previous one may cause anxiety and apprehension for the recipient, which could aggravate the experience of poverty. Because some programs, often described as a type of basic income, are based on dividends which change in amount over time (e.g., from oil or casino revenues), we did not consider them to be a type of GBI according to our criteria of providing stable or predictable amounts.

**Figure 1:**
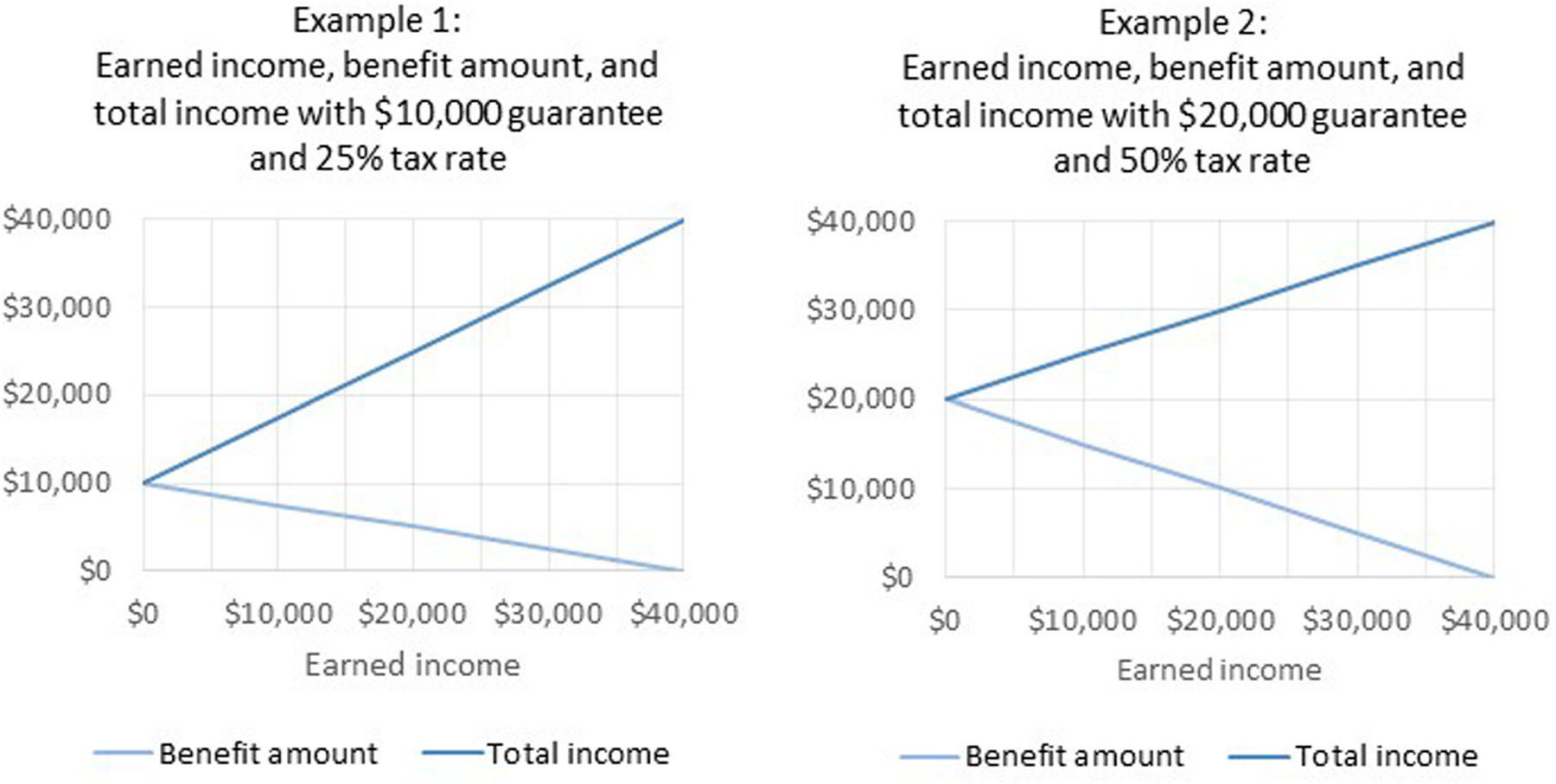
Examples of two negative income tax variations.

One form of GBI is an NIT, also called reverse income tax, whereby people whose income falls below some threshold would receive money from the government – that is, the money flows in the negative or reverse direction compared to regular income tax. In this approach, a certain benefit amount is provided by the government if there is no earned or other income, and then the benefit is reduced (or “taxed” away) as earned income rises. In this way, one's total income would never be below the “guarantee” amount that the particular NIT plan provides. Figure 1 illustrates two examples of income with the NIT approach, showing how the benefit is reduced as earned income increases in each scenario. The income at which the benefit amount becomes zero is referred to as the “break‐even” point, and in both of these examples it is $40,000. This amount was chosen to illustrate one limitation of the NIT approach: while the lower tax rate of 25% provides a greater work incentive than the 50% tax rate, the more gradual withdrawal of benefits means that the guarantee amount has to be set lower (half as much in this case), so people who don't have other sources of income may receive less than they would from existing social assistance.

The withdrawal rate or “tax” rate on benefits in the NIT approach is alternatively referred to as a “take‐back” rate, “claw‐back” rate, benefit reduction rate, or “taper” rate (due to the tapering‐off of the benefit level as other income increases). Some other forms of GBI, outlined below in the results section, also have a take‐back condition in the intervention whereby the benefit is reduced at a known, prescribed rate when there is additional income from employment or other sources; however, to be considered as GBI, the intervention must provide a minimum income guarantee which is not conditional on fulfilling requirements such as job seeking, training, or meeting regularly with caseworkers. Although the benefit amount of a GBI with a benefit reduction structure is determined by the amount of other income, and in that sense is conditional, the provision of the guaranteed minimum income (GMI) amount is not. This unconditional income guarantee serves to differentiate studies of GBI included in this review from those of existing social assistance programs, including those with “soft” (minimal) eligibility conditions.

In summary, we included interventions that meet the following criteria: (1) regular payment intervals, (2) paid in cash (not in‐kind), (3) a guaranteed minimum amount received unconditionally (up to some income level), and (4) paid in fixed or predictable amounts.

### 3.3 How the intervention might work

Proponents of GBI suggest that it is a preferable way to relieve poverty than conventional welfare programs for several reasons:

1. GBI would avoid the stigmatization inherent in conditional, intrusively means‐tested programs by offering the benefit to everyone within a community or at least everyone below a certain income threshold (Gentilini, 2020; Jenkins, 2019).
2. The means testing of applicants and scrutiny of recipients in welfare programs is labor‐intensive. These procedures are not necessary with GBI; thus, it would be a more efficient method of poverty reduction (Widerquist, 2013; Yang, 2021).
3. GBI is a matter of social justice which addresses growing income inequality and fosters a fairer sharing of the public wealth accumulated over successive generations (Gentilini, 2020; Standing, 2021).

One drawback of welfare programs is that not everyone who is eligible ends up receiving the benefit. Many people do not apply for assistance because of the stigma and shame associated with relying on social assistance, while others may not realize they are eligible for specific programs because of the complex requirements and procedures for enrollment (Bidadanure, 2019; Gentilini, 2020). Take‐up rates for many programs in Europe and the US are below 50%, due in part to information barriers for potential recipients, but even more so due to limited administrative functioning and communications strategies – the improvement of which would require even higher administrative costs (Laín & Julià, 2022). Another limitation of some government programs is that they are targeted toward specific populations (e.g., families with children), so other people do not qualify for assistance (Koebel & Pohler, 2019). Because everyone in the community with incomes below some threshold would be eligible for GBI, these problems would be avoided since everyone with a low income or no income would be entitled to receive the benefit.

As noted above, analyses of poverty measures based on income have found that they may not be accurate indicators of poverty. Part of the reason for this could be that these measures are based on aggregated data and do not consider individual circumstances – for example, people who retire early and live off their accumulated wealth may be grouped into the low‐income category. On the other hand, some people may have incurred large debts in the past which still cause financial hardship, but they wouldn't be counted as poor if they had incomes above the official poverty line. Housing costs can also vary greatly within a population and may have a much higher impact on low‐income people. In OECD countries, 36% of low‐income tenants are considered to be over‐burdened by housing costs (OECD, 2022). Income‐based measures wouldn't discriminate between low‐income households that are struggling in this way and those that aren't (e.g., who live in a house without a mortgage). As pointed out by Meyer and Sullivan (Meyer & Sullivan, 2012, p. 116), “income‐based measures […] will not capture differences over time or across households in wealth accumulation, ownership of durable goods such as houses and cars, or access to credit.” As such, this review examines studies of GBI interventions that use alternative measures, as described above, to assess their effectiveness for poverty reduction.

### 3.4 Why it is important to do this review

As far as we are aware, this systematic review is the first to focus only on poverty‐related outcomes and to quantitatively evaluate the effectiveness of various forms of GBI for reducing poverty in developed high‐income countries, using food insecurity level, consumption, material deprivation and multidimensional poverty indicators as outcomes of interest. Although other reviews have included outcomes related to various dimensions of poverty, this review attempts to synthesize findings related to all relevant material, social, and psychological outcomes according to current multidimensional conceptualizations of poverty.

We found the following reviews that included GBI‐like interventions in high‐income countries (including one overview of reviews), which differ from ours in scope and objectives:

- Hasdell (2020) conducted a synthesis of reviews, published between 2011 and 2020, of interventions globally that included at least two features of UBI. Three reviews for low‐ and middle‐income countries were included that reported on food insecurity or material deprivation.
- Chrisp et al. (2022) conducted a rapid evidence review of income assistance experiments in OECD countries that included at least one of the following features: universality (within some population), unconditionality or non‐withdrawability (with other income). The objective was to provide an evidence base to inform policymakers regarding knowledge gaps and suggest guidance for future experiments.
- Gentilini et al. (2020) produced a guide published by the World Bank that examined interventions similar to UBI globally and included one study in Sub‐Saharan Africa that reported on food security. Effects on poverty were assessed using two measures which are based on income alone: the poverty headcount and the squared poverty gap.
- Gibson et al. (2020) conducted a scoping review of interventions similar to basic income in upper‐middle‐income and high‐income countries. This review examined health outcomes. Two included quantitative studies reported increased birthweight, one reported improved nutrition. One qualitative study was included that reported improved food security.
- Günther (2020) reviewed 60 articles as part of a Master's thesis on basic income schemes and experiments globally to evaluate income and employment elasticities.
- Gupta et al. (2021) conducted a review of basic income experiments globally and examined the effect of mitigating income poverty on mental health.
- Pinto et al. (2021) conducted a systematic review that identified 86 articles on 10 basic income interventions implemented globally to examine the various methods used to evaluate the effectiveness of the interventions.
- Somers et al. (2021) reviewed unconditional and conditional income support experiments and simulation studies from around the world, with the aim of synthesizing the evidence on intended and unintended micro‐ and macroeconomic effects.
- Wilson and McDaid (2021) conducted a review of qualitative and quantitative studies of basic income interventions in high income countries to examine their effects on mental health.
- Yang et al. (2021) reviewed 152 pieces of literature on basic income theories and empirical cases (15 studies globally) to analyze the relationship between conceptual definitions of basic income and how interventions have been implemented.

#### 3.4.1 Policy relevance

Although GBI as it is thought of today was first proposed by Thomas Paine in the 18th century, there has been a resurgence of support for GBI in recent decades by advocates in various fields: philosophy, economics, social policy, high‐tech, and notably, from opposing points on the political spectrum (Alston, 2017). However, a major obstacle to constructive policy debates on GBI is that the theoretical conceptualizations of basic income – usually the universal variety – do not quite align with the ways in which GBI programs, pilots and experiments have been implemented in practice (Gentilini, 2020; Yang, 2021). The disconnect between theoretical conceptualizations and the actual designs of empirical GBI interventions, as well as the heterogeneity of these designs, makes it difficult to agree on principles to guide the development of full‐scale GBI programs (Gentilini, 2020; Yang, 2021). Because empirical GBI interventions only include some features of a true UBI and often enroll participants based on having income below some threshold, there is also ambiguity between the definitions of these interventions and those of liberal welfare programs (with less stringent eligibility criteria than workfare programs). As well, the roles of various stakeholders – researchers, politicians, advocates, communities, news media – give rise to competing expectations which may result in misperceptions of the findings of GBI studies (Merrill, 2022). For these reasons, we developed a framework as part of this review to facilitate the evaluation and comparison of various types of GBI interventions, so that empirical evidence can be more objectively assessed and synthesized, and thus be more useful for policy discussions.

The inclusion of alternative and novel poverty measures in this review would also be relevant to public and social policy, particularly with respect to health and healthcare. The association between poverty and poor physical and mental health has been well documented (Boozary & Shojania, 2018; Gundersen & Ziliak, 2015; McLeod & Veall, 2006; Seligman & Schillinger, 2010). Income, however, was found to be a weak determinant of health in a large study by the United States Department of Agriculture, which reported that income was associated with three of ten chronic diseases, while food insecurity was associated with all ten (Gregory & Coleman‐Jensen, 2017). Thus, if policymakers rely on OPMs based on income, with the assumption that poverty is being measured accurately, vulnerable populations not identified by the poverty measure may be overlooked (Cutillo, 2020).

A review of GBI interventions is relevant to discourses on public and social policy since the main goals of GBI are to reduce poverty and societal inequity. Moreover, GBI may benefit a specific population which does not qualify for regular social assistance benefits: the “working poor” (Caputo, 2007; Koebel & Pohler, 2019; Riches & Tarasuk, 2014). While social assistance eligibility has become more restrictive in recent decades, real income from employment has remained stagnant. According to the Economic Policy Institute (EPI, 2021), productivity and workers' wages increased at almost the same rate in the United States from the 1940s until the early 1980s. Since then, while productivity has continued to grow at the same pace, increasing by 62% between 1980 and 2020, wages have only increased by 17.5% in these four decades. Over the same time, the income gap between the rich and the poor has grown much wider: household income for the lowest quintile, adjusted for inflation, remained essentially unchanged between 1973 and 2015, and increased by about 20% for median‐income families, whereas it increased by 60% for the wealthiest 5% (Stone, 2020). This suggests that most of the wealth generated by the increased productivity during recent decades has gone to those who are well‐off financially. The reasons for this divergence include labor laws that favor corporations over unions, decreasing tax rates for the wealthy, and small increases in the minimum wage which have not kept pace with inflation (EPI, 2021). These factors, combined with the outsourcing of jobs to developing countries, and workfare programs that place more people into precarious low‐paying jobs, have resulted in increasing numbers of the “working poor.”

## 4 OBJECTIVES

This systematic review aimed to appraise and synthesize the available quantitative evidence on GBI interventions in high‐income countries, for the purpose of comparing the relative effectiveness of specific forms of GBI for alleviating poverty, compared to existing social assistance programs. As such, we sought to answer the following research questions:

- What are the effects of various forms of a GBI on poverty‐related outcomes in high‐income countries?
- Is there sufficient evidence available to determine a minimum amount of GBI to effect significant reductions in poverty (indicated by reported effects on poverty‐related outcomes)?
- Does GBI affect subgroups within the population differently (by age, ability, education, gender, ethnicity, etc.)?
- How do estimated effect sizes vary with the type of poverty measure used (income based, consumption based, multidimensional measures, and food insecurity level)?
- What is the relationship between the various measures of poverty (i.e., which ones predict similar effects across different types of interventions)?

## 5 METHODS

This review was conducted according to the methods described in the protocol (Rizvi, 2022), with some exceptions. Because of the atypical nature of GBI empirical research, as described below, it was necessary to modify some of the planned methods so that they were applicable to this specific area of research. The reasons for each modification are described in the relevant sections below.

Because we found that the types of interventions used in the included studies differed from theoretical concepts of a GBI, and because definitions and measures of poverty are still evolving, it was necessary to use an exploratory, inductive (“blank slate”) and iterative approach in conducting this review. As such, we considered all available, relevant empirical evidence for inclusion and did not rely on an *a priori* framework to interpret the findings and to draw conclusions.

To help improve the completeness and transparency of the review, we followed the Methodological Expectations of Campbell Collaboration Intervention Reviews (MECCIR) guidelines (Methods Group, 2019a, 2019b).

We stated in the protocol that we would also follow the PRISMA‐Equity reporting guideline (Welch, 2012) because of the association between poverty and societal inequity. However, we realized that this guidance was more suited to reviews of epidemiological studies that reported data on health equity, so we decided to follow the standard PRISMA guidance (Moher, 2009), which is integrated into the MECCIR reporting guideline.

We also used the AMSTAR 2 critical appraisal instrument (Shea, 2017), intended to assist policymakers in assessing the quality of systematic reviews, to ensure that this review addressed each of the 16 items in the AMSTAR 2 checklist.

### 5.1 Criteria for considering studies for this review

#### 5.1.1 Types of studies

The review included primary studies that collected and analyzed quantitative data on poverty‐related effects of GBI interventions. Any longitudinal study was eligible, including, but not limited to, the following designs:

- Randomized controlled trial (RCT)
- Cluster randomized controlled trial (cRCT)
- Controlled before and after (CBA)
- Regression discontinuity design (RDD)
- Interrupted time series (ITS) with at least three time points before, three time points after and a time‐series analysis
- Cohort (prospective or retrospective, including repeated cross‐sectional), with or without a control group, and with at least two repeated outcome measures

Cross‐sectional studies (using data from a single time point) were excluded as they do not examine change over time in a particular cohort. We also excluded predictive modeling and simulation studies, qualitative studies (e.g., case reports, narrative reports of interviews or focus groups) as well as any secondary sources. These sources include reviews and overviews of studies, books, news and magazine articles, editorials, opinion pieces, and blogs.

#### 5.1.2 Types of settings

We included studies from any setting in developed high‐income countries, according to the classification of the United Nations Department of Economic and Social Affairs (UN DESA, 2022). Some countries that fall under the high‐income country category of the World Bank (e.g., Chile, Oman, Saudi Arabia) are classified by the International Monetary Fund (IMF, 2022) and UN DESA as emerging market economies, developing economies and/or developing countries. Because these terms are commonly used to refer to low‐ or middle‐income countries in research articles, reports, and policy discussions, we only included studies from high‐income countries that are classified by UN DESA as developed countries, to avoid potential confusion.

We used the 2022 UN DESA country classifications regardless of the date of the study.

#### 5.1.3 Types of participants

We included studies involving any group of people in high‐income countries (as defined above). Children were included since some studies examine outcomes for the children of parents or guardians who receive GBI benefits.

#### 5.1.4 Types of interventions

We included any cash transfer programs for adults (18+ years old) in high‐income countries that met our four criteria for GBI interventions: (1) regular payment intervals, (2) paid in cash (not in‐kind), (3) a guaranteed minimum amount received unconditionally, and (4) fixed or predictable amounts.

Refundable tax credits (also called payable tax credits) were excluded because they are either conditional (e.g., on being employed, enrolled in a training program) or they are intended to offset specific expenses (e.g., health insurance premiums, the costs of raising children or caregiving for adults). Also, governments may increase or decrease the amount of these benefits over time. As such, refundable tax credits do not match our definition of providing a fixed base which can be supplemented by other income.

GBI benefits could be paid on an individual or household basis. The interventions could be administered by governments (usually as pilot projects) or by nongovernmental or civil society organizations for research purposes. In studies that included control groups, usual care would be in the form of conventional government assistance programs for participants who were eligible to receive them and no government assistance for those who were not.

#### 5.1.5 Types of outcome measures

This review examined two primary outcomes. The first was food insecurity level, which is typically assessed using survey‐based, self‐reported and validated measures of food insecurity. The survey responses are quantified using scoring rubrics, which vary among high‐income countries. Examples of measures include the UN FAO Food Insecurity Experience Scale (FIES) (FAO, 2018), used in Europe, and the Household Food Security Survey Module (HFSSM) (USDA, 2022), used in the United States and Canada (the same survey but with slightly different thresholds for levels of food insecurity).

The second primary outcome was poverty level assessed using measures that are intended to determine poverty thresholds or to index poverty levels across national or international contexts. Examples of eligible instruments included:

- National or international measures such as the United States' OPM and Supplemental Poverty Measure (SPM) (IRP, 2017), Canada's MBM (Statistics Canada, 2022), OECD relative poverty measure, and the poverty gap index (OECD, 2022)
- Consumption‐based indicators such as the Household Budget Survey (HBS) (Eurostat, 2023), and Consumer Expenditure (CE) Survey (US Census Bureau, 2021)
- Measures of deprivation such as the European Union's Material Deprivation (MD) Index (Eurostat, 2012), and other measures of ability to cover basic needs.

For the secondary outcomes, all measures listed below were eligible for inclusion. Some outcomes such as weight and height measures, used to determine body mass index (BMI), are measured using instruments or self‐reporting, while other outcomes such as self‐reported health status are measured using validated scales (e.g., the SF‐12 Survey for physical and mental health; RAND, 2018) or subjective ratings (e.g., 0–10 scale). Some secondary outcomes can be individual components of poverty indicators (e.g., food expenditure would be a component of a consumption measure).

We did not exclude studies based on pre‐specified outcomes because we expected to find other poverty‐related outcomes reported in studies, which were important to include in this review.

##### Primary outcomes

- Food insecurity level (using survey‐based, validated measures, as described above)
- Poverty level assessed using instruments intended or designed to measure poverty: income‐based OPMs; novel national or international measures of material hardship/deprivation or consumption of goods and services; multidimensional measures of physical, social and/or psychological wellbeing.

##### Secondary outcomes

- Food expenditure
- Self‐reported physical health
- Self‐reported mental health
- Body mass index (BMI)
- BMI for age
- Mid‐upper arm circumference (MUAC)
- Birth weight of children
- Cognitive development, literacy, and numeracy of children
- School/training program enrollment (children and adults)
- Individual/household earnings

#### 5.1.6 Duration of follow‐up

No restrictions were placed on the duration of follow‐ups.

#### 5.1.7 Language

We limited the included studies to those that were published in English. Although our team included multiple reviewers who were fluent in French, Spanish, and other languages, it was not feasible for pairs of reviewers who spoke the same non‐English language to commit to all the stages of the review that had to be conducted in duplicate (i.e., screening, data extraction, and risk of bias assessments).

### 5.2 Search methods for identification of studies

This review focused on studies that investigated GBI programs, pilots and experiments in developed high‐income countries. The search strategy used for this review builds on those used in previous reviews on GBI (Gibson, 2020; Pinto, 2021). Searches using both keywords and database‐specific controlled vocabulary were conducted in relevant databases, and complementary searches were done to identify additional studies as well as pertinent gray literature.

#### 5.2.1 Electronic searches

Searches were conducted in subject‐specific and multidisciplinary databases to identify relevant published studies to include in this review. Searches were executed by PRL in the following databases (in alphabetical order): APA PsycInfo (Ovid), Academic Search Complete (EBSCOhost), Business Source Complete (EBSCOhost), Cochrane CENTRAL (Ovid), CINAHL (EBSCOhost), EconLit (EBSCOhost), Embase (Ovid), Global Health (EBSCOhost), International Bibliography of the Social Sciences (ProQuest), International Political Science Abstracts (EBSCOhost), MEDLINE (Ovid), PAIS Index (ProQuest), Sociological Abstracts (including Social Services Abstracts, ProQuest), Web of Science (Science Citation Index Expanded, Social Sciences Citation Index, Arts & Humanities Citation Index, Emerging Sources Citation Index; Clarivate), Worldwide Political Science Abstracts (ProQuest).

Database limits were not used, and no restrictions related to languages or dates were imposed when searching the above resources. Some databases indexing various publication types were limited, to retrieve only scholarly journal articles, theses, or reports.

An initial, sensitive search strategy was developed for MEDLINE (Ovid). Given the scope of this review, the research librarian (PRL) and principal investigator (AR) determined that searching broadly for studies related to GBI would suffice and that no additional concepts would be included and combined. To assess its effectiveness, the strategy was peer‐reviewed by another research librarian following the Peer Review of Electronic Search Strategies (PRESS) guideline for systematic reviews (McGowan, 2016). This search strategy was then translated for the other databases using pertinent subject headings, where applicable, as well as appropriate search syntax. The complete search strategies are available in Supporting Information: Appendix 1. Searches were executed on May 16 and 17, 2022.

In addition to using the above resources, searches were done in the Cochrane Database of Systematic Reviews (Ovid), the Campbell Systematic Reviews journal (Wiley), the Social Systems Evidence database (McMaster University), and Epistemonikos (via the Cochrane Library) to identify relevant review articles.

Included studies were added to Zotero, which integrates notifications from Retraction Watch (https://retractionwatch.com/), to determine if any of them have been retracted. The Retraction Watch website was also used to search for each included study to ensure that none were retracted. Both steps were performed in October 2022.

#### 5.2.2 Gray literature

Various approaches were used to identify relevant gray literature.

To find conference proceedings, both the Science and the Social Sciences & Humanities editions of the Conference Proceedings Citation Index were searched (at the same time as other indexes on the Web of Science). In addition, reviewers consulted specific conference websites for the BIEN Congress (https://basicincome.org/congress-papers/) and for the Annual BIG Conference (https://usbig.net/2022congress/) to browse proceedings and presentations made from 2017 through 2022.

To identify relevant graduate research, a search was conducted in ProQuest Theses & Dissertations Global (ProQuest). Additional theses were found through other electronic databases mentioned above that also index graduate works.

To identify relevant government documents and other types of gray literature such as white papers and preprints, we searched the websites and catalogs of the following organizations:

- United Nations (via ODS; https://documents.un.org/prod/ods.nsf/home.xsp, and via the UN Digital Library; https://digitallibrary.un.org/)
- World Bank (via the Open Knowledge Repository; https://openknowledge.worldbank.org/, and via its eLibrary; https://elibrary.worldbank.org/)
- World Health Organization (https://www.who.int/publications)
- Social Science Research Network (https://www.ssrn.com/)
- National Bureau of Economic Research (https://www.nber.org/)
- Research Papers in Economics (https://repec.org/)
- Institute of Labor Economics (https://www.iza.org/)
- OECD (via its iLibrary [subscription access])

Targeted, specific searches were also conducted of government websites of Group of Seven (G7) high income countries:

- Canada; https://publications.gc.ca/site/eng/home.html
- France; no search option for English
- Germany; https://www.bundesregierung.de/breg-en/service/information-material-issued-by-the-federal-government,
- Italy; no search option for English
- Japan; https://www.japan.go.jp/publications/index.html
- United Kingdom; https://www.gov.uk/official-documents
- United States; https://www.govinfo.gov/

To locate gray literature as described above, members of the review team searched sites and tools throughout September 2022 using the following phrases: basic income, unconditional cash transfer, unconditional cash transfers, negative income tax and guaranteed annual income.

#### 5.2.3 Other resources

In addition to searching for gray literature, other means of identifying studies were used from September to December 2022, as described below.

Reference lists from relevant knowledge syntheses (systematic and nonsystematic reviews) as well as those from included primary studies were examined to see if other studies should be considered. Citation searching of the included articles was also conducted using Google Scholar (https://scholar.google.com/).

Once title and abstract screening was completed, journal titles of references eligible for full‐text review were analyzed to select the five journals that appear most frequently. These journals (American Economic Review, American Journal of Sociology, Australian Journal of Social Issues, Basic Income Studies, Journal of Human Resources) were then hand searched in December 2022 by looking specifically at each issue's tables of contents for 2017 through 2022.

Internet searches were continued by the principal investigator (AR) until December 2022 (while the screening and data extraction stages were completed) to identify any missed or newly published articles that would be eligible to include.

### 5.3 Data collection and analysis

#### 5.3.1 Description of methods used in primary research

GBI interventions (programs, experiments, and pilot studies) have typically been carried out within selected geographic regions with participants whose income falls below a certain threshold amount. Some interventions employed a saturation approach where every eligible person in the community who enrolls received the benefit, so that community‐level effects could be examined. Programs which would meet our criteria for GBI could also target specific populations. Although the types of outcomes were numerous, data were usually collected using surveys completed by participants, while for other outcomes, data were obtained from administrative databases such as school board records or police records.

Most basic income experiments that matched our GBI criteria were conducted as RCTs with intervention and control groups, while others were of a quasi‐experimental (observational) nature, some using statistical controls such as propensity score matching to reduce bias.

#### 5.3.2 Selection of studies

All stages of reference screening were conducted with the use of Covidence, an online tool designed to streamline certain stages of review projects (https://www.covidence.org/). A summary of the inclusion and exclusion criteria (see Supporting Information: Appendix 2) was posted on Covidence for reference during the screening process. The selection of studies began with title and abstract screening, performed independently by two reviewers. In case of disagreement, the decision on including the reference was made by the principal investigator. The same process was used at the full‐text screening stage to determine the eligibility of the references that were retained after title and abstract screening. The reasons for excluding references at this stage were recorded and summarized in a PRISMA flow diagram.

Both screening phases were subject to a pilot phase to ensure that the inclusion and exclusion criteria were clear and applied consistently. Reviewers provided feedback during both pilot stages regarding the clarity of the inclusion and exclusion criteria, and we refined the wording based on the feedback if two or more reviewers agreed with the suggestion.

The title and abstract screening procedure was piloted by six reviewers (MBD, MKS, MMK, MYY, OD, OM) on 25 randomly selected references. After the pilot phase, the title and abstract screening was completed by nine reviewers (AA‐Z, AKH, AR, MBD, MKS, MMK, MYY, OD, OM).

The full‐text pilot was conducted by five reviewers (AA‐Z, AR, KH, MBD, MMK) on 15 randomly selected references (from those remaining after title and abstract screening). Subsequently, the full‐text screening was completed by six reviewers (AA‐Z, AKH, AR, MMK, PRL, OD).

#### 5.3.3 Exclusion of studies

We excluded studies at three stages: title and abstract screening, full‐text screening, and data extraction.

We did not screen references by outcome because we did not know in advance exactly which poverty‐related outcomes would be reported in the included studies. As well, some studies might have examined eligible outcomes as secondary outcomes, so we didn't want those studies to be excluded at the preliminary title/abstract screening stage.

After data extraction was completed and all the reported outcomes were compiled, we identified outcomes that, while generally associated with poverty, were ambiguous in their effect on poverty. That is, for these outcomes, it was not clear whether the experience of poverty was ameliorated or exacerbated when a change in the outcome was reported. Studies were excluded if they only reported outcomes of this type. The outcomes that were retained were those for which beneficial or adverse impacts on the study participants were self‐evident (e.g., debt, health status, life satisfaction, agency).

Outcomes that could be poverty‐related but were ambiguous as to the direction of poverty reduction within the study included: marital stability or dissolution (separated spouses received more in combined benefits, but combined individual expenses were higher also), awareness of social services (no longer relevant to current programs), “fertility” (larger households received larger benefits), and moving/migration.

We excluded employment‐related outcomes such as job searching and labor market participation because having a paid job involves work‐related expenses (transportation, clothing, purchased lunches, paid daycare, etc.). The studies that examined such outcomes were based on interventions which entailed reductions in benefit payments as employment income increased, so it was not clear if participants were financially in a better or worse position if the amount of paid work increased.

We also excluded studies at the data extraction stage if they were deemed by two reviewers to include some aspect that did not match our eligibility criteria.

Some outcomes were also excluded at the outcome classification stage (described below) because some potentially relevant outcomes were extracted into categories provided in the extraction form (e.g., “prescription of painkillers” added under “physical health”) but which were not clear as to the direction of the effect on poverty (i.e., an increase in prescriptions could indicate poorer health or more affordability of time or money).

Lastly, we excluded studies on experiments conducted or completed after February 2020 because of the potentially confounding impacts of the COVID‐19 pandemic.

#### 5.3.4 Data extraction and management

Data was extracted by two reviewers working independently, using an extraction form in Excel (Microsoft, 2022), based on the coding template in Supporting Information: Appendix 3. The form was piloted with five studies with diverse designs and outcomes, to check if more questions or categories were required in the form to capture all relevant information on the population, setting, study design, intervention, data collection and analysis, and outcomes. Five reviewers (AA‐Z, AR, MBD, MKS, MMK) performed the pilot extractions on two studies each, resulting in five pairs of completed extraction forms. The revised extraction form was then used for the rest of the included articles, five of which were done by the reviewers that did their extractions for the pilot. The extractions for all the included studies were completed by 20 reviewers (AA‐Z, AC, AR, CMA, EG, HNE, HS, JMA, LG, LI, MBD, MG, MKS, MMK, NE, OD, OM, SIH, SN, SR) working in pairs on each study. Each pair of reviewers exchanged their forms by email, and discrepancies were discussed and reconciled to create a final extraction file for each study.

For multi‐arm studies, we included only the intervention and control groups that met our inclusion criteria. We note in the “Characteristics of included studies” table (Section 6.1.4 below) where ineligible arms were excluded.

We did not extract statistical results data into the extraction forms because effect estimates were often presented in complex ways, with various subgroup results reported for up to eight intervention arms, sometimes in several tables, for each outcome. It was more efficient to note the relevant table numbers for each outcome in the extraction form (done by two reviewers independently), and then refer to the original tables during the analysis stage of the review. This also reduced the likelihood of transcription errors and losing discrete or disaggregated data presented in the original tables. Where meta‐analyses were possible, the data were entered into the RevMan analysis tool by AR. Where meta‐analyses were not possible, the data were entered into the results tables created in RevMan by AR for each outcome category. To ensure the accuracy of the entered data, the results in the forest plots and in the tables of findings of the individual studies were compared by AR to the data in the study reports.

##### Classification of outcomes

Once the data extraction was completed, all the extracted outcomes were organized into categories and subcategories by AR to facilitate the analysis of the study findings. This was done through an inductive and iterative approach using an Excel worksheet to arrange the outcomes in one column, along with (in adjacent columns): the article reference, experiment name, outcome definition or measure used, as well as possible terms (or labels) to use for the outcome categories and subcategories. Visual inspection and the Excel “sort” function were then used to group similar outcomes. The category and subcategory names were subsequently refined by grouping (e.g., merging “community engagement” into “social engagement”) and revising the terminology (e.g., distinguishing “social perceptions” from “social engagement”). Some outcomes were extracted from the articles using an “other” category in the extraction form. For these outcomes, category and subcategory names were also developed using the process described above.

#### 5.3.5 Assessment of risk of bias in included studies

We used an adapted version of the risk of bias tool described by Sharma Waddington and Cairncross (2021), which builds on previously developed tools (Eldridge, 2016; Higgins, 2016; Hombrados & Waddington, 2012; Jimenez, 2018; Sterne, 2016; Waddington, 2017), and combines scoring criteria for randomized and non‐randomized designs so that the quality of studies using either design can be compared.

We assessed the risk of bias in the included studies across the following seven domains:

- Confounding (nonexperimental differences between intervention and groups, or imbalances not controlled in analyses)
- Selection bias (study sample at baseline is not representative of the target population)
- Attrition bias (entire sample or study group at endline is different in relevant characteristics from baseline)
- Motivation bias (participants and/or study personnel are unblinded; participants' responses are affected by their involvement in or knowledge of the experiment)
- Implementation bias (intervention is not received by all participants or not received as intended)
- Measurement error (use of subjective, inaccurate, or inappropriate measures)
- Analysis and reporting bias (inadequate analysis methods, selective reporting, or no pre‐analysis plan)

Sharma Waddington and Cairncross also include an eighth domain (unit of analysis error), which we did not incorporate into our risk of bias assessments since we addressed this type of bias separately, as described in the “Unit of analysis issues” section below. We used the term “implementation bias,” whereas the term in the original cited tool is “performance bias.” Lastly, for the “selection bias” domain, we added “truncation” as a potential source of bias since many studies used an income threshold for participant enrollment (which can lead to skewed data), and we removed “immortal time” as an example of data censoring, since none of the included studies in our review examined mortality outcomes.

Each study's risk of bias was assessed independently by two reviewers from among AA, AA‐Z, AC, AR, BJS, EK, JJ, LI, MBD. MMK, MYY, NE, OM, SH, and SR. Ratings of “low risk,” “some concerns” or “high risk” were assigned for each of seven domains. The ratings for each study were entered into a form (Excel spreadsheet) created by AR, which contained instructions based on those in the original tool (H. Sharma Waddington, personal communication, December 10, 2021). The form also included dropdown lists to select ratings for each domain, as well as spaces to provide justifications for each rating. We resolved discrepancies by consensus, and in case of disagreement, the higher risk of bias rating of the two reviewers (i.e., the more cautious rating) was used for the reconciled assessment. To calculate an overall risk of bias score, we converted the ratings to numerical values (low risk = 1, some concerns = 2, high risk = 3) and then summed up the values to get an overall score between 7 (low risk in every domain) and 21 (high risk in every domain). This approach gives equal weight to each domain and assumes that the risk of bias is additive, such that if a high risk of bias is assessed for three domains, for example, then the overall risk would be much higher than if only one domain is assessed to have a high risk of bias. There are limitations to using an overall score in systematic reviews, especially if studies are weighed by their quality in meta‐analysis (Jüni, 2001); however, we did not use the overall scores for the purpose of weighting, but rather as a way to summarize and compare the risk of bias across the included studies.

#### 5.3.6 Measures of treatment effect

We conducted meta‐analyses when comparisons across studies were feasible, if point estimates and confidence intervals were provided or could be calculated using the reported data. In most cases, pooled estimates were not feasible due to heterogeneity in outcomes, populations, and interventions. In these cases, we performed meta‐analyses (without pooled results) to conduct comparisons and depict them visually as forest plots.

Where studies used different scales to measure the same outcome, we ensured that the direction of interpretation was consistent by checking whether a higher or lower score indicated a favorable effect. If the meaning of the score was unclear, we referred to the study authors' conclusions to ascertain how to interpret the reported result (and whether to reverse the direction if the result was used in a meta‐analysis).

Where sufficient statistical information was provided in each included study, we calculated Cohen's *d* as the standardized mean difference (SMD) to estimate true effect sizes, along with 95% confidence intervals, to compare effect sizes where two or more studies report on the same outcome but measure it in different ways. The magnitude of the SMD or *d* indicates the number of standard deviations (SDs) between the means that are being compared (e.g., for the intervention and control groups).

The calculation of *d* was performed using the Campbell Collaboration effect size calculator (https://www.campbellcollaboration.org/escalc/). This tool provides separate interfaces for inputting various types of statistics to calculate the SMD, including *t* values, *t*‐test *p* values, means with SDs or standard errors (SEs), binary proportions, frequency distributions (for categorical variables), and regression coefficients (standardized and unstandardized).

If studies reported only means (or mean differences) without SDs, *p* values, *t* values or confidence intervals, or only provided graphical representations of the effect estimates (e.g., box‐and‐whisker plots), we contacted the corresponding authors of those studies (if published within the last ten years) to ask if these statistics could be provided.

We investigated whether it was necessary to use Hedges' *g* instead of Cohen's *d* as the standardized mean difference because Hedges' method uses a correction factor in calculating the SMD to reduce bias due to small sample sizes, which can exaggerate the effect size (Borenstein & Hedges, 2019).

We tested the correction factor [J(*df*) = 1 − (3/(4*df* − 1))] on the two included studies with the smallest sample sizes (*N* = 138 in Mallar, 1977, *N* = 266 in McDonald, 1979) and found that the correction was less than 1% in both cases. Since this value was negligible and the other included studies had larger sample sizes, we chose to use the more common *d* as the SMD for estimating true effect sizes.

We used a significance level of 5% (i.e., *p* = 0.05) as a threshold to decide whether there was evidence of an effect for each outcome. Although it is preferable to examine effect estimates along with their confidence intervals instead of relying on *p* values (Schünemann, 2023), this was not possible for most of the included outcomes because the confidence intervals were not reported by the study authors or there was not enough statistical information reported to calculate them. Conversely, *p* values were either reported in all the studies or we were able to calculate them (e.g., using reported *t* values). Therefore, using *p* values provided a consistent way of interpreting the findings, whether we were able to calculate a pooled result across studies or where a particular outcome was only reported in a single study.

We recognize that using a *p* value threshold is somewhat arbitrary, based on the need to “draw the line somewhere” in terms of certainty. We also recognize that a *p* value greater than 0.05 indicates a lack of evidence (e.g., due to an inadequate sample size) and not evidence that the intervention did not affect the outcome (Schünemann, 2023). This is especially relevant to the results of the included studies because the determination of *p* values (as well as confidence intervals) assumes that study samples are randomly drawn from the population (Andrade, 2019), and that was not the case with the GBI experiments. Therefore, additional caution was warranted when interpreting the findings from individual studies; thus, results with *p* values higher than 0.05 were interpreted as “no evidence of effect.”

#### 5.3.7 Unit of analysis issues

Unit of analysis error can occur when the intervention is provided at a cluster level (e.g., school, clinic, or household) and the analysis is conducted at the individual participant level (e.g., student, patient, or husband/wife). The responses of participants from the same cluster unit (e.g., spouses in a household) may be more similar to each other than to other participants in the study. Because the responses in the same cluster may not be independent, an individual‐level analysis using the total sample size may underestimate the true variance. If multiple observations per cluster were included in the study, we assessed if the researchers used an appropriate analysis approach, such as multilevel modeling, variance components analysis, or cluster‐level fixed effects, to yield more realistic confidence intervals and *p* values.

If this potential bias is not controlled for in the study, a corrected standard error, SE′, can be calculated as:

SE′=SE×SQRT(1+(m−1)×ICC)

where SE is the uncorrected SE, *m* is the number of observations per cluster, and ICC is the intra‐cluster correlation coefficient (Waddington, 2012).

If there is insufficient data to calculate the ICC, the value can be estimated based on the reported ICC in other similar studies on similar outcomes (Hedges, 2007). However, we were unable to find studies that reported consistent ICCs to draw on, especially with household‐level interventions, so we were unable to calculate the corrected SE. It is unclear whether this is a problem since most of the included studies in this review only used observations from one person per household (usually the recipient of the GBI benefit). In studies where more than one spouse or child per household was observed, the average number of individuals per household was approximately 1.6 because some households had single heads (i.e., only one participant per “cluster”) and observations on children were usually for a limited age range, resulting in one or two children typically sampled from the same household. Using the formula above and inputting a “medium” ICC value of 0.5, the correction factor for the SE would be 14% (i.e., the square root of 1 + [(1.6 − 1) × 0.5] is 1.14). Therefore, to account for possible unit of analysis error, we treated effect estimates with caution if the confidence interval of the reported or calculated SMD came close to zero.

We had also planned to calculate the SMD for cluster‐design studies using a Shiny app developed by Taylor and colleagues (2022) (https://airshinyapps.shinyapps.io/es_2lvl_clust_adj/), but this tool also requires inputting an ICC value, which we did not have. To address this problem, we did not pool the results of studies of individual‐level interventions with those of household‐level interventions where observations were made on more than one member.

#### 5.3.8 Criteria for determination of independent findings

Although we expected to find multiple reports of each GBI study, only two of the included articles reported on the same study. In this case, we used the data from the newer article for outcomes that were reported in both. For outcomes which were only reported in the older article, we extracted the relevant data from that one. All the other included articles reported on individual studies.

While some of the included studies drew on one common data set, we found that the datasets from GBI experiments were in effect databases or repositories containing data on a variety of outcomes in different fields of study (economic, epidemiological, social, etc.). The authors of the included studies drew on this data in a way that is similar to researchers using administrative data in their studies. As such, most of the included studies relied on a different *subset of* data.

In one case, there was an overlap in the outcomes reported in two studies on the same experiment; however, only one of the studies provided disaggregated results by intervention arm. Since some of the arms did not meet our inclusion criteria, we only used the results for eligible arms from the more comprehensive report in our meta‐analysis.

#### 5.3.9 Dealing with missing data

Studies were not excluded on the basis of which data were reported. If an included article did not report statistical data necessary for meta‐analysis and the data could not be calculated reliably (e.g., using reported confidence intervals to calculate SDs), we contacted the study authors to ask for the missing data if the article was published in the last 10 years (since 2012). This period is based on requirements for researchers to store data for a minimum of three to 6 years, depending on the country, institution, or funder (Elsevier, 2022). If we could not acquire the necessary statistical information from study authors, we did not use the result in the meta‐analysis, but did include it in the narrative synthesis.

#### 5.3.10 Assessment of heterogeneity

We had planned to use the *I* ^2^ statistic, calculated using RevMan, to examine heterogeneity. However, Borenstein (2022) recently proposed that the *I* ^2^ statistic is misused as a measure of heterogeneity and does not tell us how much the true effects vary (which we want to know to see if subgroup analysis is needed to explain the variation). Borenstein argues that the extent of heterogeneity is best represented by the prediction interval, which expresses another heterogeneity measure, Tau‐squared (*τ* ^2^), in an understandable way (Deeks, 2022). However, all of these measures (*I* ^2^, *τ* ^2^, and prediction intervals) require a sufficient number of studies to provide a useful indicator (Borenstein, 2022; Deeks, 2022; Higgins, 2003). The Cochrane Handbook recommends that more than 10 studies are needed to assess heterogeneity using such methods (Deeks, 2022).

Because there was an insufficient number of studies that we could meta‐analyze for any outcome, the above statistical assessments of heterogeneity were not appropriate, and therefore we employed “a subjective examination of the variability in point estimates and the overlap in [confidence intervals]” (Guyatt, 2011, p. 1296) by examining the forest plots that were generated in RevMan for our meta‐analyses. If any two confidence intervals in the plot did not overlap or barely overlapped, we considered this as an indication of substantial heterogeneity, and investigated whether it could be explained by differences in the interventions, the populations, or other factors.

#### 5.3.11 Assessment of reporting biases

The GBI experiments covered by the included studies were all conducted by governments (federal, state/provincial, or municipal) in partnership with research organizations (private or university‐based). Although some of the articles were published in peer‐reviewed journals (while the rest were published by the research organizations), we believe publication bias is unlikely for several reasons: (1) the direct relevance of GBI experiments to many fields of interest (politics, economics, health, social studies, human rights, etc.); (2) the large scale of the experiments, conducted publicly (not in a laboratory or remote setting); and (3) publicity in the news media due to opposing views on unconditional government‐funded income support.

To determine if outcomes were selectively reported or omitted, we searched for proposals, pre‐analysis plans, and protocols, and checked those that were found to see if they specified unreported outcomes.

Selective outcome and analysis reporting were also examined as part of the risk of bias assessments described above.

#### 5.3.12 Data synthesis

##### Quantitative synthesis

Data for use in meta‐analyses was entered into RevMan by AR. Because the same outcome was measured using different scales in each study, the reported effect sizes were converted into SMDs using the Campbell effect size calculator, and then the SMDs were entered into RevMan. To decrease the likelihood of data entry errors, the reviewer used the copy and paste functions whenever possible to transfer values from the original article into the effect size calculator, and from there into the RevMan data fields.

When there was sufficient and appropriate data to conduct meta‐analyses (i.e., two or more studies with the same design reporting on the same outcome), we calculated the pooled effect size using RevMan. We conducted random‐effects meta‐analyses to account for the observed between‐study heterogeneity.

The RevMan Web version provides a calculator for combining multi‐arm interventions with a single control group, which avoids double counting of participants in the control group. (Previous versions of RevMan required dividing the number of participants in the control group by the number of eligible intervention groups in the meta‐analysis.)

##### Narrative synthesis

Due to the variation across GBI interventions, study designs, populations, and outcome measures, meta‐analyses were not possible for many of the outcomes. In these cases, we present the findings of these studies in narrative form, including calculated effect sizes where possible.

We constructed tables to classify the studies according to the type of GBI, study design, and outcomes. We also illustrate effect sizes graphically with forest plots for studies that could be grouped and compared in a meaningful way.

#### 5.3.13 Subgroup analysis and investigation of heterogeneity

There were not enough included studies to meaningfully compare the difference in effect across subgroups, so a meta‐regression to test the mean difference between the groups was not feasible.

To examine whether GBI interventions impact social, economic, and health inequities across different population subgroups, we assessed the effects of GBI on reported outcomes using the sociodemographic categories of the PROGRESS‐Plus framework. The PROGRESS acronym stands for place of residence, race/ethnicity, occupation, gender, religion, education, social capital, and socioeconomic position, while “Plus” refers to any other factors which may be associated with disadvantage, such as age, criminal record, disability, or sexual orientation (Kavanagh, 2009; O'Neill, 2014). Depending on the context of the study, a “Plus” factor may be the most relevant (O'Neill, 2014).

The original PROGRESS‐Plus framework uses the term “socioeconomic status” (SES). We use “socioeconomic position” (SEP) to denote the same concept, according to the reasoning of Krieger and colleagues (Krieger, 1997, p. 346): “‘socioeconomic status’ blurs distinctions between two different aspects of socioeconomic position: (a) actual resources, and (b) status, meaning prestige‐ or rank‐related characteristics.”

While PROGRESS‐Plus is typically used in epidemiological studies to examine sociodemographic determinants of health, we considered it to be appropriate for investigating potential inequities for other types of outcomes as well.

We presented the findings that were reported across PROGRESS‐Plus factors in tabular format, as there was too much heterogeneity in the interventions, outcomes, and study populations to conduct subgroup analyses.

#### 5.3.14 Sensitivity analysis

We verified the robustness of the meta‐analyses by comparing the quality of the studies (as determined by our risk of bias assessments) to ensure that the effect sizes were not excessively influenced by one or more low quality studies.

#### 5.3.15 Treatment of qualitative research

We did not include qualitative research in this review.

#### 5.3.16 Summary of findings and assessment of the certainty of the evidence

We present a GRADE “summary of findings” table in Section 2, which includes an assessment of the certainty of the evidence, following the method of Schünemann and colleagues (Schünemann, 2019). We explored whether separate tables could be constructed for each type of GBI intervention (e.g., subsistence‐level benefits for households, or monthly amount below €500 for individuals), but due to the diversity of outcomes across different interventions, separate tables would have essentially presented individual findings rather than a meaningful summary.

“Summary of findings” tables typically include up to seven outcomes, selected a priori, which are deemed to be the most important for the review (Schünemann, 2019). While this is feasible in epidemiological reviews (e.g., mortality would be considered more important than mobility), it was not possible for us to rank the importance of the included outcomes because they covered various aspects of life. Additionally, we didn't screen studies by outcome so that we could find other relevant poverty‐related outcomes that were reported in eligible studies, and thus we couldn't rank outcomes a priori. Therefore, we included in our summary of findings the two primary outcomes of interest, as well as five other outcomes which were reported in more than one included study.

The five secondary outcomes were selected on the basis of relevance to the present time, so at least two studies that reported the outcome had to be from the last two decades.

The GRADEpro software (https://www.gradepro.org/) was used to assign an overall level of the quality of evidence for each assessed outcome – i.e., our level of certainty that the estimate of the effect is close to the true effect. The quality of the evidence was ranked as “high,” “moderate,” “low,” or “very low” according to assessments across five categories: risk of bias, publication bias, indirectness of outcome measures, imprecision, and inconsistency of effect estimates.

The GRADE assessment was conducted by AR and verified by JP, who has previous experience with the GRADE approach.

#### 5.3.17 Subject‐specific methods used in this review

In conducting this review, we encountered methodological challenges that may be unique to the body of research addressed here. As noted above, most of the included studies drew on data from large‐scale experiments that collected data on various aspects of the participants' lives, to see how the GBI interventions impacted them. This posed a problem for the typical nomenclature of “studies” and “articles/reports” in systematic reviews, because the included articles described each of the various studies, but there were several studies on the same GBI experiment. While much research draws on population statistics in administrative databases, the data used in the included studies was obtained experimentally and by other researchers, so this introduced another level in the overall research process, as explained below.

##### Experiments, studies, and reports

The terms “experiment” and “study” usually refer to the same undertaking, conducted by the same researcher(s). In the case of most of the research included in this review, the design, recruitment, implementation, and data collection stages were conducted independently of the analysis and reporting stages. One drawback of this in conducting a systematic review is that many of the articles that reported findings provided very little, if any, information on the design and implementation methodology.

Additionally, various types of outcomes (economic, social, epidemiological, educational, etc.) were examined by different researchers, each leading to a distinct “study” of the effects of the intervention. For this reason, it was necessary to classify the research activities as either “experiment” *or* “study” to conduct a meaningful review. For example, referring to the Seattle‐Denver study would cause confusion because that would only tell us about the intervention and where it took place, whereas there were many individual studies conducted using the data collected during the implementation/intervention process. Therefore, to clarify which stage of the research we are referring to in this review, we use the following terms:

- “experiment” to refer to the intervention and to the research stages up to data collection and storage (e.g., the Seattle‐Denver experiment),
- “study” to refer to the work of the researchers who analyzed a subset of data on specific outcomes from one of the experiments,
- “article” to refer to published material that reports the findings of a particular study.

##### Classification framework for GBI experiments

We stated in the protocol (Rizvi, 2022) that we would “attempt to develop a framework or rubric to facilitate the evaluation and comparison of various types of GBI interventions, so that empirical evidence can be more objectively assessed and synthesized, and be more useful for policy discussions” (p. 6). While conducting this review, we learned that it was impossible to conduct a meaningful synthesis without first developing a framework or typology because, while “guaranteed basic income” is usually discussed as a singular concept, the empirical evidence comes from many different variations of income support interventions that all meet the criteria of GBI. Thus, it was vital to categorize the types of GBI interventions examined in the included studies before we could consider the evidence; otherwise, our findings would be almost as vague as those of a systematic review seeking to determine “the effects of drugs.”

The classification framework was developed by AR and verified by the co‐authors. To categorize the various GBI approaches used in the included studies, an inductive approach was used wherein the specific attributes of each GBI intervention were coded according to the GBI benefit structure (e.g., fixed or income‐dependent), how other income impacted the benefit amount, whether the GBI replaced or supplemented existing social programs, and whether the benefit was paid to individuals or households. The resulting classifications were then compared to theoretical conceptualizations of GBI, to identify other possible types of interventions which were not used in the included studies, but which could potentially be implemented in an experiment or as a full‐scale program.

The resulting framework (described in the section ‘Typology of GBI approaches’ below) allowed us to categorize the various GBI interventions so that the empirical evidence could be compared in a meaningful way.

The development of this typology was a process separate from the systematic review itself; however, we present the typology in the Results section because it was a product of the analysis phase of the review, based on the characteristics of interventions used in the included studies, as well as conceptualizations of GBI explored in the sections above.

## 6 RESULTS

### 6.1 Description of studies

The following sections describe the body of research that was included in this review and how it was compiled, starting with the screening of potentially relevant references and articles.

#### 6.1.1 Results of the search

**Figure 2:**
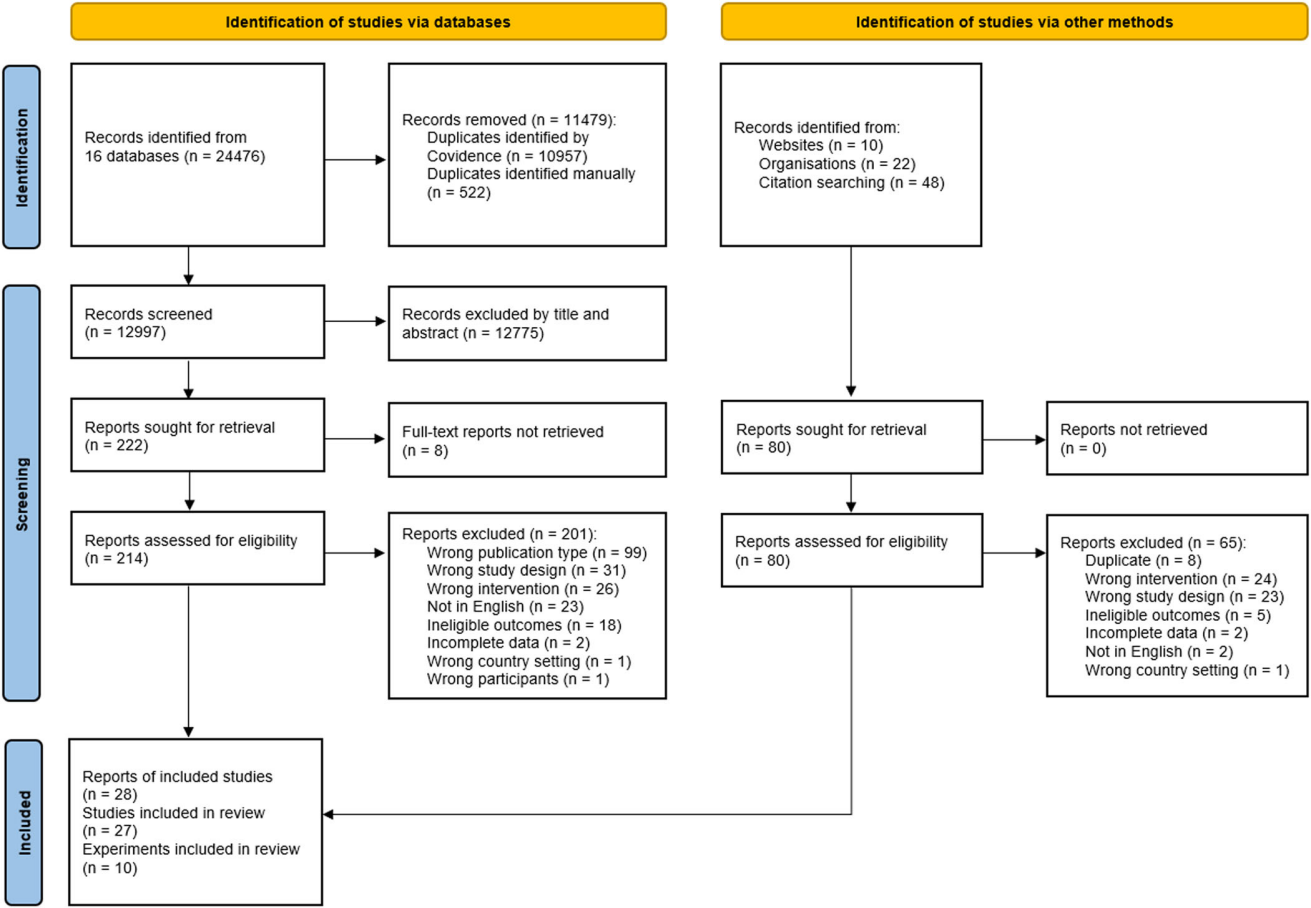
PRISMA flow diagram of the search and screening process.

The results of the search and screening process are illustrated in Figure 2. The 16 databases searched yielded 24,476 references. Eighty (80) references were identified from gray literature and through citation searching. Deduplication processes resulted in the identification and removal of 11,487 duplicate items (11,479 database results, plus 8 retrieved by other methods). The titles and abstracts of 12,997 references were reviewed in duplicate and 12,775 were excluded. The full texts of eight references could not be retrieved. Full texts for 286 references (214 from databases, and 72 found by other methods) were assessed, in duplicate, for eligibility. The hand searching was, in effect, a type of screening process because the reviewers involved were familiar with the inclusion criteria. As such, the principal investigator (AR) acted as the second screener for the records retrieved in this way.

A total of 266 full‐text articles were excluded; the most common reasons for exclusion were wrong publication type (*n* = 99), wrong study design (*n* = 54), wrong intervention (*n* = 50), and being written in a language other than English (*n* = 25). Following this two‐stage review process, we included 28 articles in this review which report on 27 studies of 10 experiments.

We found one published article for each of the included studies, with the exception of Forget (2011/2013), for which the 2013 article elaborates on some of the findings reported in the 2011 publication. Thus, we found a total of 28 articles that report on the 27 included studies.

#### 6.1.2 Included experiments

A summary of the ten included experiments is presented in Table 1. Nine experiments employed RCT designs. One of these, the Mincome experiment conducted in the Canadian province of Manitoba, included two sites: one in the city of Winnipeg (RCT site) and a “saturation” site in the town of Dauphin where every resident with income below the eligibility threshold could receive benefits, to simulate a full‐scale GBI program.

**Table 1:** Summary of Included experiments.

Experiment name	Short name	Country	Period	Included studies (primary sources)	Other sources (pre‐analysis plans, conference proceedings, final reports, books, etc.)
Barcelona B‐MINCOME Pilot	B‐Mincome	Spain	2017–2019	Bonilla (2019) Todeschini (2019)	Laín (2019)
Canada public pension study		Canada	2007–2013	McIntyre (2016a)	McIntyre (2016b)
Dutch participation income experiments		Netherlands	2017–2019	Muffels (2021)	Muffels and Gielens (2019)
Finnish basic income experiment		Finland	2017–2018	Lassander (2021) Simanainen (2021)	Kangas (2021)
Gary Income Maintenance Experiment	Gary	USA	1971–1974	Kaluzny (1979) Kehrer (1979) Maynard (1979) McDonald (1979)	Kehrer (1979a) Long (1972) Munnell (1986)
Manitoba Basic Annual Income Experiment	Mincome	Canada	1974–1978	Calnitsky (2019) Calnitsky (2021) Forget (2011/2013) Gonalons‐Pons (2021)	Hum (1979a) Hum (1979b) Simpson (2017)
New Jersey Income‐Maintenance Experiment	New Jersey	USA	1968–1972	Elesh (1977) Kerachsky (1977) Ladinsky (1977) Mallar (1977) Middleton (1977) Nicholson (1977)	Kershaw and Fair (1976a) Long (1972) Munnell (1986)
Rural Income Maintenance Experiment	RIME or Rural	USA	1970–1973	Maynard (1977) O'Connor (1979)	Bawden (1976) Long (1972) Munnell (1986)
Seattle‐Denver Income Maintenance Experiment	SIME/DIME	USA	1971–1976	Groeneveld (1979) Manheim (1979) Thoits (1979) Venti (1984)	Bell (1979) Long (1972) Munnell (1986) SRI (1983)
Stockton Economic Empowerment Demonstration	SEED	USA	2019–2020	West (2021)	Martin‐West (2019)

The tenth included experiment is a non‐randomized retrospective study of the Canada public pension, which is implemented using an NIT approach such that people of age 65 and above with no other income receive a fixed amount (approximately 80% of the national poverty line). For recipients with other income, their pension benefit is reduced by a percentage of their income from other sources (McIntyre, 2016a).

Five of the experiments were conducted between 1968 and 1978, four were conducted between 2017 and 2020, and the Canada pension study examined data from 2007 to 2013.

Five experiments took place in the US, two were in Canada, and the rest were in Barcelona (Spain), Finland, and the Netherlands.

We found 27 studies on these experiments that met our inclusion criteria. The characteristics of these studies are described in Table 3 below. We first present the typology of GBI approaches in Table 2, so that the descriptions of the interventions in the study characteristics table can be more easily understood.

**Table 2:** Types of guaranteed basic income approaches.

GBI type	Benefit structure	Integration with social assistance	Effect of earned/other income^a^	Allocation level	Other properties	Experiments
Subsistence‐level fixed amount	Fixed amount for all recipients, equal to the estimated cost of basic material needs.	Replaces basic social assistance benefits.^b^	No reduction in the benefit amount.	Household or individual	Similar to a subsistence‐level UBI, but income‐tested to provide benefits only to people with low incomes.	(None)
Supplemental fixed amount	Fixed amount for all recipients; amount below subsistence level.	Supplements social assistance benefits (and other income).	No reduction in the benefit amount.	Individual	Similar to a “partial” UBI, but income‐tested to provide support to people with low incomes.	Finland, SEED (US)
Guaranteed minimum income (GMI)^c^	Benefit amount is a “top‐up” added to earned income, so total income covers the estimated cost of basic material needs.	Replaces basic social assistance benefits.^b^	Increase in earned income results in proportional (dollar‐for‐dollar) reduction in benefit amount; fully withdrawn when earned income equals or exceeds the GMI guaranteed amount.	Household	Also called “income insurance”; similar to traditional social assistance programs, but not conditional on job seeking or other required activities. A quasi‐GBI because of the 100% withdrawal rate.	B‐Mincome (unconditional + full‐withdrawal study arm), Netherlands (5 of 8 sites: Groningen, Utrecht, Wageningen, Oss, Apeldoorn‐Epe)
Guaranteed minimum income (GMI)^c^ with partial withdrawal	Benefit amount is added to earned income (a “top‐up”), so total income covers or exceeds estimated cost of basic material needs.	Replaces basic social assistance benefits.^b^	Benefit is gradually reduced by a set percentage of additional earned income, up to a set cut‐off point.	Household	Similar to modern/novel social assistance programs (with some allowance for earned/other income), but not conditional on job seeking or participation in specific programs or activities.	B‐Mincome (unconditional + partial withdrawal study arm), Netherlands (3 of 8 sites: Deventer, Nijmegen, Tilburg)
Negative income tax (NIT)	Guaranteed, fixed benefit amount (with no other income); reduced by a percentage of earned/other income (depending on the NIT “tax” rate).	Replaces basic social assistance benefits.^b^	Benefit is gradually reduced by a percentage of additional earned income, up to a break‐even point where the benefit amount becomes zero (and earned income is well above the NIT guaranteed minimum).	Household	Similar to GMI with partial withdrawal, but the guarantee amount is not based on a calculation of the cost of basic living expenses. The guarantee amount is either arbitrary or a percentage of the official poverty line, usually 50%, 75% or 100%; various “generosity” levels have been tested in experiments.	Canada public pension, Gary (US), Mincome (Canada), New Jersey (US), Rural/RIME (US), SIME/DIME (US)

^a^ All types of GBI are subject to regular income taxes if a person's total income exceeds their individual tax liability threshold.

^b^ Does not replace programs for specific needs, such as disability benefits or healthcare coverage.

^c^ Some existing social assistance programs are also called guaranteed minimum income (GMI), but eligibility is conditional on job seeking, training, counseling, and/or other activities.

#### 6.1.3 Typology of GBI approaches

Our framework consists of five types of GBI, which are based on how the intervention would be implemented as a full‐scale program in terms of benefit receipt; that is, who would receive the GBI, how much would they receive, and how would the benefit be impacted by other income? The five types are summarized in Table 2.

The first type of GBI in Table 2 is the basic conceptual version: people with incomes below some threshold receiving a subsistence‐level, fixed amount. This approach resembles a livable‐wage (or “full”) UBI, except that the GBI version would have an income cut‐off threshold so that people with median or high incomes would not be eligible to receive benefits. Also, while UBI is usually conceptualized as an individual‐level citizens' wage, the subsistence‐level GBI could also be implemented at the household level, based on the number and ages of the members of the household. The net annual amount would be determined when income tax returns are filed, such that recipients with incomes above their tax liability threshold would have to repay some of the GBI amount. This would avoid a potential problem with the NIT approach that would occur where the NIT amount and eligibility are based on the previous year's income but where a person's current‐year income is much lower than the year before.

We did not find any experiments on this type of GBI in high‐income countries, but have included it in our framework as a conceptual and potentially operationalizable approach. In fact, there have been recent proposals for basic income pilots which could potentially be classified as the subsistence‐level, fixed‐amount type of GBI: one in England and one in Wales (the latter now underway), both providing unconditional monthly payments of £1600 to each participant (Sheils McNamee, 2023).

The second type of GBI in our framework provides a fixed amount well below a subsistence level, but which supplements all other income, including earned income and social assistance benefits. This type is similar to the “partial” UBI proposal, except that it would be provided only to people with low incomes. This approach would help to alleviate financial hardship due to insufficient social assistance benefits, as well as in‐work poverty for those with precarious, intermittent and/or low‐paying jobs. Two of the included experiments tested this approach: the Finnish basic income experiment and the Stockton Economic Empowerment Demonstration (SEED).

The third type of GBI is a GMI approach, which provides a top‐up amount to anyone whose income falls below an amount that is calculated as the cost of basic material needs. If the recipient's income rises, then the amount of the benefit is reduced dollar‐for‐dollar (i.e., a 100% withdrawal rate with additional income). This approach is similar to traditional social assistance programs, which also impose a 100% withdrawal rate, so there is little financial incentive to find low‐paying work, resulting in a poverty “trap” (Standing, 2021). This problem is more prevalent in high‐income countries where social assistance levels are more generous than in less developed countries (Konle‐Seidl, 2021).

The term GMI is also used for existing social assistance programs with a 100% withdrawal rate; the only difference with the GBI variant is that the benefits are not conditional on seeking employment, training, or other required activities. Therefore, although this variant meets our criteria of being unconditional and paid in regular, predictable amounts, we classified the GMI approach as a “quasi‐UBI” due to the austere nature of benefit withdrawal. If, for example, someone earns 90% of the GMI threshold through paid work and receives 10% as the top‐up, this amount does not meet the core definition of GBI as either providing a subsistence‐level income or a fixed base which can be supplemented with other income (Hoynes & Rothstein, 2019).

The fourth type of GBI also provides a GMI based on an estimate of living costs, but incorporates a gradual withdrawal of benefits as earned income increases. This approach is also used in novel social assistance programs in many European countries (Coady, 2021), but still in the conditional form. Unconditional GMI with partial withdrawal of benefits is similar to the fifth GBI type, NIT, where benefits are also reduced gradually with increased income; however, the guaranteed amount with NIT is not based on the estimated cost of basic needs, but is determined by the tax rate and the breakeven amount, as described above. Both GMI with partial withdrawal and NIT can be considered as providing an income base which can be supplemented with other income. The benefit amount drops below subsistence level when other income is earned, but the amount is still substantial until total income gets close to the (much larger) breakeven amount.

#### 6.1.4 Included studies

The studies that were eligible for inclusion in this review are summarized in Table 3. The studies are grouped by each of the ten experiments because the design and intervention details are features of the “experiment” level, whereas participant details and outcomes are features of the “study” level. This required a departure from the standard PICO (participants, intervention, comparator, outcomes) format for our “Characteristics of included studies” table.

**Table 3:** Characteristics of included studies.

Experiment	Intervention	Methods	Study ID (article type)	Participants^a^	Outcomes
**B‐MINCOME Pilot** Besòs area of Barcelona, Spain	7 arms/variants of GBI: 2 with full (dollar‐for‐dollar) withdrawal with additional income (GMI type), 5 with partial (25% to 35%) benefit withdrawal with additional income (GMI with partial withdrawal); guarantee amount calculated to cover basic material needs 3 arms excluded due to conditions for receipt of benefits	RCT with block randomization (based on expected benefit amount, eligibility for work, and homeownership); allocation by household. Duration: 22 months (2017–2019)	Bonilla (2019) (published report)	Low‐income families with at least one member aged 25–60 (*N* = 1320)	Life satisfaction (self‐rated) Measured at: baseline, 11/12 months, 21/22 months
			Todeschini (2019) (published report)	Low‐income families with at least one member aged 25–60 (*N* = 1383)	44 outcome variables: 20 economic, 3 educational, 4 mental/psychological, 7 physical health, 10 other (see Supporting Information: Appendix 5 for detailed list of all outcomes) Measured at: baseline, 11/12 months, 21 months
**Canadian public pension study** Canada	Public pension paid at age 65+ (a basic universal amount, plus a supplement, that is, reduced as other income increases – NIT type); maximum benefit equals 80% of poverty line	Repeated cross‐sectional design; data from the Canadian Community Health Survey (CCHS) (2007 to 2013); individual‐level data	McIntyre (2016a) (journal article)	Single low‐income (<$20,000/year) Canadians, ages 55–74 (*N* = 8019)	Food insecurity (self‐reported) measured using the Household Food Security Survey Module (HFSSM) Measured at: 2007–2008, 2009–2010, 2011–2012, and 2013
**Dutch participation income experiments** 8 cities in the Netherlands	8 arms: 3 sites (cities of Deventer, Nijmegen, Tilburg) with partial (50%) benefit withdrawal with earnings (GMI type with partial withdrawal), 5 sites (cities of Groningen, Utrecht, Wageningen, Oss, Apeldoorn‐Epe) with full withdrawal (GMI type); additional arms at each site with conditional benefits were excluded; basic benefit equal to 70% of the full‐time minimum wage	RCT; randomization of the target population before recruitment in two sites (Groningen and Deventer); individual‐level allocation Duration: 24 months (2017–2019)	Muffels (2021) (published report)	Social assistance recipients, not receiving disability benefit or retirement pension, ages vary by site, max. range 15–65 (*N* = 5230)	Financial stress and poverty, mental health, physical health, subjective well‐being, perceived capabilities, social participation, social trust; all outcomes self‐reported Measured at: baseline, 16–24 months
**Finnish basic income experiment** Finland	€560 paid monthly regardless of earned income (supplemental type of GBI)	RCT; individual‐level allocation Duration: 12 months (2017–2018)	Lassander (2021) (book chapter)	Recipients of social assistance, unemployment benefits or labor market subsidies, aged 25–58 (*N* = 1633)	Subjective financial well‐being (self‐rated) Measured at: 12 months
			Simanainen (2021) (book chapter)	Recipients of social assistance, unemployment benefits or labor market subsidies, aged 25–58 (*N* = 1633)	19 outcome variables: 13 mental/psychological, 6 physical health (see Supporting Information: Appendix 5 for detailed list of all outcomes) Measured at: 12 months
**Gary Income Maintenance Experiment** Gary, Indiana, USA	NIT with four configurations/combinations of a guarantee (approx. 77% or 100% of poverty line), and withdrawal rate of 40% or 60%	RCT; stratified by preexperimental income level; allocation by household Duration: 3 years (1971–1974)	Kaluzny (1979) (journal article)	Black families with at least one child under age 18 (*N* = 1780)	Probability of becoming a homeowner, rental expenditure (source of data not reported) Measured at: baseline, 1 year, 2 years, 3 years
			Kehrer (1979) (journal article)	Infant children from Black families (*N* = 404)	Birth weight (from government records) Measured at: birth during the experiment
			Maynard (1979) (journal article)	Children in grades 4–10 from Black families (*N* not reported; total observations over 3 years: 851 to 1517 depending on outcome)	Reading test score, academic grade point average, days absent (source of data not reported) Measured at: baseline, 1 year, 2 years, 3 years
			McDonald (1979) (journal article)	Children aged 16–18 from Black families (*N* = 266)	Post‐mandatory school enrollment (self‐reported) Measured at: baseline, 2nd school year during experiment
**Manitoba Basic Annual Income Experiment (MINCOME)** Province of Manitoba, Canada	Winnipeg (city) site – NIT with seven configurations/combinations of a guarantee (approx. 55%, 67% or 78% of poverty line) and withdrawal rate (35%, 50% or 75%): that is, 55/35, 55/50, 67/35, 67/50, 67/75, 78/50 and 78/75	RCT; stratified by family structure (one or two parents, one or both working) and by preexperimental income level; allocation by household Duration: 4 years (1974–1978)	Calnitsky (2019) (journal article)	Adults from low‐income families in Winnipeg (*N* = 926)	Reason for not working (self‐reported) Measured at: baseline, 11 survey waves over 4 years
	Dauphin, Manitoba “saturation” site – NIT with one configuration: guarantee of approx. 60% of the poverty line and withdrawal rate of 50%	Retrospective CBA study with matched controls outside Dauphin (in which all low‐income residents were eligible for the intervention) Duration: 4 years (1974–1978)	Calnitsky (2021) (journal article)	Towns in Manitoba and Saskatchewan (provinces in Canada) with populations between 5000 and 50,000; Dauphin intervention site pop. = ~12,500 (*N* = 15)	Overall crime rates, violent crime rates, property crime rates, other crime rates (from Uniform Crime Report (UCR) records) Measured at: yearly from 1972 to 1980
			Forget (2011/2013) (journal articles)	Residents of Dauphin and matched controls from other towns in Manitoba (*N* = ~50,000, based on 3 matched controls for each Dauphin resident)	Hospital separations (reported by all causes, non‐congenital mental health, accidents/injuries), low birth weight, at‐risk birth weight, small‐for‐gestational age – newborns (data from Manitoba Population Health Research Data Repository), grade 11/12 enrollment (data from Department of Education) Measured at: 32 six‐month intervals from 1970–85
	Winnipeg and Dauphin sites (as described above)	As described above for both sites	Gonalons‐Pons (2021) (journal article)	Low‐income, married couples in Manitoba (*N* = 641)	Frequency of divorce talk, temporary separation by wife, marital financial disagreement, marital Nonfinancial disagreement, wife's bargaining and decision‐making power (all self‐reported) Measured at: baseline, 2 years
**New Jersey Income‐Maintenance Experiment** 3 cities in New Jersey (Trenton, Paterson‐Passaic, and Jersey City) and 1 city in Pennsylvania (Scranton), USA	NIT with eight configurations/combinations of a guarantee (50%, 75%, 100% or 125% of the poverty line) and withdrawal rate (30%, 50% or 70%): that is, 50/30, 50/50, 75/30, 75/50, 75/70, 100/50, 100/70 and 125/50	RCT; stratified by preexperimental income level; allocation by household. Duration: 4 years (1968–1972)	Elesh (1977) (journal article)	Low‐income, husband‐wife families with one or more dependants (*N* = 732)	Number of hospital days, number of chronic illnesses (both reported separately for husband, wife, children), number of work days lost (husband, wife), number of days in bed (children) Measured at: 6 months, 1.5 years, 2.5 years for parents; 6 months, 2 years for children
			Kerachsky (1977) (book chapter)	Adult males and females from low‐income, husband‐wife families (*N* = 1293)	Number of times entered hospital, illness lasting more than 3 months, illness interfering with work (all reported separately for men and women), illness preventing work (men only) Measured at: 6 months, 1.5 years, 2.5 years
			Ladinsky (1977) (book chapter)	Low‐income husband‐wife families, male heads, husband‐wife families with children (depending on outcome) (*N* = 1001)	Home improvements, appliance and car purchases; social integration and recreation (10 variables described in Supporting Information: Appendix 5) Measured at: baseline, quarterly up to 3 years depending on outcome
			Mallar (1977) (book chapter)	Youths from working‐poor families with both parents present (*N* = 138)	High school completion, years of schooling attained Measured at: 3 years
			Middleton (1977) (book chapter)	Low‐income male household heads (*N* = 1166)	15 outcome variables on “social psychological effects” (see Supporting Information: Appendix 5 for details) Measured at: baseline, 1–3 years depending on outcome
			Nicholson (1977) (book chapter)	Low‐income families (*N* = 586)	16 economic outcome variables (see Supporting Information: Appendix 5 for details) Measured at: baseline, 1.5–2 years depending on outcome
**Rural Income Maintenance Experiment (RIME)** States of Iowa and North Carolina, USA	NIT with five configurations/combinations of a guarantee (50%, 75% or 100% of the poverty line) and withdrawal rate (30%, 50% or 70%): that is, 50/50, 75/30, 75/50, 75/70 and 100/50	RCT; allocation by household Duration: 3 years (1970–1973)	Maynard (1977) (journal article)	Children in grades 2 to 12 from low‐income rural families (*N* = 847)	Absenteeism, comportment, academic grade point average, standardized achievement test score (percentile and deviation from expected grade equivalent) Measured at: unknown^b^
			O'Connor (1979) (journal article)	Low‐income rural families (*N* = 612)	Quality of dietary intake Measured at: quarter 3, quarter 11 (2 years apart)
**Seattle‐Denver Income Maintenance Experiment (SIME/DIME)** Cities of Seattle (Washington State) and Denver (Colorado), USA	NIT with eleven configurations/combinations of a guarantee (95%, 120% or 140% of the poverty line) and withdrawal rate (50%, 70%, 70% declining^c^ or 80% declining^c^): that is, 95/50, 95/70, 95/70d, 95/80d, 120/50, 120/70, 120/70d, 120/80d, 140/50, 140/70 and 140/80d	RCT; stratified by income, race/ethnicity, and (in Denver only) marital status; allocation by household. Duration: 3–5 years^d^ (1971–1976)	Groeneveld (1979) (published report)	White and Black youths, aged 9 to 13 at enrollment (*N* = 1411)	Delinquency (contacts with police for status offenses and for serious offenses) Measured at: baseline, 5–6 years
			Manheim (1979) (conference proceeding – included report)	Children in grades 2–10 at enrollment (*N* = 765)	Grade point average, standardized test score, school absences Measured at: baseline, 2 years, 4 years
			Thoits (1979) (journal article)	Adult heads of households (*N* = approx. 7500)	Psychological distress score (self‐reported) based on adapted Macmillan Health Opinion Survey Measured at: 4/8 months, 20/24 months (males/females)
			Venti (1984) (journal article)	Youths aged 16–21 during the first three years of the experiment (*N* not reported; total of 4604 observations from 3 time points)	Probability of post‐mandatory schooling (self‐reported) Measured at: 5 months, 17 months, 29 months
**Stockton Economic Empowerment Demonstration (SEED)** City of Stockton, California, USA	USD500 per month regardless of other income (supplemental type of GBI)	RCT; allocation by individual; mixed methods with surveys for quantitative analysis and interviews for qualitative. Duration: 12 months (2019–2020)	West (2021) (published report)	Residents of Stockton, California in a neighborhood with a median income of $46,033 or less, aged 18+ (*N* = 325)	14 outcome variables: 4 economic, 3 mental/psychological, 6 physical health, 1 other (see Supporting Information: Appendix 5 for detailed list of all outcomes) Measured at: baseline, 6 months, 12 months

^a^ The capital letter “*N*” denotes the total sample size in each study.

^b^ Timing of outcome measurement is reported as “at the time of the most recent observations on any school performance measure” (Maynard, 1977, p. 371).

^c^ The Seattle‐Denver Income Maintenance Experiment also tested variable withdrawal rates that started at either 70% or 80% and declined by 2.5% for every $1000 of earned income (1970s USD).

^d^ The Seattle‐Denver Income Maintenance Experiment enrolled participants in either a 3‐year or 5‐year plan. A small sample (4%) was also enrolled in a 20‐year plan, but the experiment was canceled in 1982.

Six of the ten experiments provided NIT interventions, which varied within and between experiments in the guarantee amount and the “tax” (withdrawal) rate on benefits if other income was received. These experiments were analyzed in 21 included studies. The NIT configurations ranged in generosity from a guarantee of 50% up to 140% of the poverty line, with withdrawal rates ranging from 30% to 75%. The withdrawal rates with the less generous guarantees were limited to 50% or less, so that none of the NIT configurations had a low guarantee amount combined with a high withdrawal rate. The New Jersey experiment originally included one such arm, but it was removed because of very high attrition, and the remaining participants were reallocated to a more generous NIT arm (Kershaw & Fair, 1976a). The withdrawal rate of the Canada public pension varies with income level and marital status (McIntyre, 2016a), so we could not report a specific rate.

Three of the included studies reported on two experiments which provided a GMI, either with full withdrawal or partial withdrawal. Another three studies reported on two other experiments which provided a supplemental GBI as the intervention.

We excluded 15 studies, listed in Supporting Information: Appendix 4, which reported only outcomes that were ambiguous in their effect on poverty. Three other studies shown in this appendix were excluded following deliberations between the reviewers about the study designs and reaching consensus that the designs did not meet our inclusion criterion.

We also excluded two studies (McDowell, 2020, 2021) on the Ontario Basic Income Pilot (OBIP), conducted in Canada in 2018–2019. We initially included the OBIP studies as using cohort designs with historical data collected at one timepoint, as described by Parker and Berman (Parker & Berman, 2016); however, consensus was later reached among three reviewers (AR, LI, MMK) that both studies employed an ineligible cross‐sectional design with one time‐point. The OBIP pilot was canceled prematurely after a new government came into power, so meaningful follow‐up data was not collected by the pilot project's evaluation team (McDowell & Ferdosi, 2021).

##### Comparators

The participants in the control groups of all the included studies did not receive the intervention but could receive social assistance benefits if they met the eligibility requirements. As such, the control groups of the included studies received “usual care.”

##### Sources of data

All of the data reported in this review and used in meta‐analyses was obtained from the published reports of the included studies, with the exception of Calnitsky (2021) (for which data tables were obtained through correspondence with the study authors).

##### Funding

For most of the included studies, the funding was provided by different sources for the experimental stage (design, recruitment, intervention, data collection) and the study stage (analysis and reporting).

All of the included experiments were funded by governments (federal, provincial, state, or municipal), except the Stockton (SEED) experiment, which was funded by private donors and philanthropic organizations (SEED, 2019).

All of the included studies were supported by grants from governments or public research institutions, except the SEED study (West, 2021), which was funded by the Robert Wood Johnson Foundation.

### 6.2 Risk of bias in included studies

**Figure 3:**
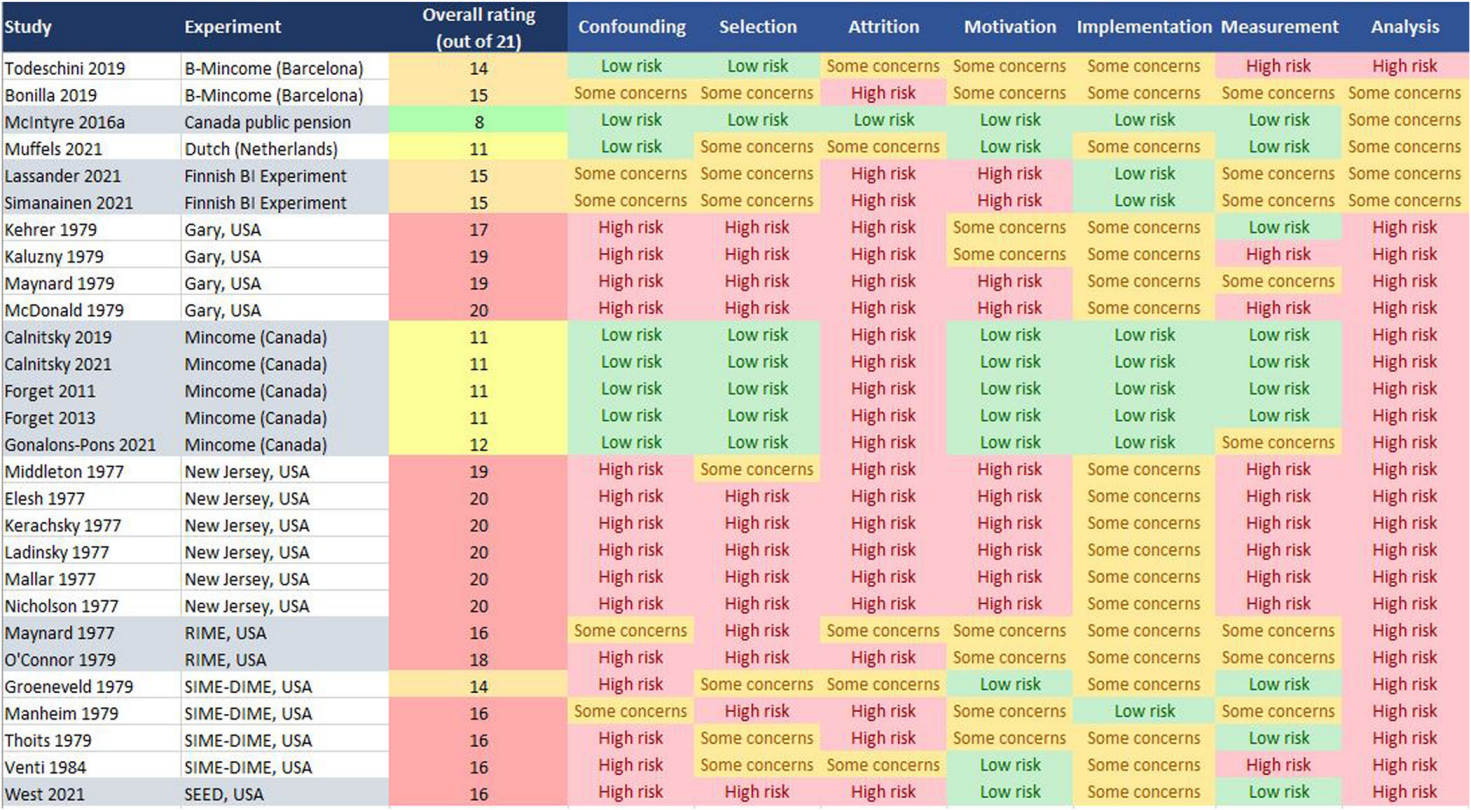
Risk of bias summary and overall score (higher score indicates higher overall risk of bias).

The risk of bias was assessed as moderate or high for most of the included studies. Figure 3 depicts a visual summary of the assessments. The overall rating in the third column uses a color‐coding scheme to visually represent the overall risk for each study. Although the thresholds for the color‐coding are arbitrary (overall score of 7–9 in green, 10–12 in yellow, 13–15 in orange, 16–21 in red), there is consistency in high‐risk ratings for the older U.S. NIT experiments (Gary, New Jersey, RIME, and SIME/DIME), even though the risk assessments were conducted by different pairs of reviewers (from a total of fourteen). Much of the high risk for these older studies is attributable to incomplete reporting of the methods used, with articles focusing on analysis methods and discussion of the results.

There were high rates of attrition in all of the experiments, except the Canada pension study, which contributed to judgments of high risk in 21 studies for the attrition domain.

The assessed risk for the analysis and reporting domain was high for 22 of the studies, mostly due to the unavailability of a pre‐analysis plan. This was typical where the “experiment” stage (from design to data collection) and the “study” stage (statistical analysis and reporting of results) were conducted by different research teams. Of the recent experiments (from the past decade), we located the pre‐analysis plan for the SEED experiment (SEED, 2019); however, specific methods and statistical details were lacking in the interim first‐year report (West, 2021) that was included in this review. We also located the pre‐analysis plan for the Finnish experiment (Hämäläinen, 2022), but it only referred to the analysis of administrative data (for employment outcomes) and not the survey data that we examined in this review.

Studies that used data from administrative sources (health departments, school boards, police records, etc.) tended to have better risk of bias ratings than those that analyzed survey data obtained in interviews with participants. High rates of attrition resulted in “some concerns” and “high risk” ratings for all the studies except McIntyre (2016a) (Canada public pension) which used a repeated cross‐sectional design, so participant retention after recruitment was not a concern.

We did not assess the risk of bias by outcome, which takes into consideration that some measures of treatment effect within the same study can be more subjective than others. This was a methodological oversight on our part. However, the included studies that reported both objective and subjective (self‐reported) outcome measures were all assessed as having a high risk of bias in domains other than “motivation bias” (i.e., the objectively measured outcomes could not have led to a low *overall* risk of bias rating). Thus, our interpretations of the findings would not have been different if we had conducted risk of bias assessments by outcome.

### 6.3 Synthesis of results

As anticipated, the diversity of intervention types and reported outcomes limited our ability to conduct a comprehensive quantitative synthesis. Therefore, the results are presented in tabular form by outcome category, along with meta‐analyses of specific outcomes which were reported in two or more studies.

Each of the results tables presents the statistically significant results (with *p* < 0.05) for the main study sample, as well as those reported for any subgroups that we could categorize according to the PROGRESS‐Plus framework, that would allow us to investigate equity‐related differences in intervention effects.

Due to the large number of outcomes, most of which were reported in single studies, we present the reported effect estimates, along with the standardized mean differences (*d*) where sufficient data was reported in the study to calculate them. For some outcomes, as shown in the results tables, the magnitude of the effect estimate was unclear because point estimates were not reported and there was insufficient data provided to calculate them. In these cases, only *p*‐values were available to indicate “significant” findings.

#### 6.3.1 Classification of outcomes

The included studies reported on only one of the two primary outcomes: food insecurity. The second primary outcome, poverty level assessed using official, national, or international measures, was not reported in any of the included studies.

Outcomes were categorized during the data extraction stage as one of the following: economic/material, physical health, mental/psychological health, education/training, and “other.” Food insecurity was considered an economic outcome (by its definition), but we examine it below as a separate category. As well, the outcomes that were originally categorized as “other” were found to belong to two distinct categories: social outcomes, and individual choice/agency outcomes. Therefore, we have summarized the study findings according to these seven categories in separate tables below.

The large number of outcome variables in each category (other than food insecurity) were grouped into subcategories, as shown in the results tables below, to help identify and match similar outcomes for potential meta‐analyses.

A total of 176 outcome variables (see Supporting Information: Appendix 5) were reported in the included studies: 3 in the food insecurity category, 38 were economic/material, 33 were on physical health, 39 were on psychological/mental health, 22 were social outcomes, 17 were educational, and 24 were on individual choice and agency. These outcomes met our inclusion criteria for poverty‐related outcomes that were not ambiguous in the direction of the effect on poverty (e.g., “number of physician visits” was excluded because it was unclear if more visits meant worse health or prioritizing one's health and being able to afford the time and expense to visit a physician). A total of 56 outcome variables were excluded (Supporting Information: Appendix 6) as either not poverty‐related (e.g., probability of moving) or ambiguous in their effect on poverty (e.g., marital dissolution).

We categorized the 176 included outcomes into 34 subcategories: one for food insecurity, six for economic/material, nine for physical health, seven for psychological/mental health, three for social, five for educational, and three for individual choice and agency. The outcomes within each of the 34 subcategories were then assessed to see which could be combined in meta‐analyses.

#### 6.3.2 Consideration of equity‐relevant factors in included studies

A summary of the PROGRESS‐Plus factors that were examined in subgroup analyses in the included studies is provided in Table 4. Out of the 27 included studies, 18 conducted subgroup analyses across one or more PROGRESS‐Plus categories. The following factors belonging to the “Plus” category were considered in at least on study: age, marital status, smoking/nonsmoking, and police record. Nine of the studies did not report any subgroup analyses by factors in the PROGRESS‐Plus framework.

**Table 4:** PROGRESS‐Plus factors examined in subgroup analyses within included studies.

Study/experiment	Place of residence	Race, ethnicity, or ancestry	Occupation	Gender or sex	Religion	Education	Social capital	Socioeconomic position	Plus factors
Bonilla (2019) B‐Mincome (Barcelona)		EU versus non‐EU ethno‐cultural background		Male, female		Education level	Amount of participation groups, support network	Income decile, material deprivation, food deprivation, bad house conditions	Age, marital status (married, single, divorced, separated, widowed)
McIntyre (2016a) Canada public pension	Urban, rural			Male, female				Income level, home ownership	
Kehrer (1979) Gary, USA									Age (<18, 18–34, >34); smoking, nonsmoking
Maynard (1979) Gary, USA								Pre‐enrollment family income (above or below half of poverty line)	
McDonald (1979) Gary, USA				Male, female				Family income above or below NIT breakeven point	
Calnitsky (2019) Mincome (Canada)				Male, female					Age (<26, 26–49, >49)
Calnitsky (2021) Mincome (Canada)						Percent of population with any postsecondary education		Population average family income	Age (percent of population between 20 and 24)
Gonalons‐Pons (2021) Mincome (Canada)	Dauphin (small town/rural), Winnipeg (city)								
Elesh (1977) New Jersey, USA				Male, female					
Kerachsky (1977) New Jersey, USA				Male, female					
Ladinsky (1977) New Jersey, USA	4 sites (3 cities in New Jersey, 1 city in Pennsylvania	Black, Spanish‐speaking, White	“Occupational prestige” (Duncan socioeconomic index)	Husbands, wives		“Education” (not defined)		Home‐ownership; family earnings	Age of adults
Nicholson (1977) New Jersey, USA		Black, Spanish‐speaking, White						Renter, homeowner	
Maynard (1977) RIME, USA	Iowa, North Carolina								
O'Connor (1979) RIME, USA	Iowa, North Carolina								Age of family head (<31, 31–50, 51–64
Groeneveld (1979) SIME‐DIME, USA	Denver, Seattle; change of residence	Black, White		Male, female				Family income	Police record; parent or sibling with police record; age (9–13 at baseline)
Manheim (1979) SIME‐DIME, USA	Denver, Seattle							Change in income due to the experiment	
Thoits (1979) SIME‐DIME, USA	Denver, Seattle	Black, Chicano, White		Male, female					Marital status (married, single)
Venti (1984) SIME‐DIME, USA	Denver, Seattle	Black, white		Male, female					Age (16, 17, 18, 19, 20, 21)

Intervention effects by race or ethnicity were examined in six studies. We report the original terminology as used by the study authors, even though some of the terms from the older studies may be outdated (e.g., “Chicano” instead of Mexican American).

#### 6.3.3 Results for primary outcomes

##### Food insecurity

Food insecurity outcomes were reported in only two of the included studies: McIntyre (2016a) (Canada public pension) and Todeschini (2019) (B‐Mincome). As shown in Table 5, both studies found large reductions in food insecurity. The Canada public pension study looked at low‐income single people aged 55 and up, and found a 54% reduction in food insecurity after they started to receive the public pension at the age of 65. For the B‐Mincome study, we only included arms that met our GBI criteria, so the conditional benefit arms were excluded. The observed decreases in the probability of food insecurity were 26.4% (*p* < 0.05) for the unconditional arm with full benefit withdrawal and 19.9% (*p* < 0.05) for the unconditional arm with partial withdrawal. For the two unconditional arms combined, the study found a decrease of 22.4% (*p* < 0.05).

**Table 5:** Results for food insecurity outcomes.

		Study findings	
Study	Outcome	Full study sample	By PROGRESS‐Plus factor
McIntyre (2016a)	Prevalence of food insecurity	Decrease of 54% (calculated *d* = −0.573, 95% CI: −0.653 to −0.493)	*Gender/sex:* 4.0% lower for male than female (*p* < 0.05) *SEP*: 10.4% to 14.1% lower for mid and upper low‐income than lowest low‐income bracket, *p* < 0.05); decrease with home ownership (11.3% less, *p* < 0.05)
Todeschini (2019)	Food insecurity	Decreased probability of 22.4% (*p* < 0.05) for all unconditional arms combined, decrease of 26.4% (*p* < 0.05) for unconditional with full withdrawal (GMI type^a^), decrease of 19.9% (*p* < 0.05) for unconditional with partial withdrawal, decrease of 29.9% (*p* < 0.01) for unconditional without activation policy	
	Going to bed hungry	Decreased probability of 12.0% (*p* < 0.05) for all unconditional arms combined, 13.8% (*p* < 0.05) for unconditional with full withdrawal (GMI type^a^), 16.3% (*p* < 0.05) for unconditional without activation policy option	

^a^ Guaranteed minimum income (GMI) type of GBI.

**Figure 4:**
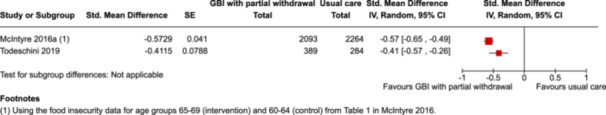
Effect of GBI with partial withdrawal on food insecurity.

The Canada public pension program uses an NIT approach, such that the benefit amount starts out at approximately 80% of the poverty line if there is no other income and then is gradually reduced as other income increases. The B‐Mincome experiment included several different intervention arms, one of which provided an unconditional guaranteed amount to cover the cost of basic needs, which was gradually reduced as income increased (i.e., GMI with partial withdrawal). Figure 4 shows a visual representation of the effect estimates of these two similar interventions. We did not calculate a pooled estimate because the interventions and populations were different. McIntyre (2016a) included only single Canadians aged 55 and up (around the public pension eligibility age of 65), and B‐Mincome targeted households in low‐income neighborhoods in Barcelona.

##### Poverty level assessed using official and international measures

None of the included studies used instruments or indexes such as official national poverty measures, consumption indicators such as the Household Budget Survey (HBS) and Consumer Expenditure (CE) Survey, or measures of deprivation such as the European Union's Material Deprivation (MD) Index. Individual components of these measures, such as food expenditure or housing quality, were included as secondary outcomes.

#### 6.3.4 Results for secondary outcomes

##### Economic and material outcomes

The outcomes in this category are summarized in Table 6. The individual outcomes were grouped into six subcategories as outlined in the subsequent paragraphs.

**Table 6:** Results for economic and material outcomes.

Outcome subcategory	Study	Outcome	Statistically significant results	
			Full study sample	By PROGRESS‐Plus factor
Financial hardship	Todeschini (2019)	Falling behind in mortgage repayments or rent	Decrease of 20.5% (*p* < 0.05) for unconditional arm without activation policy	
		Borrowing money from family or friends	Decrease of 7.3% (*p* < 0.05) for all unconditional arms combined, 7.5% (*p* < 0.05) for unconditional with partial withdrawal arm	
		Falling behind in utilities expenditures	‐‐	
		Forced to leave current residence	‐‐	
Material deprivation	Todeschini (2019)	Severe material deprivation	Decrease of 7.6% (*p* < 0.05) for all unconditional arms combined, 9.0% (*p* < 0.05) for unconditional with full withdrawal, 11.4% (*p* < 0.01) for unconditional without activation policy	
		Having roof leaks and moisture problems	Decrease of 8.5% (*p* < 0.05) for unconditional with partial withdrawal arm	
		Material deprivation	‐‐	
Personal finances	Nicholson (1977)	Financial assets	‐‐	*SEP*: increase for homeowners from −97 to 48 USD (1970 dollars, *p* < 0.05)
		Cash	‐‐	
		Home debt	‐‐	*SEP*: increase for homeowners of 19% (*p* < 0.05)
		Total nonhome debt	‐‐	*SEP*: decrease for homeowners from 590 to −71 USD (1970 dollars, *p* < 0.05)
		Auto debt	‐‐	
		Medical debt	‐‐	
		All other debt (non‐auto, nonmedical)	‐‐	*SEP*: decrease for homeowners from 696 to −39 USD (1970 dollars, *p* < 0.05); decrease for renters from 151 to 68 USD (1970 dollars, *p* < 0.05)
	Todeschini (2019)	Having outstanding debt	‐‐	
		Buffer for unexpected financial expenses	‐‐	
	West (2021)	Income volatility – monthly fluctuation	Decrease of 31% (statistical significance not reported, *p* < 0.05 is assumed)	
		Ability to cover a $400 emergency	Increase (52% in intervention group, 28% in control group; calculated *d* = 0.565, 95% CI: 0.307 to 0.822)	
Subjective financial well‐being	Lassander (2021)	Financial stress	Decrease of 6.6% (*p* = 0.014)	
		Financial management/control	Increase of 10.8% (*p* < 0.001)	
		Financial freedom	‐‐	
		Emergency funds	Increase of 6.3% (*p* = 0.009)	
	Muffels (2021)	Financial stress and poverty	‐‐	
	Todeschini (2019)	Satisfaction with economic situation (0–10 scale)	Increase of 1.1 points (*p* < 0.01) for all unconditional arms	
Food expenditure	Nicholson (1977)	Expenditures – Food eaten at home	‐‐	
		Expenditures – Food eaten out	‐‐	*SEP*: increase for homeowners of 39% (*p* < 0.05); **∇** decrease for renters of 19% (*p* < 0.05)
Nonfood expenditures	Kaluzny (1979)	Probability of becoming a homeowner	Increased probability during 1st year of 0.053 (*p* < 0.01)	
		Rental expenditure	Increase during 3rd year of 4.3% (*p* < 0.01)	
	Ladinsky (1977)	Lifestyle enhancement – home improvements and repair	‐‐	*SEP*: increase with home ownership at all quarters (*p* < 0.01); **∇** *Race/ethnicity*: decrease for Black at 7th and 11th quarters (*p* < 0.05)
		Lifestyle enhancement – value of appliances owned	‐‐	**∇** *Place*: decrease in Paterson, NJ^a^ at 6th and 10th quarters (*p* < 0.01); *SEP*: increase with home ownership at 6th quarter (*p* < 0.01); increase with family earnings at 10th quarter (*p* < 0.01); *Age*: decrease with age at 10th quarter (*p* < 0.01)
		Lifestyle enhancement – value of cars owned	‐‐	*Place*: increase in Trenton, NJ^a^ at 10th quarter (*p* < 0.05) **∇** *Race/ethnicity*: decrease for Black at 10th quarter (*p* < 0.05); *Education*: increase with education at 10th quarter (*p* < 0.05); *SEP*: increase with home ownership at 10th quarter (*p* < 0.05); Increase with family earnings at 6th quarter (*p* < 0.01); **∇** *Age*: decrease with age at 6th quarter (*p* < 0.01)
	Nicholson (1977)	Expenditures – Clothing	‐‐	
		Expenditures – Rent	‐‐	
		Expenditures – Total durables	‐‐	**∇** *SEP*: decrease for renters of 31% (*p* < 0.05)
		Expenditures – Autos	‐‐	**∇** *SEP*: decrease for renters of 43% (*p* < 0.05)
		Expenditures – Home production appliances	‐‐	*SEP*: increase for homeowners of 45% (*p* < 0.05)
		Expenditures – Furniture	‐‐	**∇** *SEP*: decrease for renters of 28% (*p* < 0.05)
		Expenditures – Other appliances	‐‐	*SEP*: increase for homeowners of 45% (*p* < 0.05)

*Note*: **∇** denotes an adverse result. ‐‐ denotes no statistically significant effect (i.e., *p* ≥ 0.05).

^a^ Trenton, NJ had the highest rate of families receiving social assistance benefits at 18%, followed by Scranton, PA at 15%, Paterson, NJ at 10%, and Jersey City, NJ at 5%.

###### Financial hardship

A single study examined outcomes related to financial hardship (Todeschini, 2019, B‐Mincome). Across all unconditional study arms, the probability of borrowing money from family or friends decreased by 7.3% (*p* < 0.05) with the unconditional partial withdrawal arm demonstrating results of a similar magnitude (7.5%, *p* < 0.05). Only the unconditional no activation study arm reported a significant decrease (20.5%, *p* < 0.05) in the likelihood of falling behind on mortgage or rent payments.

###### Material deprivation

Only Todeschini (2019) (B‐Mincome) examined outcomes within this subcategory. The probability of severe material deprivation in the unconditional study arms decreased by 7.6% (*p* < 0.05). The unconditional full withdrawal arm (9.0%, *p* < 0.05) and the unconditional no activation policy arm (11.4%, *p* < 0.01) were also independently significant. The unconditional partial withdrawal arm demonstrated a decrease in the probability of having roof leaks or moisture problems (8.5%, *p* < 0.05).

###### Personal finances

Three studies examined differing outcomes related to personal finances: Nicholson (1977) (New Jersey experiment), Todeschini (2019) (B‐Mincome), and West (2021) (SEED).

Nicholson (1977) found that with GBI, homeowners significantly increased their financial assets (−97 to 48 USD, 1970 dollars, *p* < 0.05), as well as home debt (19%, *p* < 0.05), while significantly decreasing their nonhome debt (590 to −71 USD, 1970 dollars, *p* < 0.05) and their debt from nonhome, none‐auto, and nonmedical sources (696 to −39 USD, 1970 dollars, *p* < 0.05). Whereas renters demonstrated significant results only in the latter outcome; significantly decreasing their nonhome, non‐auto, nonmedical debt from 151 to 68 USD (1970 dollars, *p* < 0.05).

Todeschini (2019) did not find any significant results relating to outstanding debt or to financial buffers reserved for unexpected expenses. Alternatively, West (2021) found that GBI significantly decreased income volatility by 31% (statistical significance level not reported; *p* < 0.05 is assumed, as reported for other outcomes) and increased ability to cover a $400 USD emergency (52% in intervention group, 28% in control group; calculated *d* = 0.565, 95% CI: 0.307 to 0.822).

###### Subjective financial well‐being

**Figure 5:**
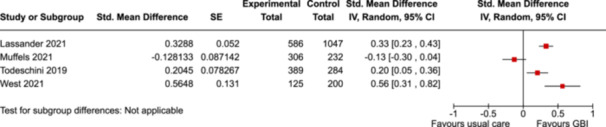
Effects of GBI on subjective financial well‐being.

Subjective financial well‐being was measured in three experiments: B‐Mincome (Todeschini, 2019), the Dutch experiments (Muffels, 2021), and the Finnish experiment (Lassander, 2021). Figure 5 shows a visual representation of the effect estimates from each study. For comparison purposes, we also included one outcome from the previous subcategory, “ability to cover a $400 emergency” (West, 2021) as a proxy for subjective financial well‐being. The only study that did not report a significant effect is Muffels (2021), which examined interventions in eight Dutch cities, three of which provided an eligible intervention (GMI with partial withdrawal).

###### Food expenditure

Food expenditure was examined by Nicholson (1977) (New Jersey experiment). GBI was found to be unrelated to at‐home food expenditures, but was linked to differences in food expenditures related to eating out. Homeowners demonstrated a 39% (*p* < 0.05) increase in eating‐out spending, whereas renters demonstrated a 19% (*p* < 0.05) decrease.

###### Nonfood expenditure

A diverse set of outcomes relating to nonfood expenditures was examined across three studies: Kaluzny (1979) (Gary experiment), Ladinsky (1977) (New Jersey experiment), and Nicholson (1977) (New Jersey experiment).

Kaluzny (1979) found that households receiving GBI increased their rental expenditure by 4.3% (*p* < 0.01) and had a 0.053 higher probability of becoming homeowners than the control group (*p* < 0.01).

Ladinsky (1977) examined three lifestyle enhancing outcomes quarterly across three cities for 3 years. Home improvements and repair significantly increased among homeowners receiving GBI across all quarters (*p* < 0.01) but decreased for Black participants at the 7th and 11th quarters (*p* < 0.05). The value of owned appliances increased with home ownership at the 6th quarter (*p* < 0.01) and increased along with family earnings at the 10th quarter (*p* < 0.01). Whereas the value of owned appliances decreased with age during the 10th quarter (*p* < 0.01 and decreased in Patterson (NJ) during both the 6th and 10th quarters (*p* < 0.01). The value of cars owned increased with home ownership, education, and in Trenton (NJ) at the 10th quarter (*p* < 0.05) and with family earnings at the 6th quarter (*p* < 0.01). However, the value of owned cars decreased with age at the 6th quarter (*p* < 0.01) and for Black residents at the 10th quarter (*p* < 0.01).

Nicholson (1977) found that homeowners enrolled in GBI increased spending on both home production appliances and other appliances by 45% (*p* < 0.05). Meanwhile, renters decreased spending on furniture, autos, and total durable products by 28%, 43%, and 31% respectively (*p* < 0.05).

##### Physical health outcomes

Nine studies of eight experiments investigated physical health outcomes: B‐Mincome (Todeschini, 2019), Gary (Kehrer, 1979), Mincome (Forget, 2011/2013), New Jersey (Elesh, 1977; Kerachsky, 1977), RIME/Rural (O'Connor, 1979), SEED (West, 2021), Finnish BI (Simanainen, 2021), and Dutch (Muffels, 2021). An overview of these outcomes, categorized into nine subgroups, is provided in Table 7.

**Table 7:** Results for physical health outcomes.

Outcome subcategory	Study	Outcome	Statistically significant results	
			Full study sample	By PROGRESS‐Plus factor
Child health (administrative data)	Forget (2011)	Small‐for‐gestational age, newborns	‐‐	
		Low birth weight	‐‐	
		At‐risk birth weight	‐‐	
	Kehrer (1979)	Birth weight	‐‐	*Age, smoking/not smoking, time between pregnancies*: Increase of 530 g (*p* < 0.01) for highest risk group (smoking, <16 months between pregnancies, age <18); **∇** decrease of 118 g (*p* < 0.01) for lowest risk group (not smoking, 16+ months between pregnancies, age 18–34)
Child health (self‐reported)	Elesh (1977)	Number of hospital days (children)	‐‐	
		Number of bed days (children)	‐‐	
		Number of chronic illnesses (children)	‐‐	
	Todeschini (2019)	Young people reporting bad health	Decrease of 21.4% (*p* < 0.05) for unconditional with partial withdrawal arm	
		New obesity diagnostics on people under 15 years	‐‐	
Physical health (administrative data)	Forget (2011/2013)	Total hospital separations (1978 vs. 1973)	Decrease of 19.2 per 1000 (95% CI: 17.1 to 21.3)	
		Total hospital separations, all causes	Decrease of 8.5% (*p* < 0.01)	
		Hospital separations, accidents and injuries	Decrease of 10% (*p* < 0.01)	
Physical health (self‐reported)	Elesh (1977)	Number of hospital days (husband and wife)	‐‐	
		Number of chronic illnesses (husband and wife)	‐‐	
	Kerachsky (1977)	Number of Times Entered Hospital – Adults	‐‐	*Gender/sex*: decrease for males during first year (out of three, (*t*(669) = 1.986; calculated *d* = 0.154, 95% CI: 0.002 to 0.306)
		Illness Lasting More Than 3 months – Adults	‐‐	
	Todeschini (2019)	Self‐reported serious health problems	‐‐	
	West (2021)	Energy over fatigue	Increase (*t*(186) = 7.30, *p* = 0.023, *d* = 0.335)	
		Pain (higher score is better)	Increase (*t*(189) = 7.87, *p* = 0.047, *d* = 0.283)	
Overall physical health (self‐reported)	Muffels (2021)	Subjective health	‐‐	
	Simanainen (2021)	Subjective state of health	Increase (*χ*² test *p*‐value = 0.051; calculated *d* = 0.146, 95% CI: 0.044 to 0.247)	
	Todeschini (2019)	Self‐rated health being good, very good or excellent	‐‐	
	West (2021)	General Health	‐‐	
Overall health and wellbeing	West (2021)	Overall Health and Wellbeing	‐‐	
Health‐related impairments/limitations	Kerachsky (1977)	Illness Interfering With Work	‐‐	
		Illness Preventing Work – Males Only	‐‐	
	Simanainen (2021)	Having a disease, disability or mental disorder that hinders daily life	Decrease (*χ*² test p‐value of 0.026; calculated *d* = −0.126, 95% CI: −0.227 to −0.025)	
	West (2021)	Physical functioning	‐‐	
		Role limitations due to physical health	‐‐	
		Social functioning (due to health)	‐‐	
Nutrition	O'Connor (1979)	Quality of dietary intake	‐‐	*Place*: increase in North Carolina^a^ (*t*(372) = 2.42, *p* = 0.02; calculated *d* = 0.251, 95% CI: 0.047 to 0.455)
Sleep	Todeschini (2019)	Quality of sleep	Increase of 7.6% (*p* < 0.05) for unconditional arm with partial withdrawal	
		Sleep deprivation – hours slept	‐‐	

*Note*: **∇** denotes an adverse result. ‐‐ denotes no statistically significant effect (i.e., *p* ≥ 0.05).

^a^ In North Carolina, 62% of families had pre‐enrollment incomes below the poverty line, compared to 37% in the other experimental site of Iowa.

###### Child health (administrative data)

Kehrer (1979) examined birth weight and identified a significant increase of 530 g (*p* < 0.01) for the highest risk group and a decrease of 118 g (*p* < 0.01) for the lowest risk group based on factors including age, smoking, and time between pregnancies.

###### Child health (self‐reported)

Todeschini (2019) study examined young people reporting poor health and found a significant decrease of 21.4% (*p* < 0.05) for the unconditional arm with partial withdrawal.

###### Physical health (administrative data)

Forget (2011/2013) investigated total hospital separations of 1978 in contrast to 1973 and found a decrease of 19.2 per 1000 (95% CI: 17.1 to 21.3). In addition, they discovered a decrease of 8.5% (*p* < 0.01) for all causes of total hospital separations, and a decrease of 10% for accidents and injuries (*p* < 0.01).

###### Physical health (self‐reported)

West (2021) explored “energy over fatigue” and pain, finding improvements in both (*p* < 0.05). In Kerachsky (1977), a gender‐based decrease was observed in the number of times males entered the hospital during the first out of 3 years (*d* = 0.154, 95% CI: 0.002 to 0.306). Simanainen (2021) reported an increase in subjective state of health (*χ*² test *p*‐value = 0.051).

###### Overall physical health (self‐reported)

**Figure 6:**
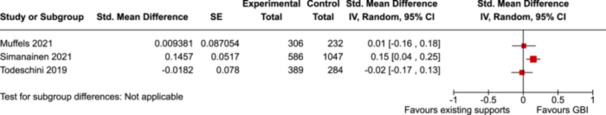
Effects of GBI on self‐rated overall physical health.

Self‐rated overall physical health was measured in four of the experiments: B‐Mincome (Todeschini, 2019), the Dutch experiments (Muffels, 2021), the Finnish experiment (Simanainen, 2021), and SEED (West, 2021). The effect estimates from the first three are visually represented in Figure 6. The result from SEED was reported only as nonsignificant, so we did not have the effect estimate and confidence interval to include in the forest plot.

The GBI interventions in B‐Mincome and the Dutch experiments both replaced most of the existing social assistance programs, whereas the intervention in the Finnish experiment was of the supplemental GBI type, so benefits were not reduced with increased income from other sources. The effect estimate from the Finland study is statistically significant but small (calculated *d* = 0.146, 95% CI: 0.044 to 0.247). The SEED intervention was also of the supplemental GBI type, but would likely have resulted in a smaller pooled estimate (if combined with the Finnish result), since it was not statistically significant.

###### Health‐related impairments or limitations

Simanainen (2021) also found a decrease in having a disease, disability, or a mental disorder that hinders daily life (*χ*² test p‐value of 0.026).

###### Nutrition

O'Connor (1979) assessed dietary intake quality, noting an increase by geographical location in North Carolina (*p* < 0.05; *d* = 0.251, 95% CI: 0.047 to 0.455).

###### Sleep

Todeschini (2019) reported a quality of sleep increase of 7.6% (*p* < 0.05) for the unconditional arm with partial withdrawal.

##### Psychological and mental health outcomes

The outcomes in this category were grouped into seven subcategories, as described in the following paragraphs. A summary of the outcomes is also presented in Table 8.

**Table 8:** Results for psychological/mental health outcomes.

Outcome subcategory	Study	Outcome	Statistically significant results	
			Full study sample	By PROGRESS‐Plus factor
Cognitive function (self‐rated)	Simanainen (2021)	Memory	Increase (*χ*² test p‐value of 0.001; calculated *d* = 0.212, 95% CI: 0.110 to 0.313)	
		Learning	Increase (*χ*² test *p*‐value < 0.001; calculated *d* = 0.281, 95% CI: 0.179 to 0.383)	
		Ability to concentrate	Increase (*χ*² test *p*‐value < 0.001; calculated *d* = 0.264, 95% CI: 0.162 to 0.365)	
Psychological well‐being (self‐rated)	Middleton (1977)	Perceived quality of life	‐‐	
	Bonilla (2019)	Life satisfaction (0–10 scale)	Increase of 0.92 points (calculated *d* = 0.35, 95% CI: 0.22 to 0.48)	*Race/ethnicity*: increase for non‐EU ethno‐cultural background (calculated *d* = 0.14, 95% CI: 0.01 to 0.27) *Sex/gender*: increase for female (calculated *d* = 0.21, 95% CI: 0.08 to 0.34) *SEP*: increase with income decile (calculated *d* = 0.21, 95% CI: 0.08 to 0.34) *Social capital*: increase with support network (calculated *d* = 0.12, 95% CI: 0.01 to 0.24)
	Muffels (2021)	Life satisfaction and subjective well‐being (0–10 scale)	‐‐	
	Simanainen (2021)	General life satisfaction (0–10 scale)	Increase of 0.5 points (*p* < 0.001)	
	Todeschini (2019)	General life satisfaction (0–10 scale)	Increase of 1.2 points (*p* < 0.01) for all unconditional arms combined	
		Being very satisfied with their life (score of >7 on 0–10 scale)	Increase of 15.4% (*p* < 0.01) for all unconditional arms combined, 16.1% (*p* < 0.01) for unconditional with full withdrawal, 17.5% (*p* < 0.01) for unconditional plus activation policy	
Mental health (administrative data)	Forget (2011/2013)	Hospital separations, non‐congenital mental health diagnoses	Decrease of 16% (*p* < 0.01)	
Mental health (self‐reported, composite score)	Simanainen (2021)	Clinical mental distress (MHI‐5 score below 53/100)	Decrease of 7.5% (*p* = 0.001)	
	Thoits (1979)	Psychological distress score (adapted Macmillan Health Survey)	‐‐	**∇** Reported for 30 different combinations of race/ethnicity (White, Black, Chicano), marital status (married, single), gender/sex (men, women), intervention length (3 or 5 years) and site (Denver, Seattle); 6 of 30 results were statistically significant, all indicating increased distress.
	West (2021)	Psychological distress score (Kessler 10 scale)	‐‐	
		Emotional health (Kessler 10 subscale)	Increase (*t*(183) = 14.85, *p* = 0.012, *d* = 0.370)	
		Emotional well‐being (Kessler 10 subscale)	Increase (*t*(191) = 7.70, *p* = 0.022, *d* = 0.332)	
Mental health (self‐reported, single item)	Middleton (1977)	General happiness	‐‐	
		Psychosomatic and nervous symptoms	‐‐	
		Self‐esteem	‐‐	
		Feeling of “nothing to do”	‐‐	
	Simanainen (2021)	I have been very nervous over the last 4 weeks	‐‐	
		I felt so low that nothing could make me feel better over the last 4 weeks	Decrease (*χ*² test *p*‐value = 0.003; calculated *d* = −0.210, 95% CI: −0.312 to −0.108)	
		I felt peaceful and calm over the last 4 weeks	‐‐	
		I felt sad and downcast over the last 4 weeks	Decrease (*χ*² test *p*‐value = 0.003; calculated *d* = −0.238, 95% CI: −0.341 to −0.136)	
		I have been happy over the last 4 weeks	‐‐	
		I have experienced depression	Decrease of 10.1% (*p* < 0.001)	
		I have experienced an inability to enjoy	Decrease of 11.5% (*p* < 0.001)	
		I experience loneliness	Decrease (*χ*² test p‐value of 0.032; calculated *d* = −0.171, 95% CI: −0.272 to −0.069)	
	Todeschini (2019)	Probability of developing a mental disorder (self‐reported)	‐‐	
		New diagnostics of anxiety and depression	‐‐	
Outlook	Middleton (1977)	Anomy	‐‐	
		Aspirations	‐‐	
		Control of future	‐‐	
		Community efficacy	‐‐	
		Expectation of better job in future	‐‐	
Worries	Middleton (1977)	Worry – money	‐‐	
		Worry – own health	‐‐	
		Worry – health of wife, children	**∇** Increase of 0.12 points (10‐point scale, *p* < 0.05)	
		Worry – raising children	‐‐	

*Note*: **∇** denotes an adverse result. ‐‐ denotes no statistically significant effect (i.e., *p* ≥ 0.05).

###### Cognitive functioning

Only one of the included studies (Simanainen, 2021, Finnish experiment) reported outcomes in this subcategory, and found statistically significant positive effects of the supplemental GBI intervention on memory (calculated *d* = 0.212, 95% CI: 0.110 to 0.313), learning (calculated *d* = 0.281, 95% CI: 0.179 to 0.383), and ability to concentrate (calculated *d* = 0.264, 95% CI: 0.162 to 0.365).

###### Self‐rated psychological well‐being

One study (Middleton, 1977, New Jersey experiment) examined perceived quality of life but did not find any significant effects of the NIT interventions.

**Figure 7:**
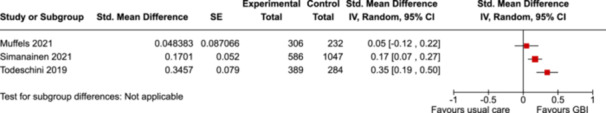
Effects of GBI on self‐rated life satisfaction.

Self‐rated life satisfaction was assessed in three experiments: B‐Mincome (Bonilla, 2019; Todeschini, 2019), the Dutch experiments (Muffels, 2021), and the Finnish experiment (Simanainen, 2021). The Bonilla (2019) study examined this outcome for the entire sample and did not report separate analyses by intervention arm. Since the B‐Mincome experiment included arms that were conditional on participation in “activation policies” (e.g., training or community projects), we did not include this study in the meta‐analysis shown in Figure 7.

We did not pool the effect estimates for this outcome because the interventions were different in each of the three experiments. The Todeschini (2019) study on B‐Mincome reported a significant result with the largest magnitude (*d* = 0.35, 95% CI: 0.19 to 0.50) for GMI with partial withdrawal arms only. The second‐largest result came from the Finnish experiment (Simanainen, 2021), while the Dutch experiments did not yield significant results at any of the municipal intervention sites (other than a negative one from a conditional (excluded) arm in the city of Oss). Bonilla (2019) reported the same effect estimate for the entire B‐Mincome sample as Todeschini (2019) reported for the unconditional/partial withdrawal arm, except with a slightly narrower confidence interval (*d* = 0.35, 95% CI: 0.22 to 0.48).

###### Mental health outcomes assessed using administrative data

One study (Forget, 2011/2013, Mincome) reported the outcome of hospital separations for non‐congenital mental health diagnoses, for which a 16% community‐wide reduction was found (*p* < 0.01) following the NIT intervention in the “saturation” site of Dauphin.

###### Self‐reported mental health assessed using survey composite scores

In this subcategory, the Dutch experiment (Muffels, 2021) did not find any significant effects of the GMI with partial withdrawal intervention on the MHI‐5 mental health index score.

**Figure 8:**
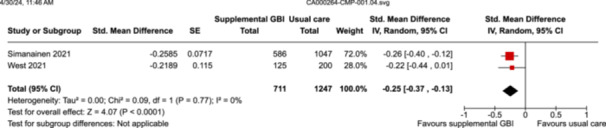
Effect of supplemental GBI on self‐rated mental distress.

For the outcome of self‐rated mental distress, we were able to pool the results of the Finnish experiment (Simanainen, 2021) and SEED (West, 2021), as both used the supplemental GBI approach in which benefits were not affected by changes in other income (Figure 8). On its own, the result from the SEED experiment was not significant at the 5% level (*p* = 0.056); however, the pooled SMD (with the larger Finnish experiment) is statistically significant (*d* = −0.25, 95% CI: −0.37 to −0.13), indicating a reduction in self‐rated mental distress. This outcome was also examined in SIME/DIME (Thoits, 1979), which provided NIT benefits in various configurations of guarantee and tax rate; however, this study did not yield consistent results across sites or subgroups.

##### Social outcomes

Table 9 summarizes the outcomes in this category, which was divided into three subcategories. The paragraphs below present the statistically significant findings for these outcomes. In most cases, we were not able to report the magnitude of the effect estimate in a meaningful way or calculate the SMDs due to missing statistical information in the included articles (e.g., regression coefficients reported without SEs, or sample sizes not reported).

**Table 9:** Results for social outcomes.

Outcome subcategory	Study	Outcome	Statistically significant results	
			Full study sample	By PROGRESS‐Plus factor
Social engagement	Ladinsky (1977)	Social Integration – social visits	Increase at 3‐year (final) timepoint (*p* < 0.01)	*Age*: decrease with age (*p* < 0.01)
		Social Integration – husband‐wife	‐‐	*Place*: decrease in Trenton, NJ^a^ (*p* < 0.05); Age: decrease with age (*p* < 0.01);
		Social Integration – family	‐‐	*Age*: decrease with age (*p* < 0.01)
		Social Integration – giving financial aid to friend or relative	Increase at 1‐year timepoint (*p* < 0.05)	*Place*: increase at 1‐year timepoint in all three New Jersey sites^a^ (vs. Scranton, PA; *p* < 0.05)
		Social Integration – membership in organizations	‐‐	*Place*: decrease in Jersey City, NJ^a^ (*p* < 0.01); *Education*: increase with education (*p* < 0.01);
		Social Integration – attendance at religious services	Increase at 7th quarter (*p* < 0.05)	*SEP*: increase with age and home ownership (both *p* < 0.01)
	Todeschini (2019)	Social participation	‐‐	
		Volunteering activities	**∇** Decrease of 8.1% (*p* < 0.05) for unconditional arm without activation policy	
		Electoral participation	‐‐	
		Participation in social leisure	‐‐	
Social perceptions	Muffels (2021)	Perceived extent of social integration	‐‐	
		Social trust	‐‐	
	Todeschini (2019)	Social support and stress – Duke Scale	‐‐	
		Confidence support	‐‐	
		Emotional support	‐‐	
		Total perceived support	‐‐	
Antisocial behavior	Calnitsky (2021)	Total crime rates	Decrease of 1438 per 100,000 (*p* < 0.01)	*Education*: decrease of 977 per 100,000 with percent increase in the population with any postsecondary ed. (*p* < 0.01)
		Violent crime rates	Decrease of 346 per 100,000 (*p* < 0.01)	*SEP*: decrease with average family income (*p* < 0.05, magnitude unclear)
		Property crime rates	Decrease of 726 per 100,000 (*p* < 0.01)	*Education*: decrease of 571 per 100,000 with percent increase in the population with any postsecondary ed. (*p* < 0.01)
		Other crime rates	‐‐	*Education*: decrease of 340 per 100,000 with percent increase in the population with any postsecondary ed. (*p* < 0.05)
	Groeneveld (1979)	Probability of delinquency – status offenses	‐‐	*Gender/sex and Plus factor*: Decrease of 0.11 (*p* < 0.01) for males with mother employed before experiment **∇** increase of 0.18 (*p* < 0.05) for males with prior police record
		Probability of delinquency – serious offenses	‐‐	*Gender/sex and Plus factor*: **∇** increase of 0.26 (*p* < 0.01) for females with sibling with police record; **∇** increase of 0.22 (*p* < 0.05) for males who changed residence

*Note*: **∇** denotes an adverse result. ‐‐ denotes no statistically significant effect (i.e., *p* ≥ 0.05).

^a^ Trenton, NJ had the highest rate of families receiving social assistance benefits at 18%, followed by Scranton, PA at 15%, Paterson, NJ at 10%, and Jersey City, NJ at 5%.

###### Social engagement

Outcomes in this subcategory were reported in two studies: Ladinsky (1977) (New Jersey experiment) and Todeschini (2019) (B‐Mincome experiment). Ladinsky (1977) observed a significant increase in social visits at the 3‐year timepoint (*p* < 0.01), which subsequently decreased with age (*p* < 0.01). In addition, they reported a rise in the provision of financial aid to friends or relatives at the 1‐year timepoint (*p* < 0.05), independent of geographical location (*p* < 0.01). Moreover, Ladinsky (1977) found that attendance at religious services increased significantly during the 7th quarter (*p* < 0.05), and this increase was positively associated with age and home ownership (*p* < 0.01). In contrast, Todeschini (2019) identified an 8.1% decrease in engagement with volunteer activities (*p* < 0.05).

###### Social perception

Muffels (2021) and Todeschini (2019) analyzed outcomes in this subcategory, but no significant findings were reported.

###### Antisocial behavior

Two studies (Calnitsky, 2021 and Groeneveld, 1979) examined outcomes related to antisocial behavior.

Calnitsky (2021) (Mincome experiment) focused on total, violent, property, and other crime rates. The results indicated a significant decrease in total crime rates of 1438 per 100,000 (*p* < 0.01). Education played a role, with a decrease of 977 per 100,000 associated with a percent increase in the population with any postsecondary education (*p* < 0.01). Violent crime rates decreased by 346 per 100,000 (*p* < 0.01), and a relationship with socioeconomic status (SEP) was observed, where average family income correlated with a decrease in violent crime rates (*p* < 0.05, magnitude not specified). Property crime rates decreased by 726 per 100,000 (*p* < 0.01), and education again showed an effect, with a reduction of 571 per 100,000 associated with an increase in postsecondary education (*p* < 0.01). For other crime rates, a decrease of 340 per 100,000 was linked to an increase in the population with any postsecondary education (*p* < 0.05).

Groeneveld (1979) (SIME‐DIME experiment) found a statistically significant decrease of 11% (*p* < 0.01) in the probability of delinquency for males with mothers who were employed before the experiment. However, a contrasting trend was observed in males with a prior police record. In this subgroup, the probability of delinquency demonstrated an increase of 18% (*p* < 0.05). Groeneveld (1979) also delved into the probability of delinquency related to more serious offenses. For females, those with a sibling having a police record exhibited an increase of 26% (*p* < 0.01) in the probability of delinquency. Among males, changing residence was identified as a significant Plus factor affecting the probability of serious offenses. The study discovered a 22% increase (*p* < 0.05) in the likelihood of delinquency for males who changed their residence during the experiment.

##### Educational outcomes

Educational and training outcomes were examined in seven studies on six of the experiments: B‐Mincome (Todeschini, 2019), Gary (Maynard, 1979), Mincome (Forget, 2011), New Jersey (Mallar, 1977), RIME/Rural (Maynard, 1977), and SIME/DIME (Manheim, 1979; Venti, 1984). A summary of these outcomes, grouped into five subcategories, is presented in Table 10.

**Table 10:** Results for educational outcomes.

Outcome subcategory	Study	Outcome	Study findings	
			Full study sample (IE denotes insufficient evidence of effect)	By PROGRESS‐Plus factor (for which evidence of effect was reported; otherwise none reported (NR))
Absenteeism	Maynard (1977)	Days absent	IE	*Place*: decrease in North Carolina^a^ of 30.4% (*p* < 0.05)
	Maynard (1979)	Days absent	IE	NR
	Manheim (1979)	Absence rate	IE	NR
Academic performance	Todeschini (2019)	Repeating course (grades 17/18 and 18/19)	IE	NR
	Maynard (1979)	Reading test score	Increase of 22 points (*p* < 0.01) for grades 4–6 (score range not reported)	*SEP*: larger increase (29.5 points, *p* < 0.05) for grades 4–6 if pre‐enrollment family income was below half of poverty line
	Maynard (1977)	Deviation from expected grade equivalent score on standardized achievement test	IE	*Place*: decrease (smaller deficit) in North Carolina^a^ of 18.9% (*p* < 0.05)
	Maynard (1977)	Standardized achievement test score – Percentile Score	IE	NR
	Maynard (1979)	Grade point average	**∇** Decrease of 1.6 points (*p* < 0.01) for grades 7–10 (score range not reported)	*SEP*: no decrease if family income was below half of poverty line; **∇** larger decrease (−2.2 points, *p* < 0.01) if family income was above half of poverty line
	Maynard (1977)		IE	NR
	Manheim (1979)		IE	NR
	Manheim (1979)	Standardized test score	IE	NR
Comportment	Maynard (1977)	Comportment grade point average	IE	*Place*: increase in North Carolina^a^ of 6.7% (*p* < 0.05)
School continuation	Todeschini (2019)	Continuing into post‐mandatory education	IE	NR
	McDonald (1979)		IE	*Gender/sex and SEP*: increase for males with below breakeven family income [*t*(135) = 2.33; calculated *d* = 0.406, 95% CI: 0.061 to 0.751]
	Forget (2011)	Grade 11/12 Enrollment	Increase of ~15% (*p* < 0.05 assumed)^b^	NR
	Mallar (1977)	Probability of high school completion	Increased probability (adjusted differential of 0.104 to 0.470) for six of eight NIT plans (*p* < 0.01), decrease (−0.113 to −0.140) for two of eight NIT plans (*p* < 0.01)	NR
	Mallar (1977)	Years of Schooling Attained	Increase (0.37 to 1.51 years) for six of eight NIT plans (*p* < 0.05)	NR
	Mallar (1977)	College attendance	IE	NR
	Venti (1984)	Probability of Schooling	Increase in probability of 0.088 or 25% (*p* < 0.05)	*Sex/gender*: larger increase for female (0.123, *p* < 0.05); *Race/ethnicity*: larger increase for White (0.140, *p* < 0.05); *Age*: larger increase for age 18 (0.140, *p* < 0.05)
Skills development	Todeschini (2019)	Number of persons in the household doing training	IE	NR

*Note*: **∇** denotes an adverse result. ‐‐ denotes no statistically significant effect (i.e., *p* ≥ 0.05).

^a^ In North Carolina, 62% of families had pre‐enrollment incomes below the poverty line, compared to 37% in the other experimental site of Iowa.

^b^ Magnitude is estimated from bar graph in Forget (2011); significance level is not reported for this outcome, but other significant outcomes are reported at *p* < 0.05.

###### Absenteeism

Three included studies addressed absences from education and training (Manheim, 1979; Maynard, 1977, 1979). Among these, only Maynard (1977) reported a statistically significant decrease in the number of days absent. This reduction, amounting to 30.4%, was observed in a specific geographical location (North Carolina) and was statistically significant (*p* < 0.05).

###### Academic performance

Maynard (1979) noted a significant increase of 22 points in reading test scores for students in grades four through six (*p* < 0.01). This improvement was even more pronounced among families with incomes below half of the poverty line (29.5 points, *p* < 0.05). Conversely, they discovered a significant decrease of 1.6 points in the grade point average for students in grades seven through ten (*p* < 0.01). Notably, this decrease was not evident among families below half of the poverty line, but they found a larger decrease of 2.2 points (*p* < 0.01) for families above half of the poverty line.

###### Comportment

Maynard (1977) also reported an increase in the comportment grade point average, specifically in the geographical location of North Carolina, amounting to 6.7% (*p* < 0.05).

###### School continuation

Five studies examined the subcategory of school continuation. McDonald (1979) reported an increase in the likelihood of males from families below the NIT breakeven income continuing into post‐mandatory education (calculated *d* = 0.406, 95% CI: 0.061 to 0.751). Additionally, Forget (2011) discovered an approximate 15% increase in grade 11 and 12 enrollments following the supplemental GBI intervention (*p* < 0.05). Similarly, Mallar (1977) found an increase in the probability of high school completion for six out of the eight NIT plans (*p* < 0.01) and a decrease in the remaining two plans (*p* < 0.01). Moreover, they observed an increase of approximately 1 year in the number of years of schooling attained for six of the eight NIT plans (*p* < 0.05). Venti (1984) identified a 25% increase in the probability of schooling (*p* < 0.05), which was even more substantial among female and White individuals (*p* < 0.05). Todeschini (2019) also found an increase in continuation to post‐mandatory education when families received UCTs (without an activation policy offered or required), though only at the 10% significance level.

##### Individual choice and agency outcomes

Table 11 summarizes the outcomes for individual choice and agency, grouped into three subcategories. The paragraphs below describe the statistically significant findings for these outcomes.

**Table 11:** Results for individual choice and agency outcomes.

Outcome subcategory	Study	Outcome	Statistically significant results	
			Full study sample	By PROGRESS‐Plus factor
Use of time – recreation and entertainment	Ladinsky (1977)	Leisure Activities – parks and zoos, movies, restaurants, and bars	**∇** Decrease at 9 months (*p* < 0.05)	**∇** *Race/ethnicity*: decrease for Spanish‐speaking at 21 months (*p* < 0.01); *Education*: Increase with education at 21 months (*p* < 0.01)
		Leisure Activities – involvement in hobbies, sports activities, and vacations	‐‐	**∇** *Age*: decrease with age at 21 months (*p* < 0.01)
	Todeschini (2019)	Participation in individual leisure	‐‐	
Choice/agency	Calnitsky (2019)	Reason for not working – any reason	‐‐	*Gender/sex*: increase for females of 11.7% (95% CI: 2.7% to 20.7%)
		Reason for not working – family	‐‐	
		Reason for not working – job/work conditions	‐‐	*Gender/sex*: increase for females of 11.3% (95% CI: 0.5% to 22.1%)
		Reason for not working – unpaid vacation	‐‐	
		Reason for not working – education	‐‐	*Gender/sex*: increase for females of 9.0% (95% CI: 0.4% to 17.6%)
		Reason for not working – did not want to work	Increase of 4.0% (95% CI: 0.4% to 7.6%)	*Age*: increase for ages 26–49 of 5.9% (95% CI: 1.7% to 10.1%)
		Reason for not working – ill or disabled	‐‐	
		Reason for not working – self‐employed	‐‐	
		Reason for not working – retired	‐‐	
		Reason for not working – other/unknown	‐‐	
	Muffels (2021)	Perceived capabilities, freedom of choice	‐‐	
Agency (wife)	Gonalons‐Pons (2021)	Bargaining and decision‐making power – on wife's job	‐‐	
		Bargaining and decision‐making power – on important decisions	‐‐	
		Bargaining and decision‐making power – who wins out	‐‐	
		Financial disagreement index (6‐item scale)	‐‐	*Place*: decrease in Winnipeg/urban (*p* < 0.05)
		Financial disagreement – have enough money	‐‐	*Place*: decrease in Winnipeg/urban (*p* < 0.05)
		Financial disagreement – save or spend	‐‐	*Place*: decrease in Winnipeg/urban (*p* < 0.05)
		Nonfinancial disagreement index (7‐item scale)	‐‐	
		Nonfinancial disagreement – husband's habits	‐‐	*Place*: decrease in Dauphin/rural (calculated *d* = −0.526, 95% CI: −0.785 to −0.267)
		Nonfinancial disagreement – religious beliefs	‐‐	*Place*: decrease in Winnipeg/urban (calculated *d* = −0.575, 95% CI: −0.814 to −0.336)
		Nonfinancial disagreement – choice of friends	‐‐	*Place*: decrease in Winnipeg/urban (calculated *d* = −0.644, 95% CI: −0.884 to −0.403)

*Note*: **∇** denotes an adverse result. ‐‐ denotes no statistically significant effect (i.e., *p* ≥ 0.05).

###### Use of time – Recreation and entertainment

Ladinsky (1977) (New Jersey experiment) found a significant decrease in leisure activities such as visiting parks and zoos, going to movies, restaurants, and bars at 9 months. Furthermore, they revealed differences across PROGRESS‐Plus factors. For instance, Spanish‐speaking individuals showed a more substantial decrease at 21 months (*p* < 0.01). On the other hand, engagement in hobbies, sports activities, and vacations did not show any significant results overall. However, age seemed to play a role, with a decrease in involvement observed with increasing age at 21 months (*p* < 0.01).

###### Choice/agency

Regarding agency and choice, Calnitsky (2019) (Mincome experiment) explored the reasons for not working. The study found that females experienced an increase in deciding not to work due to family reasons with an increase of 11.7% (95% CI: 2.7% to 20.7%), Similarly, job/work conditions were a consideration with a rise of 11.3% (95% CI: 0.5% to 22.1%), and education with an increase of 9.0% (95% CI: 0.4% to 17.6%). In addition, the study found a 4.0% increase (95% CI: 0.4% to 7.6%) in individuals who did not want to work, with a more significant increase observed in the age group of 26 to 49 years, which was 5.9% (95% CI: 1.7% to 10.1%).

###### Agency (wife)

Only one study examined outcomes in this subcategory. In terms of financial disagreements, Gonalons‐Pons (2021; Mincome experiment) observed a significant decrease within geographical locations (Winnipeg/urban areas) in the financial disagreement index, having enough money, and deciding to save or spend (all with *p* < 0.05). In addition, they found that nonfinancial disagreements showed a decrease in Dauphin/rural areas related to husband's habits (calculated *d* = −0.526, 95% CI: −0.785 to −0.267) as well as the Winnipeg/urban areas concerning religious beliefs (calculated *d* = −0.575, 95% CI: −0.814 to −0.336) and choice of friends (calculated *d* = −0.644, 95% CI: −0.884 to −0.403).

## 7 DISCUSSION

### 7.1 Summary of main results

This systematic review examines the effects of GBI interventions on poverty‐related outcomes in developed high‐income countries. Using very specific inclusion criteria, we identified 10 experiments and 27 related studies examining GBI interventions that resemble how GBI could potentially be implemented as full‐scale programs. Therefore, we excluded studies of interventions, policies, or programs that were conditional, not cash‐based, or not paid in regular, fixed or predictable amounts. As a result, the evidence examined in this review was more applicable to real‐world GBI proposals.

#### 7.1.1 GBI typology

We developed a framework for categorizing GBI approaches to assist in the evaluation and synthesis of the various approaches. We discovered that this was essential for a meaningful comparison of empirical evidence because each type of GBI approach provides financial assistance differently. To compare the effects of GBI interventions without classifying them by their type would be similar to conducting an evidence synthesis of drug trials testing the efficacy of either aspirin, ibuprofen, or acetaminophen, but not knowing which drug was given in each trial.

Based on the empirical and conceptual versions of GBI that we found in the literature on GBI, we identified five general types: subsistence‐level fixed amount, supplemental fixed amount, GMI with full benefit withdrawal, GMI with partial benefit withdrawal, and NIT.

The actual benefit amount varies within each GBI type, and once the type is identified, the next step in future research could be to examine “dose” responses (i.e., the effects of various benefit amounts of one GBI type). This was attempted in the early NIT experiments by providing varying guarantee levels and withdrawal rates to different subgroups of participants. Unfortunately, the experiments were so complex that even statisticians later debated over the appropriate analysis methods to use. One of the researchers involved in the SIME/DIME experiment wrote the following: “We decide on research questions; we get together to develop the survey instruments; and then, almost as an afterthought we realize that we have to process the data. We are finally learning that the design of the data processing and [of] the data management system has to be done before the rest of the design of the project” (Bell, 1979, p. 52).

Due to the considerable diversity of reported outcomes and the limited reporting of statistical data for each intervention arm in the included studies, we did not have enough information to examine dose responses by GBI type. However, this will likely be possible in the near future because many more GBI experiments are currently underway. In the U.S., 100 guaranteed income pilots have been announced or started since 2017, involving a total of 38,000 participants (Economic Security Project, 2022). In Germany, the privately funded Mein Grundeinkommen (My Basic Income, Mein Grundeinkommen, 2023) was launched in 2020 and will provide €1000 per month to 1500 recipients for 3 years. The Government of Ireland (2022) has also launched a 3‐year Basic Income for the Arts pilot which pays €325 per week to 2000 recipients. And as described above, two pilots in England (planned) and Wales (now underway) will test subsistence‐level, fixed‐benefit GBI.

While many of these ongoing pilots are limited by small sample sizes, the absence of control groups, and the targeting of specific demographic groups, which may limit the accuracy and generalizability of the findings, the larger pilots with controlled experimental designs will likely provide data useful for meta‐analyses. The results of these studies will also be easier to understand, compare, and meta‐analyze (possibly including dose responses) if they are categorized by the type of GBI using the framework presented above.

#### 7.1.2 Primary outcomes

##### Food insecurity

Food insecurity was only examined in two included studies: McIntyre (2016a) on the Canada public pension, and Todeschini (2019) on the B‐Mincome pilot. Although the first one used a quasi‐experimental design (repeated cross‐sectional) examining individuals, and the other was an RCT with household allocation, both studies reported substantial reductions in food insecurity when participants received a GBI. The Canada public pension is based on an NIT approach, while B‐Mincome tested the two types of GMI: one with full (dollar‐for‐dollar) withdrawal of benefits, and the other with partial withdrawal, which is similar to NIT. We did not include the full‐withdrawal arms in our synthesis because, while there were no conditions for receipt of benefits, we consider this approach to be a quasi‐GBI because it doesn't meet the criterion of providing an income base for all recipients (e.g., if other income reaches 98% of the guarantee amount, the benefit would be reduced by 98%).

B‐Mincome also included intervention arms with “activation policies”: training and employment, social entrepreneurship, room rental (by homeowners), and community participation. In the conditional arms, participants were required to engage in one of the four policies to be eligible for benefits, while in the *unconditional*‐with‐activation‐policy arms, participants were assigned to one of the four policies, but participation was optional. In the “cash transfer only” arm (Todeschini, 2019), participants received the benefits unconditionally without being assigned to any of the activation policies.

For all the unconditional arms in B‐Mincome (i.e., those included in this review), the mean food insecurity score at the final timepoint was 0.224 points lower on a 10 point scale than for the control group (*p* < 0.05). For the unconditional arms with assigned (but optional) activation policies, the result was not statistically significant; however, the largest reduction (0.299 points, *p* < 0.01) of all the subgroups was observed with the unconditional arm with no activation policy (cash transfer only). Interestingly, Todeschini (2019) reported that the participation rate was the same for the training and employment programs (81%) whether they were mandatory (conditional arm) or optional (unconditional arm), and the change in food insecurity was not statistically significant for either of these arms, compared to the control group. One possible explanation is that participants who engaged in the training and employment programs incurred work‐related expenses that cut into their household food budget. This reallocation of income, if it occurred, would be relevant to the problem of in‐work poverty.

##### Official, national, and international poverty measures

As reported above, we did not find any GBI studies that used national poverty measures (official or alternative), or internationally used poverty indexes or multidimensional measurement scales.

#### 7.1.3 Secondary outcomes

##### Economic and material

The results in this category were mostly favorable with respect to poverty outcomes, which would be expected if interventions provide more income than the existing social assistance programs. Adverse results were rare and may have been due to multiple hypothesis tests for different outcomes and subgroups of participants. Many of the included studies performed over twenty tests of statistical significance on the data, so with a significance level of 5% it is likely that at least one result that appears significant is due to chance. There were no adverse results when the entire sample was analyzed.

Subjective financial well‐being was examined in three studies, along with “ability to cover a $400 emergency” in a fourth. The only study that did not find a significant improvement evaluated the Dutch experiments, in which the amount of the GBI benefit was equal to the existing social assistance benefit, except that the GBI benefit was given unconditionally. The other three studies (on B‐Mincome, the Finnish experiment, and SEED), which provided higher amounts than social assistance, found statistically significant improvements.

##### Physical health

Improvements were reported for some specific health‐related outcomes (energy over fatigue, pain, hospital separations, quality of sleep), but no change in general health was found, except in one study. In the Finnish experiment, a statistically significant result was found for self‐rated health; however, the Stockton study (SEED) which provided a similar supplemental GBI intervention did not find a significant effect. The Finnish study also found an improvement in a similar outcome variable, “having a disease, disability or mental disorder that hinders daily life,” The absence of a significant result for overall health in the U.S. SEED study may be due to population differences between the two experiments.

There is also the question of whether a limited‐duration experiment can have an impact on overall health. The Forget (2011/2013) study of Mincome in Canada found significant reductions in hospital separations in the “saturation” site of Dauphin, reductions which persisted for several years after the experiment ended even though only about 20% of the Dauphin residents received the GBI benefit. The study authors proposed that people with the lowest income had poorer health and needed to be hospitalized more often, so improvements in their health may explain the observed reduction. On the other hand, Green (2022) conducted a reanalysis of the data used by Forget, controlling for “pre‐trends,” and found that the reduction in hospitalizations was part of a long‐term trend and not due to the Mincome benefits.

##### Psychological and mental health

The results in this category were more conclusive, especially from the newer experiments (B‐Mincome, Finland, and SEED). The two studies of the Dutch experiments did not find significant results, as was the case with the economic and material outcomes, which again may be due to the similarity of the intervention to the existing social assistance programs in the eight Dutch sites.

The older U.S. NIT experiments did not find improvements in psychological outcomes. The three included studies actually found a few adverse results, although these may also have been due to chance, considering the large numbers of subgroup analyses that were conducted on numerous outcome variables.

##### Social outcomes

Overall, the included studies did not yield many significant or conclusive results for social engagement. There were increases in some aspects of social integration in the New Jersey experiment, but they were not consistent (i.e., observed either at the midpoint or endpoint, but not both). In B‐Mincome, there was a reduction in the probability of volunteering for participants who received GBI benefits and who were not assigned to an activation policy (mandatory or optional).

In the Mincome saturation site of Dauphin, Manitoba, significant reductions in crime rates were found, especially for property crimes. While only about 20% of the residents enrolled and received the NIT benefits, crime rates are higher in low‐income settings and most of the recipients were residents with low incomes, so it is conceivable that the GBI benefits reduced the economic motives for people who may otherwise have committed break‐and‐enter and auto thefts (Calnitsky, 2021).

##### Educational outcomes

GBI interventions did not appear to have significant effects on academic performance, other than in the Gary experiment where the largest improvement in reading test scores in grades 4 to 6 was found for children from the lowest income families (less than half of the official poverty level). The limited duration of the experiments may limit the ability to assess whether GBI can affect academic performance, as success in higher grades may even depend on the level of poverty in early childhood.

The results for post‐compulsory school continuation were more compelling, with all five studies finding that more children stayed in school longer after the compulsory age for school attendance. McDonald (1979) reported results by income level and found a significant increase for males from families with incomes below the NIT breakeven amount. The intervention group also included families with higher incomes to avoid truncation bias; however, they would not have received NIT benefits unless their incomes decreased during the experiment. As such, the findings for the low‐income group are more relevant to this review.

The Todeschini (2019) study found an increase in post‐compulsory education in the group that received benefits unconditionally, without a mandatory or optional activation policy (“cash transfer only”). The result was reported as significant at the 10% level (calculated *d* = 0.09, 95% CI: −0.07 to 0.25), which isn't a strong finding on its own, but adds to the evidence from the other four studies, where the results were statistically significant at the 5% level.

School continuation is an important result for poverty reduction because the high‐school dropout rate is much higher for children from low‐income families (NCES, 2017), and the amount of income that people earn after graduating is highly correlated with their level of education. For example, the median full‐time earnings of 25‐to‐34‐year‐olds in the U.S. who had not completed high school was $29,800 in 2020, which is exactly one half of the $59,600 median earned by the same age group with a bachelor's degree (NCES, 2021). Thus, if GBI benefits allow children to stay in school longer, the long‐term effects on poverty reduction may be substantial.

##### Individual choice and agency

The effects in this category were not statistically significant overall for the entire study samples, although there were some differential effects found when subgroup results were examined, as described in the following section.

#### 7.1.4 PROGRESS‐Plus factors

Considering the socioeconomic determinants of poverty, the included experiments and studies did not investigate subgroup effects to a large extent. Interestingly, the older experiments seemed to consider differential effects across subgroups more than the newer ones; however, the choice of measures in the 1970s experiments is somewhat outdated now (e.g., “occupational prestige”), as are the gender references (e.g., two‐parent families referred to as male‐headed) and the division by race and ethnicity (e.g., Black, Spanish‐speaking, and White). Some of the researchers involved with the design of the U.S. NIT experiments did recognize the limitations of the latter: “The white group is surely heterogeneous ethnically” (Long, 1972, p. 95); however, all four of these experiments still operationalized race and ethnicity as “White,” “Black,” and sometimes a third choice depending on the U.S. setting (Puerto Rican, Chicano, or Hispanic).

The most frequent subgroup analyses that aligned with PROGRESS‐factors were in the categories of place of residence, race/ethnicity, sex and gender, and socioeconomic position. In the Plus category, age was the most commonly considered factor.

Place of residence was usually considered when the experiment was conducted in multiple sites, which was the case for the older NIT experiments in the U.S. and Canada. Some of the sites in each experiment had higher rates of poverty, and the estimated effects usually indicated larger improvements for some outcomes (quality of dietary intake, academic test score, absence from school and comportment, value of car owned).

Findings across race and ethnicity were generally inconsistent, with some exceptions. In the Seattle‐Denver experiment, White youths were found to stay in school longer than nonwhite youths, which may reflect the additional obstacles to education for non‐White youths aside from economic constraints. Since this experiment was conducted (1971–1976), dropout rates for non‐White children in the U.S. have fallen by more than half (NCES, 2019), so the experimental result may no longer be relevant. In the Bonilla (2019) study of B‐Mincome, self‐rated life satisfaction was found to be higher among participants with non‐EU ethno‐cultural backgrounds. This may be due to worse overall poverty in the countries from which they emigrated being their point of reference.

In terms of gender and sex, the B‐Mincome study also found significantly higher self‐rated life satisfaction among women than men, although the study authors do not elaborate on possible reasons for this. The Canada public pension study found that men were less likely to be food insecure than women after receiving the GBI‐like pension. The Calnitsky (2019) study on Mincome in Canada found that women receiving GBI benefits reported that they were less likely to work for reasons related to job/work conditions or choosing to pursue education. In 2022, the median hourly wage for women in Canada was 83.7% of the median for men (Statistics Canada, 2023), so the result from Mincome may still hold today, that women would make use of GBI benefits to attain a higher level of education and find better paying jobs.

The differential effects across socioeconomic position were based on the extent of financial constraints: higher incomes and more assets (e.g., home ownership) were associated with less food insecurity, less nonhome debt, higher values of appliances and cars, and greater life satisfaction.

The Plus factor of age was not found to significantly affect the magnitude or direction of outcomes, other than in reduced social integration with higher age, and more participants aged 26 to 49 giving the reason for not working as “did not want to work.” Both of these findings were from NIT experiments in the 1970s, so it is uncertain if they can be generalized to the present time.

### 7.2 Overall completeness and applicability of evidence

In general, the evidence from the included experiments and studies is directly relevant to the reduction of poverty using GBI approaches. All the reported outcomes relate to different aspects of poverty – economic, health‐related, psychological, social, and individual. What we did not anticipate was the great diversity in the outcome variables examined and reported in these experiments and studies. As such, the evidence base is expansive but very “thin,” with most outcome variables reported in only one study. Taken as a whole, though, the body of evidence still suggests that GBI interventions can lead to favorable outcomes in regard to poverty reduction.

Because of the potentially confounding impacts of the COVID‐19 pandemic, we excluded studies on experiments conducted since March 2020. One of the included studies (West, 2021) examined the first year of the Stockton Economic Empowerment Demonstration (SEED) experiment, which ended in February 2020. The final report (West & Castro, 2023) of the entire 2‐year experiment was published recently; however, the data were analyzed in separate stages for the first year and the second (March 2020 to February 2021). The second stage addressed “COVID questions” and the associated “environmental, and health threats” (p. 228), and thus was not within the scope of this review, which examined GBI interventions that could inform long‐term policies and programs for reducing poverty.

### 7.3 Quality of the evidence

Based on the GRADE assessments of the included outcomes, the quality of the evidence is moderate to low, as shown in the summary of findings table (Section 2). We assessed the two primary outcomes and five secondary outcomes which were examined in more than one included study. This comprised the maximum of seven outcomes to include in a summary of findings table (Schünemann, 2019).

In addition to being reported in multiple studies, the five secondary outcomes were selected on the basis of relevance to the present time, so at least two studies that reported the same outcome had to be relatively recent. For example, three of the NIT experiments from the 1970s examined grade point averages of youths, but the results may not be applicable to the present time because the welfare program that the control groups were eligible for, Aid to Families with Dependent Children (AFDC), was less stringent than the current Temporary Assistance for Needy Families (TANF), introduced in 1996 (Parolin, 2021).

The primary outcome of food insecurity level was reported in two studies, one using an RCT design (Todeschini, 2019), and the other using a repeated cross‐sectional design (McIntyre, 2016a). In GRADE assessments, non‐randomized studies are normally given an initial rating of “low” (i.e., downgraded two levels from “high”) because of “the inherent risk of bias associated with the lack of randomization, namely confounding and selection bias” (Schünemann, 2019, p. 391). However, we assessed the risk of bias in these two domains as being low in the McIntyre study, so we assigned an initial rating of “high” for the quality of evidence for the food insecurity outcome, and then arrived at a final rating of “moderate” as described in the summary of findings table.

None of the included studies reported results for the second primary outcome: poverty level measured using official, national, or international measures. Therefore, the summary of findings table contains six of the 176 eligible outcomes reported in the included studies. For the other 170 outcomes, GRADE assessments would have yielded ratings of either low or very low for each one. This is due to two reasons: risk of bias concerns and inconsistency across studies, the latter of which cannot be determined based on only one or two studies. Each of these two reasons reduces the GRADE rating by one level, so ratings of high or moderate certainty were not attainable for these studies. Additionally, imprecision (i.e., wide or unreported confidence intervals) of some of the effect estimates would have further reduced the rating from “low” to “very low.” Thus, it would not have been productive to conduct and report individual assessments for each of the 170 outcomes.

### 7.4 Limitations and potential biases in the review process

The strategies used to identify studies through published articles and sources as well as through gray literature were thorough and exhaustive. We ran comprehensive and sensitive searches across 16 databases, and we consulted various other resources, including websites of specific conferences, organizations, and governments. In addition, hand searching and citation searching were done to ensure that as many relevant studies as possible could be identified. However, we acknowledge that search strategies and techniques are prone to subjectivity and may have been designed differently, which means that some studies could have been missed. Nevertheless, we do feel confident that the approaches used to identify studies were rigorous and comprehensive. These approaches were all done between May and December 2022, and we do recognize that newer studies published since January 2023 may not have been included in this current review. At the same time, the results of recently conducted studies would be difficult to interpret because of the economic after‐effects of the pandemic, especially the rapid inflation in food, housing and energy prices in 2021 and 2022 (Causa, 2022).

A potential limitation of our search strategy was to not include “pensions” among our search keywords, to identify studies similar to McIntyre (2016) on public pensions in countries other than Canada. Although we used various hand searching strategies to identify eligible studies of interventions similar to GBI, it is possible that a more comprehensive search would have identified some relevant studies. Still, we do not believe that this would have significantly augmented the existing, sparse body of evidence on GBI, as much more research is also needed on populations below the pension age.

Another limitation was that half of the included experiments were completed over 40 years ago, so we were unable to acquire some of the full‐text articles that were cited in secondary sources, such as overview articles and summary reports on the experiments. Although efforts were made to request these items via interlibrary loan, some materials were still not accessible. It is not clear why some of the primary sources were no longer available in electronic form while others were. Possibly there is a “survivor” bias at play, such that the more interesting articles have survived and have been digitized.

The availability of articles on the older experiments also depended on the particular experiment. For example, we were able to obtain hard copies of the final report of the New Jersey experiment in book form (Kershaw & Fair, 1976a, 1976b), in which some individual chapters were the primary research output. We also found a digitized, online version of the Seattle‐Denver final report (SRI, 1983), which also contained some primary research reports. For the Gary and Rural/RIME experiments, we were only able to locate primary sources in the form of journal articles that were available online.

The age of the above‐mentioned studies is also a limitation in regard to the generalizability of the findings, due to the temporal differences in both the economic conditions (e.g., less precarious work, different levels of unemployment) as well as the primary welfare programs available (TANF vs. AFDC).

One of the major limitations to the strength of this review's findings was the high risk of bias in the included studies, which was due to high rates of attrition, subjective (self‐reported) and unblinded measurements, and the absence of pre‐analysis plans for most of the studies. This, combined with the sparse number of studies for each type of GBI, means that the reported findings should be considered with caution.

### 7.5 Agreements and disagreements with other studies or reviews

As mentioned above, we excluded studies of interventions, policies, or programs that would not resemble a full‐scale GBI program. Other reviews tended to cast a wider net and included interventions, programs, and policies that were conditional on participation in specific activities, not paid regularly (e.g., lottery winnings) and not paid in fixed or predictable amounts (e.g., dividends from natural resources or casinos).

We included only primary research articles to obtain accurate and complete quantitative and statistical data for each eligible study. Other reviews included secondary sources, which we examined but found to be lacking in the level of quantitative detail necessary to conduct meta‐analyses.

In a synthesis of other reviews, Hasdell (2020) notes the differences between the features of interventions in empirical studies and those of an UBI and proposes that modeling studies may help to predict the effects of a UBI for specific populations and contexts. Most of the other reviews that we found also refer to the lack of empirical evidence on interventions that include all the features of UBI as evidence gaps. Our review focuses on GBI approaches as they have been tested in experiments and how the various types of GBI impact poverty‐related outcomes.

Yang (2021) refers to “nebulous” basic income definitions, which appear to be based on a mix of UBI and GBI criteria. We developed a typology based on empirical and conceptual variants of GBI (the latter also being implemented in new experiments), and we believe that this framework can be adapted to classify proposed variants of UBI also. This may help to clarify and to “name” the different types of proposals, so that discussions and debates revolve around the same concepts.

In terms of methodology, the only other review that assessed the quality of studies (risk of bias) and the certainty of the evidence across studies (GRADE) was by Wilson and McDaid (Wilson & McDaid, 2021). However, that review did not include a quantitative synthesis and only examined mental health outcomes. Chrisp (2022) assessed threats and strengths with respect to study design, conduct, and reporting, although this quality appraisal was done by experiment and not for each study or report. Pinto (2021) rated the quality of the included studies, but did not incorporate the ratings into the results, discussion, or conclusion sections of the review. Günther (2020) conducted a regression analysis of income and employment elasticities, while our review excluded employment outcomes.

Due to the methodological differences of the other reviews compared to this one, they reported the same basic findings as this review when examining the same studies, experiments, and outcomes, but without a systematic consideration of study quality or certainty of evidence (except as noted above). As well, without a framework to categorize the various types of GBI approaches, the findings were analyzed according to the study or experiment rather than by the type of intervention. As such, the other reviews generally concluded that there were mixed findings overall with some evidence of positive effects of the interventions. This review is more conservative in its conclusions, finding that implementing a full‐scale GBI program would be risky, given the existing body of evidence from GBI experiments.

## 8 AUTHORS' CONCLUSIONS

### 8.1 Implications for practice and policy

Conditional social assistance programs in high‐income countries, with stricter eligibility requirements implemented in recent decades, have not been successful in their goal of poverty reduction – based on indicators such as the escalation in food bank use (Food, 2022; Loh, 2020; Tarasuk, 2020) and the continued prevalence of food insecurity (Carrillo‐Álvarez, 2023; Loopstra, 2018; McKay, 2019). Income‐based measures also indicate that relative poverty rates are still between 8% and 15% in most developed countries (Eurostat, 2024; OECD, 2023a).

The body of research on GBI included in this review provides some evidence that unconditional income support has a beneficial effect on several poverty‐related outcomes. However, because the evidence is limited, a cautious approach is necessary.

Opponents of subsistence‐level GBI proposals argue that they would be unaffordable and may also be detrimental for some people because their GBI benefits may be lower than the total support they receive from various existing programs. It is also risky to generalize the findings of limited duration and dispersed RCT experiments that cannot provide information on long‐term or community‐level effects. As well, because of the complexities of and differences between the various GBI approaches that we identified, actual full‐scale programs may be implemented differently from the experimental GBI interventions. This potential departure was noted by researchers involved in the Seattle‐Denver experiment in the 1970s: “What ultimately comes out as welfare reform […] will not correspond specifically to any treatment that has appeared in any income maintenance [experiment] to date” (Bell, 1979, p. 49).

Based on the findings that we could compare across the reviewed experiments and studies, the strongest evidence of improvements in poverty‐related outcomes appears to be from interventions that provided a supplemental fixed‐amount type of GBI. Two of the experiments used this approach: the Finnish basic income experiment (Lassander, 2021; Simanainen, 2021) and the Stockton Economic Empowerment Demonstration (SEED, West, 2021), providing amounts of €560 and USD500, respectively, which were not affected by other income. Evidence of positive effects of small cash transfers on health and food insecurity also comes from research on tax credits for employed people with low incomes. For example, the Earned Income Tax Credit (EITC) in the US, which increased the income of eligible families by an average amount of $212 per month per family in 2023 (IRS, 2024), has been associated with improved birthweight, physical health, mental health, and food insecurity (Lenhart, 2023). A systematic review by Holdroyd (Holdroyd, 2023) also found that in‐work tax credits may have a protective effect against child maltreatment, especially in the reduction of child neglect. Thus, a larger transfer of $500 per month *per individual* in the form of an unconditional guaranteed income may have a larger impact on such poverty‐related outcomes.

Providing a supplemental GBI would help to address the gaps between existing programs that target specific populations and exclude some, such as single people without children. The supplemental approach could also lead to an eventual transition from conditional programs with intrusive, labor‐intensive monitoring, to a simpler income‐tested approach, if providing benefits with “no strings attached” is found to yield positive individual and community‐level results.

### 8.2 Implications for research

In light of the large number of GBI experiments that are now underway or will begin in the near future, we believe that the typology of GBI approaches presented in this review will be helpful for evaluating and synthesizing the findings of these experiments.

Another major challenge in synthesizing the results of past research is the large number of outcome variables that have been examined and reported. For research that is still in the planning phase or will be planned in the future, it would be very helpful for researchers to use standardized instruments for assessing the various dimensions of poverty, such as the HFSSM for food insecurity (USDA, 2022), the K10 for mental health (Kessler, 2002), and the updated Material Deprivation (MD) Index for economic hardship (Guio, 2017).

Providing financial assistance to people experiencing poverty affects every aspect of their lives, so it is understandable why past studies have collectively examined close to 200 outcome variables. Using validated instruments that are commonly used in the fields of health, sociology, and economics would help to yield findings that can be compared within studies as well as to population statistics, to provide absolute measures of the effects of GBI.

It may also be helpful to avoid investigations of general health, as poverty may only impact specific conditions or illnesses, so measures of overall state of health may not be sensitive enough to detect more nuanced effects. Gregory and Coleman‐Jensen (2017) found that low income was associated with increased rates of three of ten chronic illnesses (stroke, asthma, and chronic obstructive pulmonary disease), so it may be informative to examine possible impacts of GBI interventions on these conditions. Also, because of the psychological stresses associated with financial constraints, it would be important to examine whether GBI interventions can have a beneficial impact on any of the six leading causes of death which are related to stress: cancer, coronary heart disease, accidental injuries, respiratory disorders, cirrhosis of the liver and suicide (Salleh, 2008). Hospital separations for “accidents and injuries” were examined by Forget (2011/2013); however, this study looked only at community‐level effects in one arm of the Mincome experiment (i.e., the Dauphin “saturation” site).

Lastly, because of the disproportionate levels of poverty experienced by some groups, it may be helpful to examine the impacts of GBI interventions across PROGRESS‐Plus factors in a more comprehensive way than in previous studies. To assist researchers, guidance for reporting equity‐relevant randomized studies has been published (CONSORT‐Equity; Welch, 2017), while a guideline for reporting equity‐relevant observational studies is currently under development (STROBE‐Equity; Funnell, 2023).

## CONTRIBUTIONS OF AUTHORS

- Content: Anita Rizvi, Elizabeth Kristjansson, Leanne Idzerda, Christina M. Pollard.
- Systematic review methods: Vivian Welch, Elizabeth Kristjansson, George A. Wells, Alison Coates, Melissa K. Sharp, Olivia Magwood, Omar Dewidar, Julian Little, Alba Antequera, Jennifer Petkovic, Janet Jull, Elizabeth Ghogomu, Beverley J. Shea, Lawrence Mbuagbaw.
- Reference screening, data extraction, risk of bias assessments: Anita Rizvi, Madeleine M. Kearns, Michael B. Dignam, Alison Coates, Melissa K. Sharp, Olivia Magwood, Nour Elmestekawy, Sydney Rossiter, Ali Al‐Zubaidi, Omar Dewidar, Leanne Idzerda, Jean Marc Aguilera, Harshita Seal, Alba Antequera, Janet Jull, Lucas Gergyek, Elizabeth Ghogomu, Beverley J. Shea, Cristina M. Atance, Holly N. Ellingwood, Elizabeth Kristjansson.
- GRADE quality assessment: Anita Rizvi, Jennifer Petkovic.
- Information retrieval: Patrick R. Labelle.

## DECLARATIONS OF INTEREST

AR led this review as part of her doctoral thesis on poverty, food insecurity, and guaranteed basic income. BJS was the principal investigator in the development of the AMSTAR 2 guideline. OM co‐authored a series of reviews on interventions for people experiencing homelessness, two of which considered income interventions (Aubry, 2020; Moledina, 2021). VW is editor‐in‐chief of the Campbell Collaboration, and was acting CEO when this review was conducted. VW was not involved in the editorial decision process for this review. The other authors of this review declare no conflicts of interest.

### Preliminary timeframe

The approximate date for submission of the systematic review is December 2023.

### Plans for updating this review

We plan to update this review 4 years after publication. If this is not possible for some reason, the lead author will communicate this to the Social Welfare Coordinating Group.

Because of the nature of the interventions being examined (cash transfers to low‐income individuals), the confounding effects of pandemic relief measures, and the economic upheaval that ensued (including rapid price inflation), we propose a two‐stage approach to the analyses in the updated review, examining studies separately according to when they were started (e.g., before or after 2024). This approach may help ensure a more meaningful interpretation of the findings.

## SOURCES OF SUPPORT

### Internal sources

- No funding received.

### External sources

- No funding received.

## DIFFERENCES BETWEEN PROTOCOL AND REVIEW

### Study eligibility

During the pilot phase of the full‐text screening process, we refined our inclusion and exclusion criteria in two ways. For study design, we decided to exclude simulation and predictive modeling studies, and only include studies with analyses and findings based entirely on empirical data. Secondly, the question came up as to the minimum number of benefit payments that would qualify as “regular” according to our definition of GBI. Because we did not specify any limits in the protocol for the duration of follow‐up, we decided on three as the minimum number of payments that we could consider as regular.

### Supplementary searches

We added another strategy for hand searching gray literature: we searched Google Scholar for reviews (since 2017) of basic income studies, to scan their bibliographies for GBI studies we may have missed. This search also yielded two reviews (Chrisp, 2022; Somers, 2021) that were added to the list of other reviews in section 1.4 (Why it is important to do this review).

The protocol noted that the website of the government of Italy did not have an English search option. We found the same for the website of the government of France, so their website link was removed from the list of G7 government sites that were checked.

We stated in the protocol that we would contact the authors of the included studies, to ask if they knew of any other GBI studies that we hadn't already identified. However, because of the highly publicized nature of GBI experiments, spanning the areas of politics, economics, public health, social advocacy, among others, we were confident that we had identified all the eligible GBI experiments through academic databases and gray literature/Internet searches. Using these strategies, we reached a “saturation point,” after which we only came across the same studies that were already included. As well, due to our specific and restrictive inclusion criteria (more so than in other GBI reviews), we expected that we would receive many suggestions for studies and secondary sources that we had already excluded, and we believed this would have been an unproductive use of time for both the study authors and the review authors.

### Data extraction

We stated in the protocol that we would pilot the extraction form with ten articles, and we did not specify how many reviewers would conduct the pilot. Instead, five reviewers extracted data from two articles each, resulting in five articles done in duplicate. We believe that this yielded sufficient feedback to improve the extraction form.

### Excluded poverty‐related outcomes

We extracted all outcome data from the included studies; however, we did not include some of the outcomes in the synthesis of results because, while these were associated with poverty in general (e.g., number of parents in a family), it was not clear if or how changes in these outcomes impacted poverty for the participants in the experiments. We also excluded employment‐related outcomes (i.e., if participants worked more or less during the experiment) because it was not clear how transitioning between experimental GBI benefits and low‐paid work impacted on their experience of poverty. A complete list of outcomes, included and excluded, is provided in Supporting Information: Appendices 5 and 6.

### Subgroup analysis

We had planned to conduct subgroup analyses according to the study design (cluster randomized controlled trials [cRCTs], controlled before and after [CBA], etc.), study duration (<2 years, 2–4 years, >4 years), generosity of GBI benefits (relative to the official poverty line), individual/household level payment modality, poverty level threshold for eligibility (e.g., income below official poverty line, no income threshold), and take‐back rate if there is additional income from other sources. However, these analyses were not possible due to the diversity of outcome measures across different types of interventions.

### Reporting of the review findings

Due to the relevance of the review topic to societal equity, we had planned to follow the PRISMA‐Equity guideline extension (Welch, 2012). However, after examining the body of evidence from the included studies, it was clear that, even though some of the studies assessed the impacts of GBI interventions across factors such as gender, race and ethnicity, the interventions were not intended to address inequities, but rather, poverty in general. The PRISMA‐Equity guideline was intended for reviews of epidemiological studies addressing health inequities, which are due to systemic and structural barriers to healthcare. This review compared guaranteed basic income interventions to conditional social assistance (“usual care,” except in the case of supplemental GBI which did not replace existing programs). While there may be barriers to eligibility for marginalized populations with existing social assistance programs, this would require the examination of other studies which were not within the scope of this review.

## DATA AND ANALYSES

**Comparison 1**

GBI versus existing income supports: Analysis 1.1, 1.2, 1.3, 1.4, 1.5.

**Table:** 

Outcome or subgroup title	No. of studies	No. of participants	Statistical method	Effect size
1.1 Self‐reported food insecurity	2		Std. Mean Difference (IV, Random, 95% CI)	Subtotals only
1.2 Subjective financial well‐being	4		Std. Mean Difference (IV, Random, 95% CI)	Subtotals only
1.3 Self‐rated physical health	3		Std. Mean Difference (IV, Random, 95% CI)	Subtotals only
1.4 Self‐rated mental distress	2	1958	Std. Mean Difference (IV, Random, 95% CI)	−0.25 [−0.37, −0.13]
1.5 Self‐rated life satisfaction	3		Std. Mean Difference (IV, Random, 95% CI)	Subtotals only

**Analysis 1.1:**
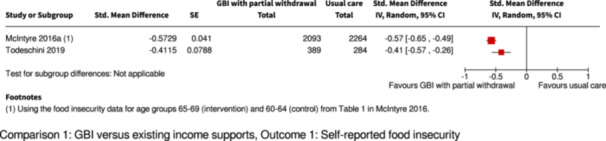
Comparison 1: GBI versus existing income supports, Outcome 1: Self‐reported food insecurity.

**Analysis 1.2:**
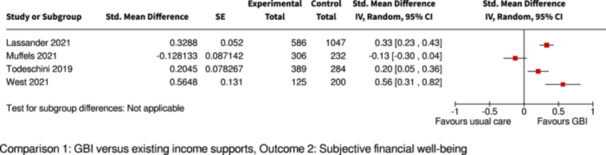
Comparison 1: GBI versus existing income supports, Outcome 2: Subjective financial well‐being.

**Analysis 1.3:**
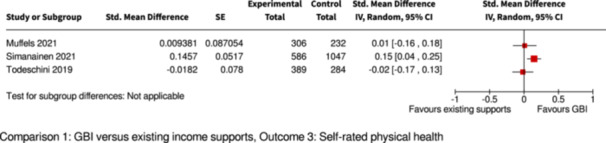
Comparison 1: GBI versus existing income supports, Outcome 3: Self‐rated physical health.

**Analysis 1.4:**
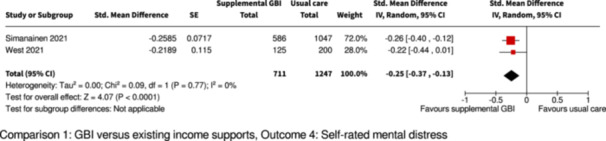
Comparison 1: GBI versus existing income supports, Outcome 4: Self‐rated mental distress.

**Analysis 1.5:**
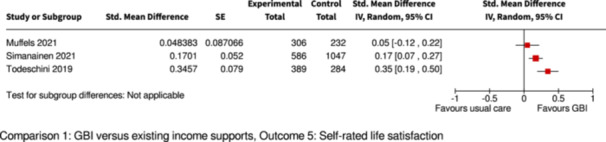
Comparison 1: GBI versus existing income supports, Outcome 5: Self‐rated life satisfaction.

## Supporting information

Supporting information.

Supporting information.

## References

[cl21414-bib-0002] Bonilla, F. , & Sekulova, F. (2019). *B‐Mincome's impact on life satisfaction*. The Institute of Environmental Science and Technology (Universitat Autonoma de Barcelona). https://ajuntament.barcelona.cat/dretssocials/sites/default/files/arxius-documents/bmincome_final_report_life_satisfaction.pdf

[cl21414-bib-0003] Calnitsky, D. , Latner, J. P. , & Forget, E. L. (2019). Life after work: The impact of basic income on nonemployment activities. Social Science History, 43(4), 657–677.

[cl21414-bib-0004] Calnitsky, D. , & Gonalons‐Pons, P. (2021). The impact of an experimental guaranteed income on crime and violence. Social Problems, 38(3), 778–798.

[cl21414-bib-0005] Elesh, D. , & Lefcowitz, M. J. (1977). The effects of the New Jersey‐Pennsylvania negative income tax experiment on health and health care utilization. Journal of Health and Social Behavior, 18(4), 391–405. 617642

[cl21414-bib-0006] Forget, E. L. (2013). New questions, new data, old interventions: The health effects of a guaranteed annual income. Preventive Medicine, 57(6), 925–928. 10.1016/j.ypmed.2013.05.029 23764242

[cl21414-bib-0007] Forget, E. L. (2011). The town with no poverty: The health effects of a canadian guaranteed annual income field experiment. Canadian Public Policy, 37(3), 283–305.

[cl21414-bib-0008] Gonalons‐Pons, P. , & Calnitsky, D. (2021). Exit, voice and loyalty in the family: Findings from a basic income experiment. Socio‐Economic Review, 20(3), 1395–1423.

[cl21414-bib-0009] Groeneveld, L. P. , Short, Jr., J. F. , & Thoits, P. (1979). Design of a study to assess the impact of income maintenance on delinquency. SRI International. https://www.ojp.gov/pdffiles1/Digitization/87569NCJRS.pdf

[cl21414-bib-0010] Kaluzny, R. L. (1979). Changes in the consumption of housing services: The Gary experiment. Journal of Human Resources, 14(4), 496–506.

[cl21414-bib-0011] Kehrer, B. H. , & Wolin, C. M. (1979). Impact of Income Maintenance on Low Birth Weight: Evidence from the Gary Experiment. The Journal of Human Resources, 14(4), 434–462. 575154

[cl21414-bib-0012] Kerachsky, S. H. (1977). Health and medical care utilization: A second approach. In H. W. Watts & A. Rees (Eds.), The New Jersey income‐maintenance experiment, volume 3: Expenditures, health, and social behavior; and the quality of the evidence (pp. 129–150). Academic Press.

[cl21414-bib-0013] Ladinsky, J. , & Wells, A. (1977). Social integration, leisure activity, media exposure, and lifestyle enhancement. In H. W. Watts & A. Rees (Eds.), The New Jersey income‐maintenance experiment, volume 3: Expenditures, health, and social behavior; and the quality of the evidence (pp. 195–223). Academic Press.

[cl21414-bib-0014] Lassander, M. , & Jauhiainen, S. (2021). Financial well‐being in basic income experiment. In O. Kangas , S. Jauhiainen , M. Simanainen , & M. Ylikanno (Eds.), Experimenting with unconditional basic income: Lessons from the Finnish BI experiment 2017–2018 (pp. 89–105). Edward Elgar Publishing Limited. 10.4337/9781839104855.00016

[cl21414-bib-0015] Mallar, C. D. (1977). The educational and labor‐supply responses of young adults in experimental families. In H. W. Watts & A. Rees (Eds.), The New Jersey income‐maintenance experiment, volume 2: Labor‐supply responses (pp. 163–184). Academic Press

[cl21414-bib-0016] Manheim, L. M. , & Minchella, M. E. (1979). Effects of income maintenance on school performance of children. In: Proceedings of the 1978 Conference on the Seattle and Denver Income Maintenance Experiments (pp. 531–549). State of Washington Departmentof Social & Health Services.

[cl21414-bib-0017] Maynard, R. A. (1977). The effects of the rural income maintenance experiment on the school performance of children. American Economic Review, 67(1), 370–375.

[cl21414-bib-0018] Maynard, R. A. , & Murnane, R. J. (1979). The effects of a negative income tax on school performance: Result of an experiment. Journal of Human Resources, 14(4), 463–476.

[cl21414-bib-0019] McDonald, J. F. , & Stephenson, S. P. J. (1979). The effect of income maintenance on the school‐enrollment and labor‐supply decisions of teenagers. Journal of Human Resources, 14(4), 488–495.

[cl21414-bib-0020] Mcintyre, L. , Dutton, D. J. , Kwok, C. , & Emery, J. C. H. (2016). Reduction of food insecurity among low‐income canadian seniors as a likely impact of a guaranteed annual income. Canadian Public Policy/Analyse de politiques, 42(3), 274–286.

[cl21414-bib-0021] Middleton, R. I. I. , & Allen, V. L. (1977). Social psychological effects. In H. W. Watts & A. Rees (Eds.), The New Jersey income‐maintenance experiment, volume 3: Expenditures, health, and social behavior; and the quality of the evidence (pp. 151–193). Academic Press.

[cl21414-bib-0022] Muffels, R. , Edzes, A. , Gramberg, P. , Rijnks Richard, H. , & Venhorst Viktor, A. (2021). Which regime works best in social welfare? Comparing Outcomes of eight Dutch RCT experiments. Web publication/site, Technequality. https://technequalityproject.eu/files/d41fddutchubiexperimentsv1020-04-2021pdf

[cl21414-bib-0023] Nicholson, W. (1977). Expenditure patterns: A descriptive survey. In H. W. Watts & A. Rees (Eds.), The New Jersey income‐maintenance experiment, volume 3: Expenditures, health, and social behavior; and the quality of the evidence (pp. 15–43). Academic Press.

[cl21414-bib-0024] O'Connor, J. F. , & Madden, J. P. (1979). The negative income tax and the quality of dietary intake. The Journal of Human Resources, 14(4), 507–517. 528785

[cl21414-bib-0025] Simanainen, M. , & Tuulio‐Henriksson, A. (2021). Subjective health, well‐being andcognitive capabilities. In O. Kangas , S. Jauhiainen , M. Simanainen , & M. Ylikanno (Eds.), Experimenting with unconditional basic income: Lessons from the Finnish BI experiment 2017–2018 (pp. 71–88). Edward Elgar Publishing Limited. 10.4337/9781839104855.00015

[cl21414-bib-0026] Thoits, P. , & Hannan, M. (1979). Income and psychological distress: The impact of an income‐maintenance experiment. Journal of Health and Social Behavior, 20(2), 120–138. 479525

[cl21414-bib-0027] Todeschini, F. , & Sabes‐Figuera, R. (2019). Barcelona city council welfare programme: Impact evaluation results. Ivàlua – Institut Català d'Avaluació de Polítiques Públiques (Catalan Institute of Public Policy Evaluation).

[cl21414-bib-0028] Venti, S. F. (1984). The effects of income maintenance on work, schooling, and non‐market activities of youth. Review of Economics and Statistics, 66(1), 16–25.

[cl21414-bib-0029] West, S. , Castro Baker, A. , Samra, S. , & Coltrera, E. (2021). *SEED: Stockton economic empowerment demonstration: Preliminary analysis: SEED's first year*. Stockton Economic Empowerment Demonstration. https://www.stocktondemonstration.org/s/SEED_Preliminary-Analysis-SEEDs-First-Year_Final-Report_Individual-Pages.pdf

[cl21414-bib-0031] Calnitsky, D. , & Latner, J. P. (2017). Basic income in a small town: Understanding the elusive effects on work. Social Problems, 64(3), 373–397.

[cl21414-bib-0032] Choudhry, S. A. , & Hum, D. J. (1995). Graduated work incentives and how they affect marital stability: The Canadian evidence. Applied Economics Letters, 2(10), 367.

[cl21414-bib-0033] Choudhry, S. A. , & Arvin, B. M. (2001). Negative income taxes and household transition dynamics: Evidence from the Canadian MINCOME experiment. International Journal of Applied Economics and Econometrics, 9(3), 255–284.

[cl21414-bib-0034] Cogan, J. F. (1983). Labor supply and negative income taxation – New evidence from the New‐Jersey–Pennsylvania experiment. Economic Inquiry, 21(4), 465–484.

[cl21414-bib-0035] Greenberg, D. , & Halsey, H. (1983). Systematic misreporting and effects of income‐maintenance experiments on work effort – Evidence from the Seattle‐Denver experiment. Journal of Labor Economics, 1(4), 380–407.

[cl21414-bib-0036] Groeneveld, L. P. , Tuma, N. B. , & Hannan, M. T. (1980). The effects of negative income‐tax programs on marital dissolution. Journal of Human Resources, 15(4), 654–674.

[cl21414-bib-0037] Hannan, M. T. , Tuma, N. B. , & Groeneveld, L. P. (1977). Income and marital events – Evidence from an income‐maintenance experiment. American Journal of Sociology, 82(6), 1186–1211.

[cl21414-bib-0038] Heffernan, J. (1977). Impact of a negative income tax on awareness of social services. Social Work Research and Abstracts, 13(2), 17–23.

[cl21414-bib-0039] Hum, D. P. , & Choudhry, S. (1992). Income, work and marital dissolution: Canadian experimental evidence. Journal of Comparative Family Studies, 23(2), 249–265.

[cl21414-bib-0040] Keeley, M. C. (1980). The effect of a negative income tax on migration. Journal of Human Resources, 15(4), 695–706.

[cl21414-bib-0041] Keeley, M. C. (1980). The effects of negative income‐tax programs on fertility. Journal of Human Resources, 15(4), 675–694.

[cl21414-bib-0042] Keeley, M. C. (1987). The effects of experimental negative income‐tax programs on marital dissolution – Evidence from the Seattle and Denver income‐maintenance experiments. International Economic Review, 28(1), 241–257.

[cl21414-bib-0043] McDowell, T. , & Ferdosi, M. (2020). The experiences of social assistance recipients on the Ontario basic income pilot. Canadian Review of Sociology = Revue canadienne de sociologie, 57(4), 681–707. 33151642 10.1111/cars.12306

[cl21414-bib-0044] McDowell, T. , & Ferdosi, M. (2021). The impacts of the Ontario basic income pilot: A comparative analysis of the findings from the Hamilton region. Basic Income Studies, 16(2), 209–256.

[cl21414-bib-0045] McIntyre, L. , Kwok, C. , Emery, J. C. H. , & Dutton Daniel, J. (2016). Impact of a guaranteed annual income program on Canadian seniors' physical, mental and functional health. Canadian Journal of Public Health = Revue canadienne de sante publique, 107(2), e176–e182. 27526215 10.17269/cjph.107.5372 PMC6972215

[cl21414-bib-0046] Pencavel, J. H. (1982). Unemployment and the labor supply effects of the Seattle‐Denver income maintenance experiments. Research in Labor Economics, 5, 1–31.

[cl21414-bib-0047] Robins, P. K. , Tuma, N. B. , & Yaeger, K. E. (1980). Effects of sime/dime on changes in employment status. Journal of Human Resources, 15(4), 545–573.

[cl21414-bib-0048] West, R. W. (1980). The effects on the labor supply of young nonheads. Journal of Human Resources, 15(4), 574–590.

[cl21414-bib-0050] Alston, P. (2017). Universal basic income as a social rights‐based antidote to growing economic insecurity. *Social Science Research Network*. NYU School of Law, Public Law Research Paper No. 17–51. https://papers.ssrn.com/abstract=3079907

[cl21414-bib-0051] Andrade, C. (2019). The P value and statistical significance: Misunderstandings, explanations, challenges, and alternatives. Indian Journal of Psychological Medicine, 41(3), 210–215. 10.4103/IJPSYM.IJPSYM_193_19 31142921 PMC6532382

[cl21414-bib-0052] Aubry, T. , Bloch, G. , Brcic, V. , Saad, A. , Magwood, O. , Abdalla, T. , Alkhateeb, Q. , Xie, E. , Mathew, C. , Hannigan, T. , Costello, C. , Thavorn, K. , Stergiopoulos, V. , Tugwell, P. , & Pottie, K. (2020). Effectiveness of permanent supportive housing and income assistance interventions for homeless individuals in high‐income countries: A systematic review. The Lancet Public Health, 5(6), e342–e360. 32504587 10.1016/S2468-2667(20)30055-4

[cl21414-bib-0053] Barder, O. M. (2009). What is poverty reduction? Center for Global Development Working Paper, *23*(170), 203–239. https://citeseerx.ist.psu.edu/viewdoc/download?doi=10.1.1.393.7930&rep=rep1&type=pdf

[cl21414-bib-0054] Bawden, D. L. , Harrar William, S. , & Kerachsky, S . (1976). *The rural income maintenance experiment* (Vol. 5). Institute for Research on Poverty, University of Wisconsin.

[cl21414-bib-0055] Bell, J. G. , Lines, P. M. , & Linn, M. (Eds.). Proceedings of the 1978 conference on the Seattle and Denver income maintenance experiments. https://www.dshs.wa.gov/sites/default/files/rda/reports/research-6-01.pdf

[cl21414-bib-0056] Bidadanure, J. , Kline, S. , Moore, C. , Rainwater, B. , & Thomas, C . (2018). Basic income in cities: A guide to city experiments and pilot projects. National League of Cities. https://www.nlc.org/wp-content/uploads/2020/10/BasicIncomeInCities_Report_For-Release-.pdf

[cl21414-bib-0057] Bidadanure, J. U. (2019). The political theory of universal basic income. Annual Review of Political Science, 22(1), 481–501.

[cl21414-bib-0058] Basic Income Earth Network . (2020). About basic income. BIEN – Basic Income Earth Network. https://basicincome.org/about-basic-income/

[cl21414-bib-0059] Boozary, A. S. , & Shojania, K. G. (2018). Pathology of poverty: The need for quality improvement efforts to address social determinants of health. BMJ Quality & Safety, 27(6), 421–424. 10.1136/bmjqs-2017-007552 29511090

[cl21414-bib-0060] Borenstein, M. (2022). In a meta‐analysis, the I‐squared statistic does not tell us how much the effect size varies. Journal of Clinical Epidemiology, 152, 281–284. 10.1016/j.jclinepi.2022.10.003 36223816

[cl21414-bib-0061] Borenstein, M. , & Hedges, L. V. (2019). Effect sizes for meta‐analysis. In H. Cooper , L. V. Hedges , & J. C. Valentine (Eds.), The handbook of research synthesis and meta‐analysis (3rd ed., pp. 208–242). Russell Sage Foundation.

[cl21414-bib-0062] Calloway, E. E. , Carpenter, L. R. , Gargano, T. , Sharp, J. L. , & Yaroch, A. L. (2023). New measures to assess the “Other” three pillars of food security – Availability, utilization, and stability. International Journal of Behavioral Nutrition and Physical Activity, 20(1), 51. 10.1186/s12966-023-01451-z 37101157 PMC10134599

[cl21414-bib-0063] Caputo, R. K. (2007). Working and poor: A panel study of maturing adults in the U.S. Families in Society, 88(3), 351–359.

[cl21414-bib-0064] Carey, M. , & Bell, S. (2020). Universal credit, lone mothers and poverty: Some context and challenges for social work with children and families. Critical and Radical Social Work, 8(2), 189–203.

[cl21414-bib-0065] Carrillo‐Álvarez, E. (2023). Perspective: Food and nutrition insecurity in Europe: Challenges and opportunities for dietitians. Advances in Nutrition, 14(5), 995–1004. 10.1016/j.advnut.2023.07.008 37543145 PMC10509433

[cl21414-bib-0066] Carson, J. , & Boege, S. (2020). The intersection of food availability. Access, & Affordability with Food Security and Health.

[cl21414-bib-0067] Causa, O. , Soldani, E. , Luu, N. , & Soriolo, C . (2022). A cost‐of‐living squeeze? Distributional implications of rising inflation.

[cl21414-bib-0068] Centre for Social Justice . (2018). Universal basic income: An effective policy for poverty reduction? Centre for Social Justice. https://www.centreforsocialjustice.org.uk/wp-content/uploads/2018/08/CSJ_UBI_August-2018.pdf

[cl21414-bib-0069] Chrisp, J. , Smyth, L. , Stansfield, C. , Pearce, N. , France, R. , & Taylor, C. (2022). Basic income experiments in OECD countries: A rapid evidence review. EPPI Centre, UCL Social Research Institute, University College London. https://eppi.ioe.ac.uk/cms/Default.aspx?tabid=3856

[cl21414-bib-0070] Coady, D. , Jahan, S. , Shang, B. , & Matsumoto, R. (2021). Guaranteed minimum income schemes in Europe: Landscape and design. IMF Working Paper WP/21/179. 10.5089/9781513584379.001.A001

[cl21414-bib-0071] Cutillo, A. , Raitano, M. , & Siciliani, I. (2020). Income‐based and consumption‐based measurement of absolute poverty: Insights from Italy. Social Indicators Research, 161, 689–710.

[cl21414-bib-0072] Day, C. (2017). The Beveridge report and the foundations of the welfare state. The National Archives blog.

[cl21414-bib-0073] Deeks, J. J. , Higgins, J. P. T. , & Altman, D. G. (Eds.). (2022). Chapter 10: Analysing data and undertaking meta‐analyses [Chapter 10]. Cochrane Handbook for Systematic Reviews of Interventions version 6.3. Cochrane. https://training.cochrane.org/handbook/current/chapter-10

[cl21414-bib-0074] Economic Security Project . (2022). Champions for guaranteed income celebrate the announcement of the 100th guaranteed income pilot program in five years. Economic Security Project.

[cl21414-bib-0075] Eldridge, S. , Campbell, M. , Campbell, M. , Drahota‐Towns, A. , Giraudeau, B. , Higgins, J. , Reeves, B. , & Siegfried, N . (2016). Revised Cochrane risk of bias tool for randomized trials (RoB 2.0): Additional considerations for cluster‐randomized trials. riskofbias.info.

[cl21414-bib-0076] Elsevier . (2022). Research data storage and retention. Elsevier Author Services – Articles.

[cl21414-bib-0077] Encyclopædia Britannica . (2024). Poverty | Definition, causes, types, & facts | Britannica.

[cl21414-bib-0078] Economic Policy Institute . (2021). The productivity–pay gap. Economic Policy Institute.

[cl21414-bib-0079] Eurostat . (2012). Measuring material deprivation in the EU — Indicators for the whole population and child‐specific indicators. Eurostat.

[cl21414-bib-0080] Eurostat . (2023). Glossary: Household budget survey (HBS) [Glossary]. Eurostat – Statistics Explained.

[cl21414-bib-0081] Eurostat . (2024). Statistics | Eurostat.

[cl21414-bib-0082] Food and Agriculture Organization of the United Nations . (2018). Food Insecurity Experience Scale | Voices of the Hungry.

[cl21414-bib-0083] FAO . (2023). *The impact of disasters on agriculture and food security 2023: Avoiding and reducing losses through investment in resilience*. FAO.

[cl21414-bib-0084] Food Banks Canada . (2022). HungerCount 2022.

[cl21414-bib-0085] Food Banks Canada . (2019). HungerCount 2019. Food Banks Canada. https://www.foodbankscanada.ca/getmedia/496dff0c-fe21-46b5-bc71-bd4c20210e6a/HungerCount-2019_FINAL.pdf.aspx?ext=.pdf

[cl21414-bib-0086] Forget, E. L. (2011). The town with no poverty: The health effects of a Canadian guaranteed annual income field experiment. Canadian Public Policy, 37(3), 283–305. 10.3138/cpp.37.3.283

[cl21414-bib-0087] Funnell, S. , Jull, J. , Mbuagbaw, L. , Welch, V. , Dewidar, O. , Wang, X. , Lesperance, M. , Ghogomu, E. , Rizvi, A. , Akl Elie, A. , Avey Marc, T. , Antequera, A. , Bhutta Zulfiqar, A. , Chamberlain, C. , Craig, P. , Cuervo Luis, G. , Dicko, A. , Ellingwood, H. , Feng, C. , … Young, T. (2023). Improving social justice in observational studies: Protocol for the development of a global and Indigenous STROBE‐equity reporting guideline. International Journal for Equity in Health, 22(1), 55. 10.1186/s12939-023-01854-1 36991403 PMC10060140

[cl21414-bib-0088] Gentilini, U. , Grosh, M. , Rigolini, J. , & Yemtsov, R. (2020). Exploring universal basic income: A guide to navigating concepts, evidence, and practices. World Bank.

[cl21414-bib-0089] Gibson, M. , Hearty, W. , & Craig, P. (2020). The public health effects of interventions similar to basic income: A scoping review. The Lancet Public Health, 5(3), e165–e176. 32113520 10.1016/S2468-2667(20)30005-0 PMC7208547

[cl21414-bib-0090] Government of Ireland . (2022). Ireland's basic income for the arts pilot scheme launched by government. https://www.gov.ie/en/press-release/27aed-irelands-basic-income-for-the-arts-pilot-scheme-launched-by-government/#

[cl21414-bib-0091] Government of Ontario . (2022). Working and earning while on Ontario Works. http://www.ontario.ca/page/working-and-earning-while-ontario-works

[cl21414-bib-0092] Green, D. A. (2022). A reanalysis of “The Town with No Poverty: The Health Effects of a Canadian Guaranteed Annual Income Field Experiment”. Canadian Public Policy, 48(4), 539–548. 10.3138/cpp.2021-025

[cl21414-bib-0093] Gregory, C. A. , & Coleman‐Jensen, A. (2017). Food insecurity, chronic disease, and health among working‐age adults. United States Department of Agriculture.

[cl21414-bib-0094] Guio, A.‐C. , Marlier, E. , Gordon, D. , Fahmy, E. , Nandy, S. , & Pomati, M. (2016). Improving the measurement of material deprivation at the European Union level. Journal of European Social Policy, 26(3), 219–333.

[cl21414-bib-0095] Guio, A.‐C. , Gordon, D. , Najera, H. , & Pomati, M. (2017). Revising the EU material deprivation variables (Vol. 10, p. 33408). European Union.

[cl21414-bib-0096] Gundersen, C. , & Ziliak James, P. (2015). Food insecurity and health outcomes. Health Affairs, 34(11), 1830–1839. 26526240 10.1377/hlthaff.2015.0645

[cl21414-bib-0097] Gupta, R. , Jacob, J. , & Bansal, G. (2021). The role of UBI in mitigating the effects of psychosocial stressors: A review and proposal. Psychological Reports, 125(4), 00332941211005115. 10.1177/00332941211005115 33789535

[cl21414-bib-0098] Gupta, B. S. , & Theoharis, L. (2020). US poverty measure is misleading, leaves out millions of americans who need help. Business Insider.

[cl21414-bib-0099] Guyatt, G. H. , Oxman Andrew, D. , Kunz, R. , Woodcock, J. , Brozek, J. , Helfand, M. , Alonso‐Coello, P. , Glasziou, P. , Jaeschke, R. , Akl Elie, A. , Norris, S. , Vist, G. , Dahm, P. , Shukla Vijay, K. , Higgins, J. , Falck‐Ytter, Y. , & Schünemann Holger, J. (2011). GRADE guidelines: 7. Rating the quality of evidence—inconsistency [GRADE guidelines]. Journal of Clinical Epidemiology, 64(12), 1294–1302. 10.1016/j.jclinepi.2011.03.017 21803546

[cl21414-bib-0100] Günther, S. (2020). Basic income: A systematic review of existing programs and their effects. Umeå School of Business and Economics, Umeå University.

[cl21414-bib-0101] Hasdell, R. (2020). What we know about universal basic income: A cross‐synthesis of reviews. Basic Income Lab.

[cl21414-bib-0102] Hedges, L. V. (2007). Effect sizes in cluster‐randomized designs. Journal of Educational and Behavioral Statistics, 32(4), 341–370.

[cl21414-bib-0103] Higgins, J. P. T. , Thompson, S. G. , Deeks, J. J. , & Altman, D. G. (2003). Measuring inconsistency in meta‐analyses. BMJ, 327(7414), 557–560. 10.1136/bmj.327.7414.557 12958120 PMC192859

[cl21414-bib-0104] Higgins, J. P. T. , Sterne, J. A. C. , Savovic, J. , Page, M. J. , Hróbjartsson, A. , Boutron, I. , Reeves, B. , & Eldridge, S. (2016). A revised tool for assessing risk of bias in randomized trials. Cochrane Database of Systematic Reviews, 10(Suppl. 1), 29–31.

[cl21414-bib-0105] Holdroyd, I. , Barton, G. , & Holdroyd, D. (2023). The effect of working tax credits on child maltreatment rates: A systematic review. Child Abuse & Neglect, 143, 106279. 10.1016/j.chiabu.2023.106279 37331186

[cl21414-bib-0106] Hombrados, J. G. , & Waddington, H. (2012). A tool to assess risk of bias for experiments and quasi‐experiments in development research. International Initiative for Impact Evaluation (3ie).

[cl21414-bib-0107] Horowitz, J. , Igielnik, R. , & Kochhar, R. (2020). Trends in income and wealth inequality. Pew Research Center's Social & Demographic Trends Project.

[cl21414-bib-0108] Hoynes, H. , & Rothstein, J. (2019). Universal basic income in the United States and advanced countries. Annual Review of Economics, 11(1), 929–958.

[cl21414-bib-0109] Hum, D. P. J. , Laub, M. E. , & Powell, B. J. (1979). The objectives and design of the Manitoba basic annual income experiment (Mincome Technical Report 1). 10.5203/FK2/XAGGJT

[cl21414-bib-0110] Hum, D. P. J. , Metcalf, C. E. , Laub, M. E. , & Sabourin, D . (1979). The Sample Design and Assignment Model (MINCOME Technical Report 2). 10.5203/FK2/VPZILS

[cl21414-bib-0111] Hunger + Health . (2022). What Is Food Insecurity in America? Hunger and Health.

[cl21414-bib-0112] Hämäläinen, K. , Kanninen, O. , & Verho, J . (2022). Finnish basic income experiment. AEA RCT Registry. https://www.socialscienceregistry.org/trials/2095

[cl21414-bib-0113] International Labour Organization . (2016). World Employment and Social Outlook 2016 – Transforming jobs to end poverty (Vol. 192). International Labour Organization. http://www.ilo.org/wcmsp5/groups/public/@dgreports/@dcomm/@publ/documents/publication/wcms_481534.pdf

[cl21414-bib-0114] International Monetary Fund . (2022). Statistical appendix. In J. Procopio , L. Scott Morales , J. Unwin , M. Harrup , N. Morrison , & H. Medina (Eds.), World economic outlook: War sets back the global recovery (pp. 101–127). International Monetary Fund.

[cl21414-bib-0115] Institute for Research on Poverty . (2017). How is poverty measured? Institute for Research on Poverty. https://www.irp.wisc.edu/resources/how-is-poverty-measured/

[cl21414-bib-0116] IRS . (2024). Statistics for tax returns with the Earned Income Tax Credit (EITC) | Earned Income Tax Credit.

[cl21414-bib-0117] Interagency Technical Working Group . (2021). Final report of the interagency technical working group on evaluating alternative measures of poverty. U.S. Bureau of Labor Statistics. https://www.bls.gov/evaluation/final-report-of-the-interagency-technical-working-group-on-evaluating-alternative-measures-of-poverty.pdf

[cl21414-bib-0118] Jenkins, B. (2019). A guaranteed basic income and the aesthetics of existence. Journal of Cultural Economy, 12(1), 21–35.

[cl21414-bib-0119] Jimenez, E. , Waddington, H. , Goel, N. , Prost, A. , Pullin, A. , White, H. , Lahiri, S. , & Narain, A. (2018). Mixing and matching: Using qualitative methods to improve quantitative impact evaluations (IEs) and systematic reviews (SRs) of development outcomes. Journal of Development Effectiveness, 10(4), 400–421.

[cl21414-bib-0120] Jüni, P. , Altman, D. G. , & Egger, M. (2001). Assessing the quality of controlled clinical trials. BMJ: British Medical Journal, 323(7303), 42–46. 11440947 10.1136/bmj.323.7303.42 PMC1120670

[cl21414-bib-0121] Kangas, O. , Jauhiainen, S. , Simanainen, M. & Ylikännö, M. , (Eds). (2021). Experimenting with unconditional basic income: Lessons from the Finnish BI experiment 2017–2018. Edward Elgar Publishing. https://www.elgaronline.com/display/edcoll/9781839104848/9781839104848.xml

[cl21414-bib-0122] Kavanagh, J. , Oliver, S. , Caird, J. , Tucker, H. , Greaves, A. , Oakley, A. , Harden, A. , Lorenc, T. , & Thomas, J. (2009). Inequalities and the mental health of young people: A systematic review of secondary school‐based cognitive behavioural interventions. EPPI‐Centre, Social Science Research Unit, Institute of Education, University of London.

[cl21414-bib-0123] Kehrer, K. C. , McDonald, J. F. , & Moffitt, R. A. (1979). Final report of the gary income maintenance experiment: Labor supply.

[cl21414-bib-0124] Kenworthy, L. (1999). Do social‐welfare policies reduce poverty? A cross‐national assessment. Social Forces, 77(3), 1119–1139.

[cl21414-bib-0125] Kershaw, D. , & Fair, J . (1976). *The New Jersey income‐maintenance experiment Volume 2: Labor-supply responses*. Academic Press.

[cl21414-bib-0126] Kershaw, D. , & Fair, J . (1976). *The New Jersey income‐maintenance experiment, Volume 3: Expenditures, health, and social behavior; and the quality of the evidence*. Academic Press.

[cl21414-bib-0127] Kessler, R. C. , Andrews, G. , Colpe, L. J. , Hiripi, E. , Mroczek, D. K. , Normand, S.‐L. T. , Walters, E. E. , & Zaslavsky, A. M. (2002). Short screening scales to monitor population prevalences and trends in non‐specific psychological distress. Psychological Medicine, 32(6), 959–976. 10.1017/S0033291702006074 12214795

[cl21414-bib-0128] Koebel, K. , & Pohler, D. (2019). Expanding the Canada workers benefit to design a guaranteed basic income. Canadian Public Policy, 45(3), 283–309.

[cl21414-bib-0129] Konle‐Seidl, R. A. (2021). Strengthening minimum income protection in the EU. Publications Office of the EU.

[cl21414-bib-0130] Krieger, N. , Williams, D. R. , & Moss, N. E. (1997). Measuring social class in US public health research: Concepts, methodologies, and guidelines. Annual Review of Public Health, 18(1), 341–378. 10.1146/annurev.publhealth.18.1.341 9143723

[cl21414-bib-0131] Laín, B. (2019). Report on the preliminary results of the B‐MINCOME project (2017–2018): Combining a guaranteed minimum income and active social policies in deprived urban areas of Barcelona. Planning and Innovation Department Area of Social Rights, Barcelona City Council.

[cl21414-bib-0132] Laín, B. , & Julià, A. (2022). Why do poor people not take up benefits? Evidence from the Barcelona's B‐MINCOME experiment. Journal of Social Policy, 53(1), 1–22. 10.1017/S0047279422000575

[cl21414-bib-0133] Lenhart, O. (2023). The earned income tax credit and food insecurity. American Journal of Agricultural Economics, 105(5), 1543–1570. 10.1111/ajae.12365

[cl21414-bib-0134] Loh, S. , Knight, A. , & Loopstra, R. (2020). Working‐age adults using food banks in England have significantly poorer health and higher rates of mental health conditions than adults in the general population: A cross‐sectional quantitative study. Health & Social Care in the Community, 1–12. 10.1111/hsc.13226 33211358

[cl21414-bib-0135] Long, R. B. (1972). Income maintenance experiments.

[cl21414-bib-0136] Loopstra, R. (2018). Interventions to address household food insecurity in high‐income countries. Proceedings of the Nutrition Society, 77(3), 270–281. 29580316 10.1017/S002966511800006X

[cl21414-bib-0137] Loopstra, R. , & Tarasuk, V. (2013). What does increasing severity of food insecurity indicate for food insecure families? Relationships between severity of food insecurity and indicators of material hardship and constrained food purchasing. Journal of Hunger & Environmental Nutrition, 8(3), 337–349.

[cl21414-bib-0138] Martin‐West, S. , Baker Amy, C. , Balakrishnan, S. , Rao, K. , & Tan Guan, Y . (2019). Pre‐analysis plan Stockton economic empowerment demonstration.

[cl21414-bib-0139] Martinelli, L . (2017). IPR policy brief: Assessing the case for a universal basic income in the UK.

[cl21414-bib-0140] McDowell, T. , & Ferdosi, M. (2021). The impacts of the Ontario basic income pilot: A comparative analysis of the findings from the Hamilton region. Basic Income Studies, 16(2), 209–256.

[cl21414-bib-0141] McGowan, J. , Sampson, M. , Salzwedel, D. M. , Cogo, E. , Foerster, V. , & Lefebvre, C. (2016). PRESS peer review of electronic search strategies: 2015 guideline statement. Journal of Clinical Epidemiology, 75, 40–46. 10.1016/j.jclinepi.2016.01.021 27005575

[cl21414-bib-0142] McKay, F. H. , Haines, B. C. , & Dunn, M. (2019). Measuring and understanding food insecurity in Australia: A systematic review. International Journal of Environmental Research and Public Health, 16(3), 476. 10.3390/ijerph16030476 30736305 PMC6388276

[cl21414-bib-0143] McLeod, L. , & Veall, M. (2006). The dynamics of food insecurity and overall health: Evidence from the Canadian National Population Health Survey. Applied Economics, 38(18), 2131–2146.

[cl21414-bib-0144] Mein, G . (2023). Was würdest du tun, wenn du plötzlich Grundeinkommen hättest? Mein Grundeinkommen. https://www.mein-grundeinkommen.de/

[cl21414-bib-0145] Merriam‐Webster . (2024). Definition of POVERTY.

[cl21414-bib-0146] Merrill, R. , Neves, C. , & Laín, B. (2022). How the findings help advance the basic income debate and advocacy. In R. Merrill , C. Neves , & B. Laín (Eds.), Basic income experiments: A critical examination of their goals, contexts, and methods (pp. 173–205). Springer International Publishing.

[cl21414-bib-0147] The Methods Group of the Campbell Collaboration . (2019). Methodological expectations of Campbell Collaboration intervention reviews: Conduct standards. Campbell Policies and Guidelines.

[cl21414-bib-0148] The Methods Group of the Campbell Collaboration . (2019). Methodological expectations of Campbell Collaboration intervention reviews: Reporting standards. Campbell Policies and Guidelines.

[cl21414-bib-0149] Meyer, B. D. , & Sullivan, J. X. (2012). Identifying the disadvantaged: Official poverty, consumption poverty, and the new supplemental poverty measure. Journal of Economic Perspectives, 26(3), 111–136.

[cl21414-bib-0150] Microsoft Corporation . (2022). Microsoft Excel spreadsheet software – Microsoft 365. Microsoft.

[cl21414-bib-0151] Moher, D. , Liberati, A. , Tetzlaff, J. , & Altman Douglas, G. (2009). Preferred reporting items for systematic reviews and meta‐analyses: The PRISMA statement. BMJ, 339, b2535. 10.1136/bmj.b2535 19622551 PMC2714657

[cl21414-bib-0152] Moledina, A. , Magwood, O. , Agbata, E. , Hung, J.‐H. , Saad, A. , Thavorn, K. , Salvalaggio, G. , Bloch, G. , Ponka, D. , Aubry, T. , Kendall, C. , & Pottie, K. (2021). A comprehensive review of prioritised interventions to improve the health and wellbeing of persons with lived experience of homelessness. Campbell Systematic Reviews, 17(2), e1154. 10.1002/cl2.1154 37131928 PMC8356292

[cl21414-bib-0153] Muffels, R. , & Gielens, E. (2019). Job search, employment capabilities and well‐being of people on welfare in the Dutch ‘Participation Income’ experiments. In L. Delsen (Ed.), Empirical research on an unconditional basic income in Europe (pp. 109–138). Springer International Publishing.

[cl21414-bib-0154] Munnell, A. H. (Ed.). (1986). Lessons from the income maintenance experiments: An overview. Federal Reserve Bank of Boston Conference. https://core.ac.uk/download/pdf/6706962.pdf

[cl21414-bib-0155] National Center for Education Statistics (NCES) . (2017). Digest of Education Statistics, 2017. IES NCES National Center for Education Statistics. https://nces.ed.gov/programs/digest/d17/tables/dt17_219.75.asp

[cl21414-bib-0156] National Center for Education Statistics (NCES) . (2019). Digest of education statistics, 2019. https://nces.ed.gov/programs/digest/d19/tables/dt19_219.70.asp

[cl21414-bib-0157] National Center for Education Statistics (NCES) . (2021). Digest of education statistics, 2021. IES NCES National Center for Education Statistics. https://nces.ed.gov/programs/digest/d21/

[cl21414-bib-0158] Nettle, D. , Johnson, E. , Johnson, M. , & Saxe, R. (2021). Why has the COVID‐19 pandemic increased support for Universal Basic Income? Humanities and Social Sciences Communications, 8(1), 1–12.38617731

[cl21414-bib-0159] Nilsson Sandra, F. , Nordentoft, M. , & Hjorthøj, C. (2019). Individual‐level predictors for becoming homeless and exiting homelessness: A systematic review and meta‐analysis. Journal of Urban Health, 96(5), 741–750. 31388823 10.1007/s11524-019-00377-x PMC6814700

[cl21414-bib-0160] O'Neill, J. , Tabish, H. , Welch, V. , Petticrew, M. , Pottie, K. , Clarke, M. , Evans, T. , Pardo Pardo, J. , Waters, E. , White, H. , & Tugwell, P. (2014). Applying an equity lens to interventions: Using PROGRESS ensures consideration of socially stratifying factors to illuminate inequities in health. Journal of Clinical Epidemiology, 67, 56–64. 24189091 10.1016/j.jclinepi.2013.08.005

[cl21414-bib-0161] Organisation for Economic Co‐operation and Development . (2019). A declining middle‐income class? Under Pressure: The Squeezed Middle Class. OECD.

[cl21414-bib-0162] Organisation for Economic Co‐operation and Development . (2020). Social Expenditure Database (SOCX) – OECD. OECD – Better Policies for Better Lives.

[cl21414-bib-0163] Organisation for Economic Co‐operation and Development . (2021). Perspectives on global development 2021: From protest to progress? OECD.

[cl21414-bib-0164] Organisation for Economic Co‐operation and Development . (2022). Housing conditions – OECD. https://www.oecd.org/housing/data/affordable-housing-database/housing-conditions.htm

[cl21414-bib-0165] Organisation for Economic Co‐operation and Development . (2023). Poverty rate (indicator). OECD. http://data.oecd.org/inequality/poverty-rate.htm

[cl21414-bib-0166] OECD . (2023). Government at a glance. OECD.

[cl21414-bib-0167] Orrell, B. (2021). Let them spend cash? American Enterprise Institute – AEI.

[cl21414-bib-0168] Parker, R. A. , & Berman, N. G. (Eds.) (2016). Cohort studies. In Planning clinical research (pp. 82–94). Cambridge University Press.

[cl21414-bib-0169] Parolin, Z. (2021). Decomposing the decline of cash assistance in the United States, 1993 to 2016. Demography, 58(3), 1119–1141. 10.1215/00703370-9157471 33881488

[cl21414-bib-0170] Pattaro, S. , Bailey, N. , Williams, E. , Gibson, M. , Wells, V. , Tranmer, M. , & Dibben, C. (2022). The impacts of benefit sanctions: A scoping review of the quantitative research evidence. Journal of Social Policy, 51(3), 611–653. 36000019 10.1017/S0047279421001069 PMC7613403

[cl21414-bib-0171] Peer, A. (2023). Global poverty: Facts, FAQs, and how to help [Global poverty]. World Vision.

[cl21414-bib-0172] Pinto, A. D. , Perri, M. , Pedersen Cheryl, L. , Aratangy, T. , Pinky, H. A. , Hwang, S. W. (2021). Exploring different methods to evaluate the impact of basic income interventions: A systematic review. International Journal for Equity in Health, 20(142), 1–20. 10.1186/s12939-021-01479-2 34134715 PMC8206888

[cl21414-bib-0173] Policy Options . (2004). New century, new risks: The Marsh Report and the post‐war welfare state in Canada. Policy Options. https://policyoptions.irpp.org/magazines/social-policy-in-the-21st-century/new-century-new-risks-the-marsh-report-and-the-post-war-welfare-state-in-canada/

[cl21414-bib-0174] Poverty Analysis Discussion Group . (2012). Understanding poverty and wellbeing: A note with implications for research and policy. Department for International Development (DFID).

[cl21414-bib-0175] Power, E. , Abercrombie, D. , Fafard St‐Germain, A.‐A. , & Vanderkooy, P. (2016). Prevalence, severity and impact of household food insecurity: A serious public health issue. Dietitians of Canada. https://www.dietitians.ca/DietitiansOfCanada/media/Documents/Resources/HFI-Background-DC-FINAL.pdf?ext=.pdf

[cl21414-bib-0176] Ramsey, R. , Giskes, K. , Turrell, G. , & Gallegos, D. (2011). Food insecurity among Australian children: Potential determinants, health and developmental consequences. Journal of Child Health Care, 15(4), 401–416. 22199175 10.1177/1367493511423854

[cl21414-bib-0177] RAND Corporation . (2018). 12‐Item short form survey from the RAND Medical Outcomes Study. Objective analysis. Effective Solutions.

[cl21414-bib-0178] Reed, H. , & Lansley, S . (2016). *Universal basic income: An idea whose time has come?* Compass.

[cl21414-bib-0179] Riches, G. , & Tarasuk, V. (2014). Canada: Thirty years of food charity and public policy neglect. In G. Riches , & T. Silvasti (Eds.), First world hunger revisited: Food charity or the right to food? (pp. 42–56). Palgrave Macmillan.

[cl21414-bib-0180] Rizvi, A. , Welch, V. , Gibson, M. , Labelle Patrick, R. , Pollard, C. , Wells George, A. , & Kristjansson, E. (2022). PROTOCOL: Effects of guaranteed basic income interventions on poverty‐related outcomes in high‐income countries: A systematic review. Campbell Systematic Reviews, 18(4), e1281. 10.1002/cl2.1281 36908842 PMC9538708

[cl21414-bib-0181] Robson, J. , & Schwartz, S. (2020). Who doesn't file a tax return? A portrait of non‐filers. Canadian Public Policy, 46(3), 323–339.

[cl21414-bib-0182] Romano, S. (2015). Idle paupers, scroungers and shirkers: Past and new social stereotypes of the undeserving welfare claimant in the UK. In L. Foster , A. Brunton , C. Deeming , & T. Haux (Eds.), In defence of welfare II (pp. 65–68). Policy Press.

[cl21414-bib-0183] Rose, J. (1985). From Bismark to Roosevelt: How the welfare began. Scholastic Update, 118, 13–16.

[cl21414-bib-0184] Salleh, M. R. (2008). Life event, stress and illness. The Malaysian Journal of Medical Sciences: MJMS, 15(4), 9–18. 22589633 PMC3341916

[cl21414-bib-0185] Sarabia, D. N. (2016). Consumption: The best way to measure poverty? BORGEN Magazine.

[cl21414-bib-0186] Sarlo, C. A. (2018). Poverty really isn't that hard to measure: Op‐ed. Fraser Institute.

[cl21414-bib-0187] Schenck‐Fontaine, A. , & Panico, L. (2019). Many kinds of poverty: Three dimensions of economic hardship, their combinations, and children's behavior problems. Demography, 56(6), 2279–2305. 31808103 10.1007/s13524-019-00833-y PMC7172985

[cl21414-bib-0188] Schünemann, H. J. , Higgins, J. P. T. , Vist, G. E. , Glasziou, P. , Akl, E. A. , Skoetz, N. , Guyatt, G. H. , & The Cochrane GRADEing Methods Group (formerly Applicability and Recommendations Methods Group) The Cochrane Statistical Methods Group . (2019). Completing ‘Summary of findings’ tables and grading the certainty of the evidence. In J. P. T. Higgins , J. Thomas , J. Chandler , M. Cumpston , T. Li , M. J. Page , & V. A. Welch (Eds.), Cochrane Handbook for Systematic Reviews of Interventions (pp. 375–402). John Wiley & Sons Ltd.

[cl21414-bib-0189] Schünemann, H. J. , Vist, G. E. , Higgins, J. P. T. , Santesso, N. , Deeks, J. J. , Glasziou, P. , Akl, E. , & Guyatt, G. H. (2023). *Chapter 15: Interpreting results and drawing conclusions [Chapter 15]. Cochrane handbook for systematic reviews of interventions. Version 6.4 edition*. Cochrane.

[cl21414-bib-0190] Sustainable Development Solutions Network . (2019). Multidimensional poverty index. SDSN – Indicators and a Monitoring Framework.

[cl21414-bib-0191] Stockton Economic Empowerment Demonstration . (2019). Our Vision for SEED: A discussion paper. https://www.stocktondemonstration.org/s/10-SEED-Discussion-Paper.pdf

[cl21414-bib-0192] Seligman, H. K. , & Schillinger, D. (2010). Hunger and socioeconomic disparities in chronic disease. The New England Journal of Medicine, 363(1), 6–9. 20592297 10.1056/NEJMp1000072

[cl21414-bib-0193] Sharma Waddington, H. , & Cairncross, S. (2021). PROTOCOL: Water, sanitation and hygiene for reducing childhood mortality in low‐ and middle‐income countries. Campbell Systematic Reviews, 17(1), e1135. 37050969 10.1002/cl2.1135 PMC8356349

[cl21414-bib-0194] Shea, B. J. , Reeves, B. C. , Wells, G. , Thuku, M. , Hamel, C. , Moran, J. , Moher, D. , Tugwell, P. , Welch, V. , Kristjansson, E. , & Henry, D. A. (2017). AMSTAR 2: A critical appraisal tool for systematic reviews that include randomised or non‐randomised studies of healthcare interventions, or both. BMJ, 358, 9. 10.1136/bmj.j4008 PMC5833365 28935701

[cl21414-bib-0195] Sheils McNamee, M. (2023). Universal basic income: Plans drawn up for £1,600 a month trial in England. BBC News. https://www.bbc.com/news/uk-65806599

[cl21414-bib-0196] Simpson, W. , Mason, G. , & Godwin, R. (2017). The Manitoba basic annual income experiment: Lessons learned 40 years later. Canadian Public Policy, 43(1), 85–104.

[cl21414-bib-0197] Somers, M. A. , Muffels, R. , & Künn‐Nelen, A. (2021). Micro‐ and macro economic effects of unconditional basic income and participation income: A systematic review.

[cl21414-bib-0198] SRI International . (1983). *Final report of the seattle‐denver income maintenance experiment* (Vol. 1). US Department of Health and Human Services.

[cl21414-bib-0199] Standing, G. (2019). Basic income as common dividends: Piloting a transformative policy. Progressive Economy Forum.

[cl21414-bib-0200] Standing, G. (2021). Basic income pilots: Uses, limitations and design principles. Basic Income Studies, 16(1), 75–99.

[cl21414-bib-0201] Statistics Canada . (2021). Dimensions of poverty hub. Statistics Canada.

[cl21414-bib-0202] Statistics Canada . (2022). Dictionary, Census of Population, 2021 – Market Basket Measure (MBM).

[cl21414-bib-0203] Statistics Canada . (2023). Wages by deciles, 1997 to 2022. https://www150.statcan.gc.ca/n1/pub/14-28-0001/2023001/article/00005-eng.htm

[cl21414-bib-0204] Sterne, J. A. C. , Hernán Miguel, A. , Reeves Barnaby, C. , Savović, J. , Berkman Nancy, D. , Viswanathan, M. , Henry, D. , Altman Douglas, G. , Ansari Mohammed, T. , Boutron, I. , Carpenter James, R. , Chan, A.‐W. , Churchill, R. , Deeks Jonathan, J. , Hróbjartsson, A. , Kirkham, J. , Jüni, P. , Loke Yoon, K. , Pigott Theresa, D. , … Higgins Julian, P. T. (2016). ROBINS‐I: A tool for assessing risk of bias in non‐randomised studies of interventions. BMJ, 355, i4919. 27733354 10.1136/bmj.i4919 PMC5062054

[cl21414-bib-0205] Stone, C. , Trisi, D. , Sherman, A. , & Beltrán, J. (2020). A guide to statistics on historical trends in income inequality. Center on Budget and Policy Priorities.

[cl21414-bib-0206] Tarasuk, V. , Fafard St‐Germain, A.‐A. , & Loopstra, R. (2020). The relationship between food banks and food insecurity: Insights from Canada. VOLUNTAS: International Journal of Voluntary and Nonprofit Organizations, 31(5), 841–852. 10.1007/s11266-019-00092-w

[cl21414-bib-0207] Tarasuk, V. , & Mitchell, A. (2020). Household food insecurity in Canada, 2017–18. Research to identify policy options to reduce food insecurity (PROOF). https://proof.utoronto.ca/wp-content/uploads/2020/03/Household-Food-Insecurity-in-Canada-2017-2018-Full-Reportpdf.pdf

[cl21414-bib-0208] Taylor Joseph, A. , Pigott, T. , & Williams, R. (2022). Promoting knowledge accumulation about intervention effects: Exploring strategies for standardizing statistical approaches and effect size reporting. Educational Researcher, 51(1), 72–80.

[cl21414-bib-0209] Thoits Peggy, A. (2010). Stress and health: Major findings and policy implications. Journal of Health and Social Behavior, 51(1_Suppl.), S41–S53. 20943582 10.1177/0022146510383499

[cl21414-bib-0210] Thomas Margaret, M. C. , Miller Daniel, P. , & Morrissey Taryn, W. (2019). Food insecurity and child health. Pediatrics, 144(4), e20190397. 31501236 10.1542/peds.2019-0397

[cl21414-bib-0211] Toppenberg, L. (2017). Exploring food insecurity policy through multidimensional poverty measures. Texas Medical Center Dissertations (via ProQuest) (pp. 1–71).

[cl21414-bib-0212] Trattner Walter, I. (2007). From poor law to welfare state: A history of social welfare in America. Simon and Schuster.

[cl21414-bib-0213] United Kingdom Government . (2014). Universal credit. GOV.UK.

[cl21414-bib-0214] UK Government . (2021). United Kingdom food security report 2021: Theme 4: Food security at household level. GOV.UK. https://www.gov.uk/government/statistics/united-kingdom-food-security-report-2021/united-kingdom-food-security-report-2021-theme-4-food-security-at-household-level

[cl21414-bib-0215] UK Government . (2023). Universal Credit. GOV.UK. https://www.gov.uk/universal-credit/how-your-earnings-affect-your-payments

[cl21414-bib-0216] United Nations Department of Economic and Social Affairs . (2022). Statistical annex – Country classifications. In G. Luchsinger (Ed.), World economic situation and prospects 2022 (pp. 163–171). United Nations. https://www.un.org/development/desa/dpad/wp-content/uploads/sites/45/WESP2022_ANNEX.pdf

[cl21414-bib-0217] United Nations . (2020). Inequality – Bridging the Divide. UN75: 2020 and beyond 2020. https://www.un.org/en/un75/inequality-bridging-divide

[cl21414-bib-0218] US Census Bureau . (2021). Consumer Expenditure Survey (CE). Census.gov. https://www.census.gov/programs-surveys/ce.html

[cl21414-bib-0219] US Department of Agriculture . (2022). USDA ERS – Survey Tools. USDA Economic Research Service. https://www.ers.usda.gov/topics/food-nutrition-assistance/food-security-in-the-u-s/survey-tools/#household

[cl21414-bib-0220] Van Mechelen, N. , & Janssens, J. (2017). Who is to blame? An overview of the factors contributing to the non‐take‐up of social rights. Herman Deleeck Centre for Social Policy, University of Antwerp. https://repository.uantwerpen.be/docman/irua/cf28ca/147576.pdf

[cl21414-bib-0221] Van Parijs, P. (2004). Basic income: A simple and powerful idea for the twenty‐first century. Politics & Society, 32(1), 7–39. 10.1177/0032329203261095

[cl21414-bib-0222] Van Parijs, P. , & Vanderborght, Y. (2017). Basic income: A radical proposal for a free society and a sane economy. Harvard University Press.

[cl21414-bib-0223] Versace, C. , Hawkins Lenore, E. , & Abssy, M. (2021). World reimagined: The rise of the global middle class. Nasdaq. https://www.nasdaq.com/articles/world-reimagined%3A-the-rise-of-the-global-middle-class-2021-07-09

[cl21414-bib-0224] Waddington, H. , White, H. , Snilstveit, B. , Hombrados Jorge, G. , Vojtkova, M. , Davies, P. , Bhavsar, A. , Eyers, J. , Koehlmoos Tracey, P. , Petticrew, M. , Valentine Jeffrey, C. , & Tugwell, P. (2012). How to do a good systematic review of effects in international development: A tool kit. Journal of Development Effectiveness, 4(3), 359–387. 10.1080/19439342.2012.711765

[cl21414-bib-0225] Waddington, H. , Aloe Ariel, M. , Becker Betsy, J. , Djimeu Eric, W. , Hombrados Jorge, G. , Tugwell, P. , Wells, G. , & Reeves, B. (2017). Quasi‐experimental study designs series—Paper 6: Risk of bias assessment. Journal of Clinical Epidemiology, 89, 43–52. 28351693 10.1016/j.jclinepi.2017.02.015

[cl21414-bib-0226] Welch, V. , Petticrew, M. , Tugwell, P. , Moher, D. , O'Neill, J. , Waters, E. , White, H. , & PRISMA‐Equity Bellagio group . (2012). PRISMA‐equity 2012 extension: Reporting guidelines for systematic reviews with a focus on health equity. PLoS Medicine, 9(10), e1001333. 23222917 10.1371/journal.pmed.1001333 PMC3484052

[cl21414-bib-0227] Welch, V. A. , Norheim, O. F. , Jull, J. , Cookson, R. , Sommerfelt, H. , & Tugwell, P. (2017). Symposium CONSORT‐equity and Boston equity. CONSORT‐Equity 2017 extension and elaboration for better reporting of health equity in randomised trials. BMJ, 359, j5085. 10.1136/bmj.j5085 29170161

[cl21414-bib-0228] West, S. , & Castro, A. (2023). Impact of guaranteed income on health, finances, and agency: Findings from the Stockton randomized controlled trial. Journal of Urban Health, 100(2), 227–244. 10.1007/s11524-023-00723-0 37037977 PMC10160253

[cl21414-bib-0229] Wheeler, B. (2015). How did we get to this welfare state? BBC News. https://www.bbc.com/news/uk-politics-33256084

[cl21414-bib-0230] Whelan, C. T. , Nolan, B. , & Maître, B. (2014). Multidimensional poverty measurement in Europe: An application of the adjusted headcount approach. Journal of European Social Policy, 24(2), 183–197.

[cl21414-bib-0231] Widerquist, K. , Noguera José, A. , & Vanderborght, Y. (2013). Introduction: The economics of basic income. In K. Widerquist , J. Noguera , Y. Vandeborght , & J. Wispelaere (Eds.), Basic income: An anthology of contemporary research (pp. 190–194). Blackwell Publishing.

[cl21414-bib-0232] Wilson, N. , & McDaid, S. (2021). The mental health effects of a universal basic income: A synthesis of the evidence from previous pilots. Social Science & Medicine, 287, 114374. 10.1016/j.socscimed.2021.114374 34534779

[cl21414-bib-0233] Winchester, N. (2021). Universal credit: An end to the uplift. UK Parliament – House of Lords Library.

[cl21414-bib-0234] Wolfson, M. (2018). How a guaranteed income could work. Policy Options.

[cl21414-bib-0235] World Bank Group . (2023). World Bank country and lending groups – World Bank Data help desk. World Bank Group. https://datahelpdesk.worldbank.org/knowledgebase/articles/906519-world-bank-country-and-lending-groups

[cl21414-bib-0236] Yang, J. , Mohan, G. , Pipil, S. , & Fukushi, K. (2021). Review on basic income (BI): Its theories and empirical cases. Journal of Social and Economic Development, 23, 203–239.

