# Supplementary material for: Effects of guaranteed basic income interventions on poverty‐related outcomes in high‐income countries: A systematic review and meta‐analysis
Source: Campbell Syst Rev. 2024 Jun 16;20(2):e1414. doi: 10.1002/cl2.1414 (PMC11180702; doi:10.1002/cl2.1414)
Supplement: Supplementary file 1 — Supporting information. [file CL2-20-e1414-s002.docx]

# Characteristics of studies

## Characteristics of included studies [ordered by study ID]

#### Bonilla 2019

***Study characteristics***

Notes

#### Calnitsky 2019

***Study characteristics***

Notes

#### Calnitsky 2021

***Study characteristics***

Notes

#### Elesh 1977

***Study characteristics***

Notes

#### Forget 2011/2013

***Study characteristics***

Notes

#### Gonalons-Pons 2021

***Study characteristics***

Notes

#### Groeneveld 1979

***Study characteristics***

Notes

#### Kaluzny 1979

***Study characteristics***

Notes

#### Kehrer 1979

***Study characteristics***

Notes

#### Kerachsky 1977

***Study characteristics***

Notes

#### Ladinsky 1977

***Study characteristics***

Notes

#### Lassander 2021

***Study characteristics***

Notes

#### Mallar 1977

***Study characteristics***

Notes

#### Manheim 1979

***Study characteristics***

Notes

#### Maynard 1977

***Study characteristics***

Notes

#### Maynard 1979

***Study characteristics***

Notes

#### McDonald 1979

***Study characteristics***

Notes

#### McIntyre 2016a

***Study characteristics***

Notes

#### Middleton 1977

***Study characteristics***

Notes

#### Muffels 2021

***Study characteristics***

Notes

#### Nicholson 1977

***Study characteristics***

Notes

#### O’Connor 1979

***Study characteristics***

Notes

#### Simanainen 2021

***Study characteristics***

Notes

#### Thoits 1979

***Study characteristics***

Notes

#### Todeschini 2019

***Study characteristics***

Notes

#### Venti 1984

***Study characteristics***

Notes

#### West 2021

***Study characteristics***

Notes

## Characteristics of excluded studies [ordered by study ID]

| Study | Reason for exclusion |
| --- | --- |
| Calnitsky 2017 | Ambiguous poverty-related outcome (employment) |
| Choudhry 1995 | Ambiguous poverty-related outcome (marital dissolution) |
| Choudhry 2001 | Ambiguous poverty-related outcome (household transition dynamics) |
| Cogan 1983 | Ambiguous poverty-related outcome (employment) |
| Greenberg 1983 | Ambiguous poverty-related outcome (employment) |
| Groeneveld 1980 | Ambiguous poverty-related outcome (marital dissolution) |
| Hannan 1977 | Ambiguous poverty-related outcome (marital dissolution) |
| Heffernan 1977 | Ambiguous poverty-related outcome (awareness of social services) |
| Hum 1992 | Ambiguous poverty-related outcome (marital dissolution) |
| Keeley 1980a | Ambiguous poverty-related outcome (migration) |
| Keeley 1980b | Ambiguous poverty-related outcome (fertility) |
| Keeley 1987 | Ambiguous poverty-related outcome (marital dissolution) |
| McDowell 2020 | Ineligible study design (cross-sectional with one timepoint) |
| McDowell 2021 | Ineligible study design (cross-sectional with one timepoint) |
| McIntyre 2016b | Ineligible study design (cross-sectional with one timepoint) |
| Pencavel 1982 | Ambiguous poverty-related outcome (employment) |
| Robins 1980 | Ambiguous poverty-related outcome (employment) |
| West 1980 | Ambiguous poverty-related outcome (employment) |

# Appendices

## Appendix 1. Complete search strategy

The following databases were searched on May 16 or May 17, 2022:

- APA PsycInfo (Ovid) – 387 results
- Academic Search Complete (EBSCOhost) – 2125 results
- Business Source Complete (EBSCOhost) – 2785 results
- CENTRAL (Ovid) – 73 results
- CINAHL (EBSCOhost) – 542 results
- EconLit (EBSCOhost) – 3080 results
- Embase (Ovid) – 761 results
- Global Health (EBSCOhost) – 487 results
- International Bibliography of the Social Sciences (ProQuest) – 2911 results
- International Political Science Abstracts (EBSCOhost) – 236 results
- MEDLINE (Ovid) – 610 results
- PAIS Index (ProQuest) – 1198 results
- ProQuest Dissertations & Theses Global (PQDT) – 1014 results
- Sociological Abstracts (including Social Services Abstracts; ProQuest) – 2341 results
- Web of Science (various indexes; Clarivate) – 4745 results
- Worldwide Political Science Abstracts (ProQuest) – 1181 results

The following strategies were used for each database.

**APA PsycInfo (Ovid)**

1. (“basic income”).ti,ab.
2. ((guarantee* or universal*) adj2 (income or allowance*)).ti,ab.
3. (minim* adj2 income).ti,ab.
4. (citizen* adj2 (income or dividend*)).ti,ab.
5. (“negative income tax*“).ti,ab.
6. (optimal adj2 income).ti,ab.
7. (income adj2 maintenance).ti,ab.
8. (unconditional adj2 (cash or transfer*)).ti,ab.
9. mincome*.ti,ab.
10. demogrant*.ti,ab.
11. or/1-10

**Academic Search Complete (EBSCOhost)**

1. DE “income maintenance programs” OR DE “basic income” OR DE “negative income tax”
2. TI(“basic income”) OR AB(“basic income”)
3. TI((guarantee* or universal*) N2 (income or allowance*)) OR AB((guarantee* or universal*) N2 (income or allowance*))
4. TI(minim* N2 income) OR AB(minim* N2 income)
5. TI(citizen* N2 (income or dividend*)) OR AB(citizen* N2 (income or dividend*))
6. TI(“negative income tax*“) OR AB(“negative income tax*“)
7. TI(optimal N2 income) OR AB(optimal N2 income)
8. TI(income N2 maintenance) OR AB(income N2 maintenance)
9. TI(unconditional N2 (cash or transfer*)) OR AB(unconditional N2 (cash or transfer*))
10. TI(mincome*) OR AB(mincome*)
11. TI(demogrant*) OR AB(demogrant*)
12. S1 OR S2 OR S3 OR S4 OR S5 OR S6 OR S7 OR S8 OR S9 OR S10 OR S11
13. From results, limit to “Academic Journals” as “Source Type”

**Business Source Complete (EBSCOhost)**

1. DE “income maintenance programs” OR DE “basic income” OR DE “negative income tax”
2. TI(“basic income”) OR AB(“basic income”)
3. TI((guarantee* or universal*) N2 (income or allowance*)) OR AB((guarantee* or universal*) N2 (income or allowance*))
4. TI(minim* N2 income) OR AB(minim* N2 income)
5. TI(citizen* N2 (income or dividend*)) OR AB(citizen* N2 (income or dividend*))
6. TI(“negative income tax*“) OR AB(“negative income tax*“)
7. TI(optimal N2 income) OR AB(optimal N2 income)
8. TI(income N2 maintenance) OR AB(income N2 maintenance)
9. TI(unconditional N2 (cash or transfer*)) OR AB(unconditional N2 (cash or transfer*))
10. TI(mincome*) OR AB(mincome*)
11. TI(demogrant*) OR AB(demogrant*)
12. S1 OR S2 OR S3 OR S4 OR S5 OR S6 OR S7 OR S8 OR S9 OR S10 OR S11
13. From results, limit to “Academic Journals” as “Source Type”

**CENTRAL (Ovid)**

1. (“basic income”).ti,ab.
2. ((guarantee* or universal*) adj2 (income or allowance*)).ti,ab.
3. (minim* adj2 income).ti,ab.
4. (citizen* adj2 (income or dividend*)).ti,ab.
5. (“negative income tax*“).ti,ab.
6. (optimal adj2 income).ti,ab.
7. (income adj2 maintenance).ti,ab.
8. (unconditional adj2 (cash or transfer*)).ti,ab.
9. mincome*.ti,ab.
10. demogrant*.ti,ab.
11. or/1-10

**CINAHL (EBSCOhost)**

1. TI(“basic income”) OR AB(“basic income”)
2. TI((guarantee* or universal*) N2 (income or allowance*)) OR AB((guarantee* or universal*) N2 (income or allowance*))
3. TI(minim* N2 income) OR AB(minim* N2 income)
4. TI(citizen* N2 (income or dividend*)) OR AB(citizen* N2 (income or dividend*))
5. TI(“negative income tax*“) OR AB(“negative income tax*“)
6. TI(optimal N2 income) OR AB(optimal N2 income)
7. TI(income N2 maintenance) OR AB(income N2 maintenance)
8. TI(unconditional N2 (cash or transfer*)) OR AB(unconditional N2 (cash or transfer*))
9. TI(mincome*) OR AB(mincome*)
10. TI(demogrant*) OR AB(demogrant*)
11. S1 OR S2 OR S3 OR S4 OR S5 OR S6 OR S7 OR S8 OR S9 OR S10

**EconLit (EBSCOhost)**

1. TI(“basic income”) OR AB(“basic income”)
2. TI((guarantee* or universal*) N2 (income or allowance*)) OR AB((guarantee* or universal*) N2 (income or allowance*))
3. TI(minim* N2 income) OR AB(minim* N2 income)
4. TI(citizen* N2 (income or dividend*)) OR AB(citizen* N2 (income or dividend*))
5. TI(“negative income tax*“) OR AB(“negative income tax*“)
6. TI(optimal N2 income) OR AB(optimal N2 income)
7. TI(income N2 maintenance) OR AB(income N2 maintenance)
8. TI(unconditional N2 (cash or transfer*)) OR AB(unconditional N2 (cash or transfer*))
9. TI(mincome*) OR AB(mincome*)
10. TI(demogrant*) OR AB(demogrant*)
11. S1 OR S2 OR S3 OR S4 OR S5 OR S6 OR S7 OR S8 OR S9 OR S10

**Embase (Ovid)**

1. (“basic income”).ti,ab.
2. ((guarantee* or universal*) adj2 (income or allowance*)).ti,ab.
3. (minim* adj2 income).ti,ab.
4. (citizen* adj2 (income or dividend*)).ti,ab.
5. (“negative income tax*“).ti,ab.
6. (optimal adj2 income).ti,ab.
7. (income adj2 maintenance).ti,ab.
8. (unconditional adj2 (cash or transfer*)).ti,ab.
9. mincome*.ti,ab.
10. demogrant*.ti,ab.
11. or/1-10

**Global Health (EBSCOhost)**

1. TI(“basic income”) OR AB(“basic income”)
2. TI((guarantee* or universal*) N2 (income or allowance*)) OR AB((guarantee* or universal*) N2 (income or allowance*))
3. TI(minim* N2 income) OR AB(minim* N2 income)
4. TI(citizen* N2 (income or dividend*)) OR AB(citizen* N2 (income or dividend*))
5. TI(“negative income tax*“) OR AB(“negative income tax*“)
6. TI(optimal N2 income) OR AB(optimal N2 income)
7. TI(income N2 maintenance) OR AB(income N2 maintenance)
8. TI(unconditional N2 (cash or transfer*)) OR AB(unconditional N2 (cash or transfer*))
9. TI(mincome*) OR AB(mincome*)
10. TI(demogrant*) OR AB(demogrant*)
11. S1 OR S2 OR S3 OR S4 OR S5 OR S6 OR S7 OR S8 OR S9 OR S10

**International Bibliography of the Social Sciences (ProQuest)**

1. TI,AB(“basic income”)
2. TI,AB((guarantee* or universal*) N/2 (income or allowance*))
3. TI,AB(minim* N/2 income)
4. TI,AB(citizen* N/2 (income or dividend*))
5. TI,AB(“negative income tax*“)
6. TI,AB(optimal N/2 income)
7. TI,AB(income N/2 maintenance)
8. TI,AB(unconditional N/2 (cash or transfer*))
9. TI,AB(mincome*)
10. TI,AB(demogrant*)
11. 1 OR 2 OR 3 OR 4 OR 5 OR 6 OR 7 OR 8 OR 9 OR 10
12. From results, limited to “Scholarly Journals OR Dissertations & Theses OR Other Sources OR Reports” as Source Types

**International Political Science Abstracts (EBSCOhost)**

1. TI(“basic income”) OR AB(“basic income”)
2. TI((guarantee* or universal*) N2 (income or allowance*)) OR AB((guarantee* or universal*) N2 (income or allowance*))
3. TI(minim* N2 income) OR AB(minim* N2 income)
4. TI(citizen* N2 (income or dividend*)) OR AB(citizen* N2 (income or dividend*))
5. TI(“negative income tax*“) OR AB(“negative income tax*“)
6. TI(optimal N2 income) OR AB(optimal N2 income)
7. TI(income N2 maintenance) OR AB(income N2 maintenance)
8. TI(unconditional N2 (cash or transfer*)) OR AB(unconditional N2 (cash or transfer*))
9. TI(mincome*) OR AB(mincome*)
10. TI(demogrant*) OR AB(demogrant*)
11. S1 OR S2 OR S3 OR S4 OR S5 OR S6 OR S7 OR S8 OR S9 OR S10

**MEDLINE (Ovid)**

1. (“basic income”).ti,ab,kf.
2. ((guarantee* or universal*) adj2 (income or allowance*)).ti,ab,kf.
3. (minim* adj2 income).ti,ab,kf.
4. (citizen* adj2 (income or dividend*)).ti,ab,kf.
5. (“negative income tax*“).ti,ab,kf.
6. (optimal adj2 income).ti,ab,kf.
7. (income adj2 maintenance).ti,ab,kf.
8. (unconditional adj2 (cash or transfer*)).ti,ab,kf.
9. mincome*.ti,ab,kf.
10. demogrant*.ti,ab,kf.
11. or/1-10

**PAIS Index (ProQuest)**

1. MAINSUBJECT.EXACT(“Income Maintenance Programs”)
2. TI,AB(“basic income”)
3. TI,AB((guarantee* or universal*) N/2 (income or allowance*))
4. TI,AB(minim* N/2 income)
5. TI,AB(citizen* N/2 (income or dividend*))
6. TI,AB(“negative income tax*“)
7. TI,AB(optimal N/2 income)
8. TI,AB(income N/2 maintenance)
9. TI,AB(unconditional N/2 (cash or transfer*))
10. TI,AB(mincome*)
11. TI,AB(demogrant*)
12. 1 OR 2 OR 3 OR 4 OR 5 OR 6 OR 7 OR 8 OR 9 OR 10 OR 11
13. From results, limited to “Scholarly Journals OR Reports OR Dissertations & Theses” as Source Types

**Sociological Abstracts (including Social Services Abstracts; ProQuest)**

1. MAINSUBJECT.EXACT(“Income Maintenance Programs”)
2. TI,AB(“basic income”)
3. TI,AB((guarantee* or universal*) N/2 (income or allowance*))
4. TI,AB(minim* N/2 income)
5. TI,AB(citizen* N/2 (income or dividend*))
6. TI,AB(“negative income tax*“)
7. TI,AB(optimal N/2 income)
8. TI,AB(income N/2 maintenance)
9. TI,AB(unconditional N/2 (cash or transfer*))
10. TI,AB(mincome*)
11. TI,AB(demogrant*)
12. 1 OR 2 OR 3 OR 4 OR 5 OR 6 OR 7 OR 8 OR 9 OR 10 OR 11
13. From results, limited to “Scholarly Journals OR Dissertations & Theses OR Conference Papers & Proceedings OR Working Papers OR Other Sources” as Source Types

**Web of Science (Science Citation Index, Social Sciences Citation Index, Arts & Humanities Citation Index, Emerging Sources Citation Index, Conference Proceedings Citation Index – Science, Conference Proceedings Citation Index – Social Sciences & Humanities; Clarivate)**

1. TS=(“basic income”)
2. TS=((guarantee* or universal*) NEAR/2 (income or allowance*))
3. TS=(minim* NEAR/2 income)
4. TS=(citizen* NEAR/2 (income or dividend*))
5. TS=(“negative income tax*“)
6. TS=(optimal NEAR/2 income)
7. TS=(income NEAR/2 maintenance)
8. TS=(unconditional NEAR/2 (cash or transfer*))
9. TS=(mincome*)
10. TS=(demogrant*)
11. #1 OR #2 OR #3 OR #4 OR #5 OR #6 OR #7 OR #8 OR #9 OR #10

**Search strategies used to identify other review articles**

*Cochrane Database of Systematic Reviews (Ovid)*

1. (“basic income”).ti,ab.
2. ((guarantee* or universal*) adj2 (income or allowance*)).ti,ab.
3. (minim* adj2 income).ti,ab.
4. (citizen* adj2 (income or dividend*)).ti,ab.
5. (“negative income tax*“).ti,ab.
6. (optimal adj2 income).ti,ab.
7. (income adj2 maintenance).ti,ab.
8. (unconditional adj2 (cash or transfer*)).ti,ab.
9. mincome*.ti,ab.
10. demogrant*.ti,ab.
11. or/1-10

*Campbell Systematic Reviews (Wiley)*

- “basic income” OR “unconditional cash transfer” OR “unconditional cash transfers” OR “negative income tax” OR “guaranteed annual income”
- Two searches were done: one limiting results to keywords appearing in the title and the other limiting to the abstract.

*Epistemonikos (Wiley)*

- “basic income” OR ((guarantee* OR universal*) NEAR/2 (income OR allowance*)) OR (minim* NEAR/2 income) OR (citizen* NEAR/2 (income or dividend*)) OR “negative income tax*“ OR (optimal NEAR/2 income) OR (income NEAR/2 maintenance) OR (unconditional NEAR/2 (cash or transfer*)) OR mincome* OR demogrant*

*Social Systems Evidence (https://www.socialsystemsevidence.org/)*

- “basic income” OR “unconditional cash transfer” OR “unconditional cash transfers” OR “negative income tax” OR “guaranteed annual income”

*Google Scholar (https://scholar.google.com)*

- intitle:“basic income” OR intitle:“unconditional cash transfer” OR intitle:“unconditional cash transfers” OR intitle:“negative income tax” OR intitle:“guaranteed annual income” intitle:review OR intitle:meta
- Search results were limited to 2017 to 2022.

## Appendix 2. Study inclusion and exclusion criteria

|  | **Include** | **Exclude** |
| --- | --- | --- |
| **Article type** | Primary research | - any literature that synthesizes, summarizes or refers to the results of primary research - e.g. reviews, compilations, news and magazine articles, editorials, opinion pieces, textbook chapters, blogs  - proposals for studies, programs or policies |
| **Intervention** | Any cash transfer intervention for adults (18+ years old) that: (1) is unconditional, (2) has regular payment intervals, (3) provides a guaranteed minimum amount, and (4) provides fixed or predictable amounts  Negative income tax (NIT) interventions are eligible if receipt of benefits is unconditional | - interventions that are conditional - e.g. require having or seeking employment, taking training courses, participating in educational or counselling programs, having children of a certain age, caring for adults, having a disability  - unconditional cash transfers of amounts that vary by more than 10% during the study period due to changes in program funding/budget  - interventions that provide in-kind benefits (e.g. food vouchers, store gift cards, paid courses/training) |
| **Study design** | Any design that collects quantitative or quantifiable data about the effects of GBI on recipients  Multi-arm studies if one or more intervention arms meet the inclusion criteria | - simulations and predictive modelling (i.e. with no analysis of empirical data about GBI recipients)  - cross-sectional studies using data from a single time point  - interrupted time series (ITS) with less than three time points before and three time points after the intervention  - qualitative studies - e.g. case reports, interviews, focus groups  - process evaluations |
| **Setting** | Any setting in high-income countries classified as developed by UN DESA* | Not in a developed high-income country* |
| **Participants** | Persons living in a developed high-income country (including children of GBI recipients) | People and organizations involved with the study but not receiving GBI benefits (e.g. program administrators and staff, businesses offering employment) |
|  |  |  |

*Developed high-income countries are listed in Table A of the Statistical Annex of *World Economic Situation and Prospects 2022* (https://www.un.org/development/desa/dpad/wp-content/uploads/sites/45/WESP2022_ANNEX.pdf)

## Appendix 3. Data extraction template

| **General information** | |
| --- | --- |
| Covidence ID# |  |
| Reviewer’s initials |  |
| First author’s last name and year |  |
| Corresponding author’s contact info |  |
| Source (e.g. journal name, organization/government name) |  |
| Publication type | Select from: journal article, report, other (please specify) |
| Country | Select from: Canada, Finland, France, Germany, Italy, Japan, Netherlands, United Kingdom, Spain, United States, other (please specify) |
| Region (city, state/province, or region described in study) |  |
| Study start date (MM/YYYY) |  |
| Study end date (MM/YYYY) |  |
| Duration of follow-up (baseline to final measurement) |  |
| Study objectives (if presented) |  |
| Main conclusions (as reported) |  |
| Funder |  |
| Ethics approval |  |
| **Methods** | |
| Study design | Select from: RCT, cRCT, before-after (w/o control), CBA, RDD, ITS, other (please specify) |
| Statistical methods used | (Can use article text with page numbers) |
| Number of intervention arms |  |
| Description of intervention(s) | (Can use article text with page numbers) |
| Type of control/comparison | Select from: no intervention, other intervention, no control/comparison group, other control (please specify) |
| Allocation type | Select from: individual, household |
| Units of analysis | Individual, household and/or community level |
| Sociodemographic characteristics used to describe sample and/or compare groups at baseline |  |
| Sociodemographic characteristics used in subgroup analyses of intervention effects |  |
| **Population** | |
| Setting (e.g. urban, low-income neighbourhood, rural) |  |
| Age (mean, SD, range, as reported) |  |
| Sociodemographic characteristics for eligibility in study | e.g. income below x, age between x and y, sex, released from prison |
| Reasons for exclusion from study (if reported) |  |
| Total number of participants at baseline |  |
| Number of participants in each group at baseline |  |
| Number of participants in each group at end of study |  |
| Number of participants lost in each group |  |
| **Outcomes** |  |
| Outcomes reported | e.g. food insecurity, subjective financial stress, education level, self-reported health, material deprivation index score |
| Method of assessment for each outcome | Type of scale, instrument, source of income data or health data, etc. |
| Validity of each measure (if reported) |  |
| Timing of outcome assessment (e.g. baseline, 18 months, 36 months) |  |
| **Results** |  |
| Effect estimates reported for each outcome  (Summarize separately for each intervention if multi-arm study)  (Note if reported effect estimates are adjusted for covariates) | e.g. mean difference and SD, *p*-value or confidence interval |
| Type of effect estimate for each outcome | e.g. standardized mean difference, difference in differences, mean ratio, odds ratio (for dichotomous measures) |
| Statistically significant differences across subgroups for each outcome (and each intervention if multi-arm study) |  |

## Appendix 4. Studies excluded after the screening stage or during data extraction

| **Study** | **Reason for exclusion** |
| --- | --- |
| Calnitsky 2017 | Ambiguous poverty-related outcome (employment) |
| Choudhry 1995 | Ambiguous poverty-related outcome (marital dissolution) |
| Choudhry 2001 | Ambiguous poverty-related outcome (household transition dynamics) |
| Cogan 1983 | Ambiguous poverty-related outcome (employment) |
| Greenberg 1983 | Ambiguous poverty-related outcome (employment) |
| Groeneveld 1980 | Ambiguous poverty-related outcome (marital dissolution) |
| Hannan 1977 | Ambiguous poverty-related outcome (marital dissolution) |
| Heffernan 1977 | Ambiguous poverty-related outcome (awareness of social services) |
| Hum 1992 | Ambiguous poverty-related outcome (marital dissolution) |
| Keeley 1980a | Ambiguous poverty-related outcome (migration) |
| Keeley 1980b | Ambiguous poverty-related outcome (fertility) |
| Keeley 1987 | Ambiguous poverty-related outcome (marital dissolution) |
| McDowell 2020 | Ineligible study design (cross-sectional with one timepoint) |
| McDowell 2021 | Ineligible study design (cross-sectional with one timepoint) |
| McIntyre 2016b | Ineligible study design (cross-sectional with one timepoint) |
| Pencavel 1982 | Ambiguous poverty-related outcome (employment) |
| Robins 1980 | Ambiguous poverty-related outcome (employment) |
| West 1980 | Ambiguous poverty-related outcome (employment) |

## Appendix 5. Included outcomes by study/experiment

### 5.1 Economic and material outcomes

| **Study/experiment** | **Outcome sub-category** | **Outcome** | **Instrument/scale/source of data** | **Timing of assessment** |
| --- | --- | --- | --- | --- |
| McIntyre 2016a, Canada public pension | Food insecurity | Food insecurity | Household Food Security Survey Module (HFSSM) | 2007-2008, 2009-2010, 2011-2012, and 2013 |
| Todeschini 2019, B-Mincome (Barcelona) | Food insecurity | Food insecurity | Study survey data | Baseline, 11-12 months, 21 months (July 2019) |
| Todeschini 2019, B-Mincome (Barcelona) | Food insecurity | Going to bed hungry | Study survey data | Baseline, 11-12 months, 21 months (July 2019) |
| Kaluzny 1979, Gary, USA | Non-food expenditures | Probability of becoming a homeowner | Study survey data | Baseline, 1 year, 2 year, 3 year |
| Kaluzny 1979, Gary, USA | Non-food expenditures | Rental expenditure | Study survey data | Baseline, 1 year, 2 year, 3 year |
| Ladinsky 1977, New Jersey, USA | Non-food expenditures | Lifestyle enhancement – value of appliances owned | Study survey data | Baseline (1st quarter), 6th quarter, 10th quarter |
| Ladinsky 1977, New Jersey, USA | Non-food expenditures | Lifestyle enhancement – value of cars owned | Study survey data | Baseline (1st quarter), 6th quarter, 10th quarter |
| Ladinsky 1977, New Jersey, USA | Non-food expenditures | Lifestyle enhancement – home improvements and repair | Study survey data | Baseline, 3rd quarter, 7th quarter, 11th quarter |
| Nicholson 1977, New Jersey, USA | Food expenditure | Expenditures - Food eaten at home | Study survey data | Unclear |
| Nicholson 1977, New Jersey, USA | Food expenditure | Expenditures - Food eaten out | Study survey data | Unclear |
| Nicholson 1977, New Jersey, USA | Non-food expenditures | Expenditures - Rent | Study survey data | 8th Quarter |
| Nicholson 1977, New Jersey, USA | Non-food expenditures | Expenditures - Total durables | Study survey data | Quarters 1, 2, 3, 4, 5, and 6 |
| Nicholson 1977, New Jersey, USA | Non-food expenditures | Expenditures - Clothing | Study survey data | Unclear |
| Nicholson 1977, New Jersey, USA | Non-food expenditures | Expenditures - Autos | Study survey data | Unclear |
| Nicholson 1977, New Jersey, USA | Non-food expenditures | Expenditures - Home production appliances | Study survey data | Unclear |
| Nicholson 1977, New Jersey, USA | Non-food expenditures | Expenditures - Furniture | Study survey data | Unclear |
| Nicholson 1977, New Jersey, USA | Non-food expenditures | Expenditures - Other appliances | Study survey data | Unclear |
| Nicholson 1977, New Jersey, USA | Personal finances | Home debt | Study survey data | 1st and 6th quarters |
| Nicholson 1977, New Jersey, USA | Personal finances | Total non-home debt | Study survey data | 1st and 6th quarters |
| Nicholson 1977, New Jersey, USA | Personal finances | Auto debt | Study survey data | 1st and 6th quarters |
| Nicholson 1977, New Jersey, USA | Personal finances | Medical debt | Study survey data | 1st and 6th quarters |
| Nicholson 1977, New Jersey, USA | Personal finances | All other debt (non-auto, non-medical) | Study survey data | 1st and 6th quarters |
| Nicholson 1977, New Jersey, USA | Personal finances | Financial assets | Study survey data | Unclear |
| Nicholson 1977, New Jersey, USA | Personal finances | Cash | Study survey data | Unclear |
| Todeschini 2019, B-Mincome (Barcelona) | Financial hardship | Falling behind in mortgage repayments or rent | Study survey data | Baseline, 11-12 months, 21 months (July 2019) |
| Todeschini 2019, B-Mincome (Barcelona) | Financial hardship | Borrowing money from family or friends | Study survey data | Baseline, 11-12 months, 21 months (July 2019) |
| Todeschini 2019, B-Mincome (Barcelona) | Financial hardship | Falling behind in utilities expenditures | Study survey data | Baseline, 11-12 months, 21 months (July 2019) |
| Todeschini 2019, B-Mincome (Barcelona) | Financial hardship | Forced to leave current residence | Study survey data | Baseline, 11-12 months, 21 months (July 2019) |
| Todeschini 2019, B-Mincome (Barcelona) | Material deprivation | Severe material deprivation | Study survey data | Baseline, 11-12 months, 21 months (July 2019) |
| Todeschini 2019, B-Mincome (Barcelona) | Material deprivation | Having roof leaks and moisture problems | Study survey data | Baseline, 11-12 months, 21 months (July 2019) |
| Todeschini 2019, B-Mincome (Barcelona) | Material deprivation | Material deprivation | Study survey data | Baseline, 11-12 months, 21 months (July 2019) |
| Todeschini 2019, B-Mincome (Barcelona) | Personal finances | Having outstanding debt | Study survey data | Baseline, 11-12 months, 21 months (July 2019) |
| Todeschini 2019, B-Mincome (Barcelona) | Personal finances | Buffer for unexpected financial expenses | Study survey data | Baseline, 11-12 months, 21 months (July 2019) |
| West 2021, SEED, USA | Aggregate expenditure | Aggregate spending data | Benefit (debit card) transaction data | Baseline, monthly |
| West 2021, SEED, USA | Personal finances | Ability to cover a $400 emergency | Study survey data | Baseline (-3 months), Month 1, Month 6, Month 12 |
| West 2021, SEED, USA | Personal finances | Income volatility - monthly fluctuation | Unclear | Baseline, monthly |

### 5.2 Physical health outcomes

| **Study/experiment** | **Outcome sub-category** | **Outcome** | **Instrument/scale/source of data** | **Timing of assessment** |
| --- | --- | --- | --- | --- |
| Elesh 1977, New Jersey, USA | Child health (self-reported) | Number of hospital days (children) | Study survey data | 2nd, 8th, and 12th quarters |
| Elesh 1977, New Jersey, USA | Child health (self-reported) | Number of bed days (children) | Study survey data | 2nd, 8th, and 12th quarters |
| Elesh 1977, New Jersey, USA | Child health (self-reported) | Number of chronic illnesses (children) | Study survey data | 2nd, 8th, and 12th quarters |
| Elesh 1977, New Jersey, USA | Physical health (self-reported) | Number of hospital days (husband and wife) | Quarterly interviews | Pre-enrollment, 2nd, 6th, 10th quarters |
| Elesh 1977, New Jersey, USA | Physical health (self-reported) | Number of chronic illnesses (husband and wife) | Quarterly interviews | Pre-enrollment, 2nd, 6th, 10th quarters |
| Forget 2011, Mincome (Canada) | Child health (administrative data) | Low birth weight | Manitoba Population Health Research Data Repository | Unclear |
| Forget 2011, Mincome (Canada) | Child health (administrative data) | At-risk birth weight | Manitoba Population Health Research Data Repository | Unclear |
| Forget 2011, Mincome (Canada) | Child health (administrative data) | Small-for-gestational age, newborns | Manitoba Population Health Research Data Repository | Unclear |
| Forget 2011, Mincome (Canada) | Physical health (records data) | Total hospital separations (1978 vs 1973) | Manitoba Health Services Insurance Plan registry | Baseline (1973) and 5 years later |
| Forget 2011, Mincome (Canada) | Physical health (records data) | Total hospital separations, all causes | Manitoba Health Services Insurance Plan registry | ITS model, 6-month intervals from 1970 to 1985 |
| Forget 2011, Mincome (Canada) | Physical health (records data) | Hospital separations, accidents and injuries | Manitoba Health Services Insurance Plan registry | ITS model, 6-month intervals from 1970 to 1985 |
| Forget 2013, Mincome (Canada) | Physical health (records data) | Overall hospital separations | Manitoba Health Services Insurance Plan registry | 6-month periods beginning 1974 |
| Forget 2013, Mincome (Canada) | Physical health (records data) | Hospital separations (accidents and injuries) | Manitoba Health Services Insurance Plan registry | 6-month periods beginning 1974 |
| Kehrer 1979, Gary, USA | Child health (administrative data) | Birth weight | Indiana State Board of Health records | Once (birth weight) |
| Kerachsky 1977, New Jersey, USA | Health-related impairments/limitations | Illness Interfering With Work - Adults | Study survey data | Pre-enrollment, 2nd, 6th, 10th quarters |
| Kerachsky 1977, New Jersey, USA | Health-related impairments/limitations | Illness Preventing Work - Adult Males Only | Study survey data | Pre-enrollment, 2nd, 6th, 10th quarters |
| Kerachsky 1977, New Jersey, USA | Physical health (self-reported) | Number of Times Entered Hospital - Adults | Study survey data | Pre-enrollment, 2nd, 6th, 10th quarters |
| Kerachsky 1977, New Jersey, USA | Physical health (self-reported) | Illness Lasting More Than 3 months - Adults | Study survey data | Pre-enrollment, 2nd, 6th, 10th quarters |
| Muffels 2021, Dutch (Netherlands) | Overall physical health (self-reported) | Subjective health | Single item question (5 choice Likert scale) | 16-24 months |
| O’Connor 1979, RIME, USA | Nutrition | Quality of dietary intake | 24-hour recall method | Quarter 3, quarter 11 (i.e. 2 years apart) |
| Simanainen 2021, Finnish BI Experiment | Health-related impairments/limitations | Having a disease, disability or mental disorder that hinders daily life | Study survey data | Unclear |
| Simanainen 2021, Finnish BI Experiment | Overall physical health (self-reported) | Subjective state of health | Study survey data | Unclear |
| Todeschini 2019, B-Mincome (Barcelona) | Child health (self-reported) | New obesity diagnostics on people under 15 years | Health care records | Baseline, 11-12 months, 21 months (July 2019) |
| Todeschini 2019, B-Mincome (Barcelona) | Child health (self-reported) | Number of young people in household reporting bad health | Study survey data | Baseline, 11-12 months, 21 months (July 2019) |
| Todeschini 2019, B-Mincome (Barcelona) | Overall physical health (self-reported) | Self-rated health being good, very good or excellent | Study survey data | Baseline, 11-12 months, 21 months (July 2019) |
| Todeschini 2019, B-Mincome (Barcelona) | Physical health (self-reported) | Self-reported serious health problems | Study survey data | Baseline, 11-12 months, 21 months (July 2019) |
| Todeschini 2019, B-Mincome (Barcelona) | Sleep | Quality of sleep | Study survey data | Baseline, 11-12 months, 21 months (July 2019) |
| Todeschini 2019, B-Mincome (Barcelona) | Sleep | Sleep deprivation - hours slept during last week | Study survey data | Baseline, 11-12 months, 21 months (July 2019) |
| West 2021, SEED, USA | Health-related impairments/limitations | Physical functioning | Short Form Health Survey 36 (SF-36) | Baseline (-3 months), Month 1, Month 6, Month 12 |
| West 2021, SEED, USA | Health-related impairments/limitations | Role limitations due to physical health | Short Form Health Survey 36 (SF-36) | Baseline (-3 months), Month 1, Month 6, Month 12 |
| West 2021, SEED, USA | Health-related impairments/limitations | Social functioning (due to health) | Short Form Health Survey 36 (SF-36) | Baseline (-3 months), Month 1, Month 6, Month 12 |
| West 2021, SEED, USA | Overall health and wellbeing | Overall Health and Wellbeing | Short Form Health Survey 36 (SF-36) | Baseline (-3 months), Month 1, Month 6, Month 12 |
| West 2021, SEED, USA | Overall physical health (self-reported) | General Health | Short Form Health Survey 36 (SF-36) | Baseline (-3 months), Month 1, Month 6, Month 12 |
| West 2021, SEED, USA | Physical health (self-reported) | Energy over fatigue | Short Form Health Survey 36 (SF-36) | Baseline (-3 months), Month 1, Month 6, Month 12 |
| West 2021, SEED, USA | Physical health (self-reported) | Pain (higher score means less) | Short Form Health Survey 36 (SF-36) | Baseline (-3 months), Month 1, Month 6, Month 12 |

### 5.3 Psychological and mental health outcomes

| **Study/experiment** | **Outcome sub-category** | **Outcome** | **Instrument/scale/source of data** | **Timing of assessment** |
| --- | --- | --- | --- | --- |
| Bonilla 2019, B-Mincome (Barcelona) | Life satisfaction | Life satisfaction | Self-rated, 0-10 scale | Baseline (Oct 2017), 1 year, and ~21/22 months |
| Forget 2011, Mincome (Canada) | Mental health (records data) | Hospital separations, non-congenital mental health | Manitoba Population Health Research Data Repository | ITS model, 6-month intervals from 1970 to 1985 |
| Forget 2013, Mincome (Canada) | Mental health (records data) | Hospital separations (mental health diagnoses) | Manitoba Health Services Insurance Plan registry | 6-month periods beginning 1974 |
| Lassander 2021, Finnish BI Experiment | Subjective financial well-being | Subjective financial well-being (SFWB) - financial stress | Study survey data | 2 years |
| Lassander 2021, Finnish BI Experiment | Subjective financial well-being | Subjective financial well-being (SFWB) - financial management/control | Study survey data | 2 years |
| Lassander 2021, Finnish BI Experiment | Subjective financial well-being | Subjective financial well-being (SFWB) - financial freedom | Study survey data | 2 years |
| Lassander 2021, Finnish BI Experiment | Subjective financial well-being | Subjective financial well-being (SFWB) - emergency funds | Study survey data | 2 years |
| Middleton 1977, New Jersey, USA | Life satisfaction | Quality of life and aspirations | Modified from Cantril (1965) | Unclear |
| Middleton 1977, New Jersey, USA | Mental health (self-reported, single item) | Psychosomatic and nervous symptoms | Based on items appearing in Star (1950); Gurin, Veroff, and Feld (1960); Srole et al. (1962); Leighton et al. (1963); and Langner (1962 | Unclear |
| Middleton 1977, New Jersey, USA | Mental health (self-reported, single item) | General happiness | Developed by researchers | Unclear |
| Middleton 1977, New Jersey, USA | Mental health (self-reported, single item) | Feeling of “nothing to do” | Developed by researchers | Unclear |
| Middleton 1977, New Jersey, USA | Mental health (self-reported, single item) | Self-esteem | Modified from Rosenberg (1965) | Unclear |
| Middleton 1977, New Jersey, USA | Outlook | Community efficacy | Developed by researchers | Unclear |
| Middleton 1977, New Jersey, USA | Outlook | Expectation of better job in future | Developed by researchers | Unclear |
| Middleton 1977, New Jersey, USA | Outlook | Anomy | From McClosky and Schaar (1965) | Unclear |
| Middleton 1977, New Jersey, USA | Outlook | Control of future | From Strodtbeck (1958] | Unclear |
| Middleton 1977, New Jersey, USA | Worries | Worry about money | Modified from Bradburn and Caplovitz (1965) | Unclear |
| Middleton 1977, New Jersey, USA | Worries | Worry about own health | Modified from Bradburn and Caplovitz (1965) | Unclear |
| Middleton 1977, New Jersey, USA | Worries | Worry about health of wife and children | Modified from Bradburn and Caplovitz (1965) | Unclear |
| Middleton 1977, New Jersey, USA | Worries | Worry about raising children | Modified from Bradburn and Caplovitz (1965) | Unclear |
| Middleton 1977, New Jersey, USA | Worries | Worry about losing job | Modified from Bradburn and Caplovitz (1965) | Unclear |
| Muffels 2021, Dutch (Netherlands) | Life satisfaction | Life satisfaction and subjective well-being (self-rated, 0-10 scale) | Study survey data | 16-24 months |
| Muffels 2021, Dutch (Netherlands) | Mental health (self-reported, composite score) | Mental health index (MHI-5) | MHI-5 | 16-24 months |
| Muffels 2021, Dutch (Netherlands) | Subjective financial well-being | Financial stress and poverty | 5-item question | 16-24 months |
| Simanainen 2021, Finnish BI Experiment | Cognitive function | Ability to concentrate (self-rated) | Single item (1-5 scale) | Unclear |
| Simanainen 2021, Finnish BI Experiment | Cognitive function | Memory (self-rated) | Single item (1-5 scale) | Unclear |
| Simanainen 2021, Finnish BI Experiment | Cognitive function | Learning (self-rated) | Single item (1-5 scale) | Unclear |
| Simanainen 2021, Finnish BI Experiment | Life satisfaction | General life satisfaction (self-rated) | Single item (0-10 scale) | Unclear |
| Simanainen 2021, Finnish BI Experiment | Mental health (self-reported, composite score) | Clinical mental distress (MHI-5 score below 53/100) | MHI-5 | Unclear |
| Simanainen 2021, Finnish BI Experiment | Mental health (self-reported, single item) | Being very nervous over the last 4 weeks | MHI-5 item | Unclear |
| Simanainen 2021, Finnish BI Experiment | Mental health (self-reported, single item) | Feeling so low that nothing could make me feel better over the last 4 weeks | MHI-5 item | Unclear |
| Simanainen 2021, Finnish BI Experiment | Mental health (self-reported, single item) | Feeling peaceful and calm over the last 4 weeks | MHI-5 item | Unclear |
| Simanainen 2021, Finnish BI Experiment | Mental health (self-reported, single item) | Feeling sad and downcast over the last 4 weeks | MHI-5 item | Unclear |
| Simanainen 2021, Finnish BI Experiment | Mental health (self-reported, single item) | Being happy over the last 4 weeks | MHI-5 item | Unclear |
| Simanainen 2021, Finnish BI Experiment | Mental health (self-reported, single item) | Experiencing loneliness | Study survey data | Unclear |
| Simanainen 2021, Finnish BI Experiment | Mental health (self-reported, single item) | Experiencing depression | Study survey data | Unclear |
| Simanainen 2021, Finnish BI Experiment | Mental health (self-reported, single item) | Experiencing an inability to enjoy | Study survey data | Unclear |
| Thoits 1979, SIME-DIME, USA | Mental health (self-reported, composite score) | Psychological distress score | Adapted Macmillan Health Survey | Males: 4 months, 20 months; females: 8 months, 24 months |
| Todeschini 2019, B-Mincome (Barcelona) | Life satisfaction | General satisfaction with life (self-rated, 0-10 scale) | Study survey data | Baseline, 11-12 months, 21 months (July 2019) |
| Todeschini 2019, B-Mincome (Barcelona) | Life satisfaction | Being very satisfied with their life | Study survey data | Baseline, 11-12 months, 21 months (July 2019) |
| Todeschini 2019, B-Mincome (Barcelona) | Mental health (self-reported, single item) | Probability of developing a mental disorder (self-reported) | GHQ12 | Baseline, 11-12 months, 21 months (July 2019) |
| Todeschini 2019, B-Mincome (Barcelona) | Mental health (self-reported, single item) | New diagnostics of anxiety and depression | Health care records | Baseline, 11-12 months, 21 months (July 2019) |
| Todeschini 2019, B-Mincome (Barcelona) | Subjective financial well-being | Satisfaction with economic situation (0-10 scale) | Study survey data | Baseline, 11-12 months, 21 months (July 2019) |
| West 2021, SEED, USA | Mental health (self-reported, composite score) | Psychological distress score | Kessler 10 | Baseline (-3 months), Month 1, Month 6, Month 12 |
| West 2021, SEED, USA | Mental health (self-reported, composite score) | Emotional health (Kessler 10 subscale) | Short Form Health Survey 36 (SF-36) | Baseline (-3 months), Month 1, Month 6, Month 12 |
| West 2021, SEED, USA | Mental health (self-reported, composite score) | Emotional well-being (Kessler 10 subscale) | Short Form Health Survey 36 (SF-36) | Baseline (-3 months), Month 1, Month 6, Month 12 |

### 5.4 Social outcomes

| **Study/experiment** | **Outcome sub-category** | **Outcome** | **Instrument/scale/source of data** | **Timing of assessment** |
| --- | --- | --- | --- | --- |
| Calnitsky 2021, Mincome (Canada) | Anti-social behavior | Total crime rates | Uniform Crime Report data (UCR) | annual |
| Calnitsky 2021, Mincome (Canada) | Anti-social behavior | Violent crime rates | Uniform Crime Report data (UCR) | annual |
| Calnitsky 2021, Mincome (Canada) | Anti-social behavior | Property crime rates | Uniform Crime Report data (UCR) | annual |
| Calnitsky 2021, Mincome (Canada) | Anti-social behavior | Other crime rates | Uniform Crime Report data (UCR) | annual |
| Groeneveld 1979, SIME-DIME, USA | Anti-social behavior | Probability of delinquency - status offences | Police records/survey data | 48/60 months |
| Groeneveld 1979, SIME-DIME, USA | Anti-social behavior | Probability of delinquency - serious offences | Police records/survey data | 48/60 months |
| Ladinsky 1977, New Jersey, USA | Social engagement | Social Integration – giving financial aid to friend or relative | Study survey data | Baseline, 4th quarter, 8th quarter |
| Ladinsky 1977, New Jersey, USA | Social engagement | Social Integration – social visits | Study survey data | Baseline, 4th quarter, 8th quarter, 12th quarter |
| Ladinsky 1977, New Jersey, USA | Social engagement | Social Integration – husband-wife | Study survey data | Baseline, 5th quarter |
| Ladinsky 1977, New Jersey, USA | Social engagement | Social Integration – family | Study survey data | Baseline, 5th quarter |
| Ladinsky 1977, New Jersey, USA | Social engagement | Social Integration – membership in organizations | Study survey data | Baseline, 5th quarter, 9th quarter |
| Ladinsky 1977, New Jersey, USA | Social engagement | Social Integration – attendance at religious services | Study survey data | Baseline, 7th quarter |
| Muffels 2021, Dutch (Netherlands) | Social perceptions | Perceived extent of social integration | Study survey data | 16-24 months |
| Muffels 2021, Dutch (Netherlands) | Social perceptions | Social trust | Study survey data | 16-24 months |
| Todeschini 2019, B-Mincome (Barcelona) | Social engagement | Social participation | Study survey data | Baseline, 11-12 months, 21 months (July 2019) |
| Todeschini 2019, B-Mincome (Barcelona) | Social engagement | Volunteering activities | Study survey data | Baseline, 11-12 months, 21 months (July 2019) |
| Todeschini 2019, B-Mincome (Barcelona) | Social engagement | Electoral participation | Study survey data | Baseline, 11-12 months, 21 months (July 2019) |
| Todeschini 2019, B-Mincome (Barcelona) | Social engagement | Participation in social leisure | Study survey data | Baseline, 11-12 months, 21 months (July 2019) |
| Todeschini 2019, B-Mincome (Barcelona) | Social perceptions | Social support and stress - Duke Scale | Duke Scale | Baseline, 11-12 months, 21 months (July 2019) |
| Todeschini 2019, B-Mincome (Barcelona) | Social perceptions | Confidence support | Study survey data | Baseline, 11-12 months, 21 months (July 2019) |
| Todeschini 2019, B-Mincome (Barcelona) | Social perceptions | Emotional support | Study survey data | Baseline, 11-12 months, 21 months (July 2019) |
| Todeschini 2019, B-Mincome (Barcelona) | Social perceptions | Total perceived support | Study survey data | Baseline, 11-12 months, 21 months (July 2019) |

### 5.5 Education/training outcomes

| **Study/experiment** | **Outcome sub-category** | **Outcome** | **Instrument/scale/source of data** | **Timing of assessment** |
| --- | --- | --- | --- | --- |
| Forget 2011, Mincome (Canada) | School continuation | Grade 11/12 Enrolment | Department of Education | Unclear |
| Mallar 1977, New Jersey, USA | School continuation | Probability of high school completion | Study survey data | 3 years (end of the experiment) |
| Mallar 1977, New Jersey, USA | School continuation | Years of Schooling Attained | Study survey data | 3 years (end of the experiment) |
| Mallar 1977, New Jersey, USA | School continuation | College attendance | Study survey data | 3 years (end of the experiment) |
| Manheim 1979, SIME-DIME, USA | Absenteeism | School absences | School records and survey data | Seattle 1972-73; Denver 1973-74 |
| Manheim 1979, SIME-DIME, USA | Academic performance | Grade point average | School records and survey data | Seattle 1972-73; Denver 1973-74 |
| Manheim 1979, SIME-DIME, USA | Academic performance | Standardized test score | School records and survey data | Seattle 1972-73; Denver 1973-74 |
| Maynard 1977, RIME, USA | Absenteeism | Absenteeism | Unclear | “at the time of the most recent observations on any school performance measure” |
| Maynard 1977, RIME, USA | Academic performance | Academic Grade Point Average | Unclear | “at the time of the most recent observations on any school performance measure” |
| Maynard 1977, RIME, USA | Academic performance | Deviation from expected grade equivalent score on standardized achievement test | Unclear | “at the time of the most recent observations on any school performance measure” |
| Maynard 1977, RIME, USA | Academic performance | Standardized achievement test score - Percentile Score | Unclear | “at the time of the most recent observations on any school performance measure” |
| Maynard 1977, RIME, USA | Comportment | Comportment grade point average | Unclear | “at the time of the most recent observations on any school performance measure” |
| Maynard 1979, Gary, USA | Absenteeism | Days absent | Unclear | Baseline, year 1, year 2, year 3 or 4 |
| Maynard 1979, Gary, USA | Academic performance | Reading test score | Unclear | Baseline, year 1, year 2, year 3 or 4 |
| Maynard 1979, Gary, USA | Academic performance | Academic grade point average | Unclear | Baseline, year 1, year 2, year 3 or 4 |
| McDonald 1979, Gary, USA | School continuation | School enrollment | Study survey data | 3rd periodic interview, 2nd school year |
| Todeschini 2019, B-Mincome (Barcelona) | Academic performance | Repeating course (grades 17/18 and 18/19) | Consorci Educació de Barcelona (CEB) | Baseline, 11-12 months, 21 months (July 2019) |
| Todeschini 2019, B-Mincome (Barcelona) | School continuation | Continuing into post-mandatory education | Consorci Educació de Barcelona (CEB) | Baseline, 11-12 months, 21 months (July 2019) |
| Todeschini 2019, B-Mincome (Barcelona) | Skills development | Number of persons in the household doing training | Study survey data | Baseline, 11-12 months, 21 months (July 2019) |
| Venti 1984, SIME-DIME, USA | School continuation | Probability of Schooling | Study survey data | “three points in time” after start of benefits |

### 5.6 Individual choice and agency outcomes

| **Study/experiment** | **Outcome sub-category** | **Outcome** | **Instrument/scale/source of data** | **Timing of assessment** |
| --- | --- | --- | --- | --- |
| Calnitsky 2019, Mincome (Canada) | Choice/agency | Reason for not working – any reason | Study survey data | Baseline, 1 year, and 7 more times every four months |
| Calnitsky 2019, Mincome (Canada) | Choice/agency | Reason for not working – family | Study survey data | Baseline, 1 year, and 7 more times every four months |
| Calnitsky 2019, Mincome (Canada) | Choice/agency | Reason for not working – job/work conditions | Study survey data | Baseline, 1 year, and 7 more times every four months |
| Calnitsky 2019, Mincome (Canada) | Choice/agency | Reason for not working – unpaid vacation | Study survey data | Baseline, 1 year, and 7 more times every four months |
| Calnitsky 2019, Mincome (Canada) | Choice/agency | Reason for not working – education | Study survey data | Baseline, 1 year, and 7 more times every four months |
| Calnitsky 2019, Mincome (Canada) | Choice/agency | Reason for not working – did not want to work | Study survey data | Baseline, 1 year, and 7 more times every four months |
| Calnitsky 2019, Mincome (Canada) | Choice/agency | Reason for not working – ill or disabled | Study survey data | Baseline, 1 year, and 7 more times every four months |
| Calnitsky 2019, Mincome (Canada) | Choice/agency | Reason for not working – self-employed | Study survey data | Baseline, 1 year, and 7 more times every four months |
| Calnitsky 2019, Mincome (Canada) | Choice/agency | Reason for not working – retired | Study survey data | Baseline, 1 year, and 7 more times every four months |
| Calnitsky 2019, Mincome (Canada) | Choice/agency | Reason for not working – other/unknown | Study survey data | Baseline, 1 year, and 7 more times every four months |
| Gonalons-Pons 2021, Mincome (Canada) | Agency (wife) | Bargaining and decision-making power (“voice”) - on wife’s job | Couples Survey | Baseline, 2 years |
| Gonalons-Pons 2021, Mincome (Canada) | Agency (wife) | Bargaining and decision-making power (“voice”) - on important decisions | Couples Survey | Baseline, 2 years |
| Gonalons-Pons 2021, Mincome (Canada) | Agency (wife) | Bargaining and decision-making power (“voice”) - who wins out | Couples Survey | Baseline, 2 years |
| Gonalons-Pons 2021, Mincome (Canada) | Agency (wife) | Financial disagreement index (marital conflict/“loyalty”) | Couples Survey | Baseline, 2 years |
| Gonalons-Pons 2021, Mincome (Canada) | Agency (wife) | Financial disagreement (marital conflict/“loyalty”) - have enough money | Couples Survey | Baseline, 2 years |
| Gonalons-Pons 2021, Mincome (Canada) | Agency (wife) | Financial disagreement (marital conflict/“loyalty”) - save or spend | Couples Survey | Baseline, 2 years |
| Gonalons-Pons 2021, Mincome (Canada) | Agency (wife) | Non-financial disagreement index (marital conflict/“loyalty”) | Couples Survey | Baseline, 2 years |
| Gonalons-Pons 2021, Mincome (Canada) | Agency (wife) | Non-financial disagreement (marital conflict/“loyalty”) - husband’s habits | Couples Survey | Baseline, 2 years |
| Gonalons-Pons 2021, Mincome (Canada) | Agency (wife) | Non-financial disagreement (marital conflict/“loyalty”) - religious beliefs | Couples Survey | Baseline, 2 years |
| Gonalons-Pons 2021, Mincome (Canada) | Agency (wife) | Non-financial disagreement (marital conflict/“loyalty”) - choice of friends | Couples Survey | Baseline, 2 years |
| Ladinsky 1977, New Jersey, USA | Use of time - recreation and entertainment | Leisure Activities – parks and zoos, movies, restaurants, and bars | Study survey data | Baseline, 3rd quarter, 7th quarter, 11th quarter, |
| Ladinsky 1977, New Jersey, USA | Use of time - recreation and entertainment | Leisure Activities – involvement in hobbies, sports activities, and vacations | Study survey data | Baseline, 7th quarter, 11th quarter, |
| Muffels 2021, Dutch (Netherlands) | Choice/agency | Perceived capabilities, freedom of choice | 7-item question | 16-24 months |
| Todeschini 2019, B-Mincome (Barcelona) | Use of time - recreation and entertainment | Participation in individual leisure | Study survey data | Baseline, 11-12 months, 21 months (July 2019) |

## Appendix 6. Excluded poverty-related outcomes and non-relevant outcomes

| **Study/experiment** | **Category** | **Outcome** |
| --- | --- | --- |
| Calnitsky 2019, Mincome (Canada) | Other | Reason for not working – laid off |
| Elesh 1977, New Jersey, USA | Physical health | Number of work days lost (husband and wife) |
| Elesh 1977, New Jersey, USA | Physical health | Total physician visits (husband and wife) |
| Elesh 1977, New Jersey, USA | Physical health | private physician visits (husband and wife) |
| Elesh 1977, New Jersey, USA | Physical health | other physician visits (husband and wife) |
| Elesh 1977, New Jersey, USA | Physical health | Total physician visits (children) |
| Elesh 1977, New Jersey, USA | Physical health | private physician visits (children) |
| Elesh 1977, New Jersey, USA | Physical health | other physician visits (children) |
| Forget 2011, Mincome (Canada) | Other | Proportion of Women with at Least One Child by Age 25 |
| Forget 2011, Mincome (Canada) | Other | Mean Number of Children before Age 25 by Mother’s Birth Cohort |
| Forget 2011, Mincome (Canada) | Other | Family dissolution |
| Forget 2011, Mincome (Canada) | Physical health | Physician visits |
| Gonalons-Pons 2021, Mincome (Canada) | Other | Couple Splits (separation/“exit”) |
| Gonalons-Pons 2021, Mincome (Canada) | Other | Divorce talk frequency (separation/“exit”) |
| Gonalons-Pons 2021, Mincome (Canada) | Other | Wives’ temporary break-ups (separation/“exit”) |
| Kaluzny 1979, Gary, USA | Other | Probability of moving |
| Kerachsky 1977, New Jersey, USA | Physical health | Number of Physician Visits - Adult Males |
| Kerachsky 1977, New Jersey, USA | Physical health | Number of Clinic Visits - Adult Males |
| Kerachsky 1977, New Jersey, USA | Physical health | Visit to Specialist - Adult Males |
| Kerachsky 1977, New Jersey, USA | Physical health | Number of Dentist Visits - Adult Males |
| Kerachsky 1977, New Jersey, USA | Physical health | Number of Days in Bed - Adult Males |
| Kerachsky 1977, New Jersey, USA | Physical health | Number of Days Not Working - Adult Males Only |
| Kerachsky 1977, New Jersey, USA | Physical health | Number of Physician Visits - Adult Females |
| Kerachsky 1977, New Jersey, USA | Physical health | Number of Clinic Visits - Adult Females |
| Kerachsky 1977, New Jersey, USA | Physical health | Visit to Specialist - Adult Females |
| Kerachsky 1977, New Jersey, USA | Physical health | Number of Dentist Visits - Adult Females |
| Kerachsky 1977, New Jersey, USA | Physical health | Number of Days in Bed - Adult Females |
| Ladinsky 1977, New Jersey, USA | Other | Mass media exposure – tv |
| Ladinsky 1977, New Jersey, USA | Other | Mass media exposure – newspaper and magazine reading |
| Mallar 1977, New Jersey, USA | Economic/material | Labor-Supply Activity of Youths (Predicted Marginal Effects in Middle Two Years) |
| Mallar 1977, New Jersey, USA | Economic/material | Labor Force Participation of Youths (Predicted Marginal Effects Evaluated at Appropriate Probabilities) |
| McDonald 1979, Gary, USA | Economic/material | Labor-force participation by youths |
| Middleton 1977, New Jersey, USA | Mental/psychological health | Support government social programs |
| Muffels 2021, Dutch (Netherlands) | Economic/material | Employment outcomes (fulltime, partime, temporary) |
| Muffels 2021, Dutch (Netherlands) | Other | Self-efficacy in finding work |
| Muffels 2021, Dutch (Netherlands) | Other | Job search efforts |
| Muffels 2021, Dutch (Netherlands) | Other | Trust in case worker |
| Simanainen 2021, Finnish BI Experiment | Physical health | Use of health services: Public Health Nurse |
| Simanainen 2021, Finnish BI Experiment | Physical health | Use of health services: Hospital physician |
| Simanainen 2021, Finnish BI Experiment | Physical health | Use of health services: Dentist |
| Simanainen 2021, Finnish BI Experiment | Physical health | Use of health services: Other health care services |
| Todeschini 2019, B-Mincome (Barcelona) | Economic/material | Labor participation (using survey data) |
| Todeschini 2019, B-Mincome (Barcelona) | Economic/material | Using social services |
| Todeschini 2019, B-Mincome (Barcelona) | Economic/material | Number of people working in the household |
| Todeschini 2019, B-Mincome (Barcelona) | Economic/material | Having an indefinite full-time job |
| Todeschini 2019, B-Mincome (Barcelona) | Economic/material | Engaging in entrepreneurship |
| Todeschini 2019, B-Mincome (Barcelona) | Economic/material | Receiving housing subsidy |
| Todeschini 2019, B-Mincome (Barcelona) | Economic/material | Receiving discretionary transfer from municipal social services |
| Todeschini 2019, B-Mincome (Barcelona) | Economic/material | Receiving the RGC |
| Todeschini 2019, B-Mincome (Barcelona) | Other | Use of time - household common tasks |
| Todeschini 2019, B-Mincome (Barcelona) | Physical health | Prescription of painkillers |
| Venti 1984, SIME-DIME, USA | Economic/material | Probability of Working |
| Venti 1984, SIME-DIME, USA | Education/training | Joint probability of neither attending school nor working |
| West 2021, SEED, USA | Economic/material | Aggregate spending data |
| West 2021, SEED, USA | Economic/material | Employment |
