# Supplementary material for: Effects of guaranteed basic income interventions on poverty‐related outcomes in high‐income countries: A systematic review and meta‐analysis
Source: Campbell Syst Rev. 2024 Jun 16;20(2):e1414. doi: 10.1002/cl2.1414 (PMC11180702; doi:10.1002/cl2.1414)
Supplement: Supplementary file 2 — Supporting information. [file CL2-20-e1414-s001.docx]

# Characteristics of studies

## Characteristics of included studies [ordered by study ID]

#### Bonilla 2019

***Study characteristics***

Notes

#### Calnitsky 2019

***Study characteristics***

Notes

#### Calnitsky 2021

***Study characteristics***

Notes

#### Elesh 1977

***Study characteristics***

Notes

#### Forget 2011/2013

***Study characteristics***

Notes

#### Gonalons-Pons 2021

***Study characteristics***

Notes

#### Groeneveld 1979

***Study characteristics***

Notes

#### Kaluzny 1979

***Study characteristics***

Notes

#### Kehrer 1979

***Study characteristics***

Notes

#### Kerachsky 1977

***Study characteristics***

Notes

#### Ladinsky 1977

***Study characteristics***

Notes

#### Lassander 2021

***Study characteristics***

Notes

#### Mallar 1977

***Study characteristics***

Notes

#### Manheim 1979

***Study characteristics***

Notes

#### Maynard 1977

***Study characteristics***

Notes

#### Maynard 1979

***Study characteristics***

Notes

#### McDonald 1979

***Study characteristics***

Notes

#### McIntyre 2016a

***Study characteristics***

Notes

#### Middleton 1977

***Study characteristics***

Notes

#### Muffels 2021

***Study characteristics***

Notes

#### Nicholson 1977

***Study characteristics***

Notes

#### O’Connor 1979

***Study characteristics***

Notes

#### Simanainen 2021

***Study characteristics***

Notes

#### Thoits 1979

***Study characteristics***

Notes

#### Todeschini 2019

***Study characteristics***

Notes

#### Venti 1984

***Study characteristics***

Notes

#### West 2021

***Study characteristics***

Notes

## Characteristics of excluded studies [ordered by study ID]

| Study | Reason for exclusion |
| --- | --- |
| Calnitsky 2017 | Ambiguous poverty-related outcome (employment) |
| Choudhry 1995 | Ambiguous poverty-related outcome (marital dissolution) |
| Choudhry 2001 | Ambiguous poverty-related outcome (household transition dynamics) |
| Cogan 1983 | Ambiguous poverty-related outcome (employment) |
| Greenberg 1983 | Ambiguous poverty-related outcome (employment) |
| Groeneveld 1980 | Ambiguous poverty-related outcome (marital dissolution) |
| Hannan 1977 | Ambiguous poverty-related outcome (marital dissolution) |
| Heffernan 1977 | Ambiguous poverty-related outcome (awareness of social services) |
| Hum 1992 | Ambiguous poverty-related outcome (marital dissolution) |
| Keeley 1980a | Ambiguous poverty-related outcome (migration) |
| Keeley 1980b | Ambiguous poverty-related outcome (fertility) |
| Keeley 1987 | Ambiguous poverty-related outcome (marital dissolution) |
| McDowell 2020 | Ineligible study design (cross-sectional with one timepoint) |
| McDowell 2021 | Ineligible study design (cross-sectional with one timepoint) |
| McIntyre 2016b | Ineligible study design (cross-sectional with one timepoint) |
| Pencavel 1982 | Ambiguous poverty-related outcome (employment) |
| Robins 1980 | Ambiguous poverty-related outcome (employment) |
| West 1980 | Ambiguous poverty-related outcome (employment) |
